# Disaster Preparedness Intervention for Older Adults (Seniors’ Positive Involvement in Community Emergencies): Protocol for a Quasi-Experimental Study

**DOI:** 10.2196/58895

**Published:** 2024-12-04

**Authors:** Sharon White-Lewis, Joseph Lightner, Julia Crowley, Amanda Grimes, Kathleen Spears, Steven Chesnut

**Affiliations:** 1 School of Nursing and Health Sciences University of Missouri Kansas City Kansas City, MO United States; 2 School of Medicine University of Missouri Kansas City St. Joseph, MO United States

**Keywords:** older adults, disaster preparedness, emergency preparedness, disaster protocol, disaster engagement, disaster recovery, personal preparedness, community dwelling older adults, elderly, aging in place, activities in daily life, resiliency, community health

## Abstract

**Background:**

Older adults comprise a substantial proportion of the US population requiring support during disaster events. Previous research demonstrates that older adults are resilient but deficient in disaster preparedness and lacking in community engagement. There is a gap in high-quality research in this area.

**Objective:**

This study aims to fill this gap by developing a 4-phase intervention to improve mobility and balance, decrease fall risks (mitigation), increase knowledge of disaster preparedness (preparedness), improve community emergency operation plans (response), and improve self-efficacy in disaster recovery (recovery) for older adults.

**Methods:**

This is a community-based, 10-month study in a large Midwestern urban and suburban location targeting community-dwelling older adults. The 4 phases of interventions address mitigation, preparedness, response, and recovery—aspects improving outcomes from disaster events. In total, 4 to 6 one-hour seminars each month are provided to community-dwelling older adults to improve disaster preparedness and recovery planning. A critical incident packet with resources on essential information such as medications, a communication plan, evacuation resources, and supplies was started and is being reviewed. Preintervention surveys are orally given, with research assistants aiding in any difficulties the participants have. After the surveys, 2 individual 20-minute presentations separated by a short break for snacks and initial completion of their disaster plan preserve the older adult’s attention. Mitigation efforts to improve mobility and safety are offered with 10 visits to the older adults’ residences, adapting physical activity and balance exercises to the individual’s needs. To address response needs, the emergency operations plans for 2 of the major cities are being amended for specific functional needs and access guidelines. Measurements include accelerometers to assess improvement in mobility, fall risk assessments, an abbreviated Federal Emergency Management Association Household Survey, an assessment for disaster engagement with partners tool, a brief pain inventory assessment, and the General Self-Efficacy Scale. We analyze data descriptively and compare pre- and postintervention data for each phase with paired-samples *t* test and other nonparametric techniques (proportion tests and Wilcoxon signed-rank tests). Overarching objectives prioritized during this intervention include underscoring respect for the experience and resilience found in older adults and engaging them in specialized roles to support their communities during disaster events.

**Results:**

The intervention was funded in July 2023; enrollment began in November 2023 and is continuing. We will conclude data collection by July 2025. Published study results can be expected in early 2025.

**Conclusions:**

With improved disaster preparedness, mobility, recovery planning, and inclusion as a resource in community disasters, older adults are expected to be safer and be able to age in place. If successful, future studies will focus on outreach and sustainability. This study will serve as a model for older adult disaster preparedness and community involvement.

**International Registered Report Identifier (IRRID):**

DERR1-10.2196/58895

## Introduction

Older adults experiencing disaster events are disproportionately at risk for morbidity and mortality [1,2]. This population is increasing to 1 in 5 Americans by 2050 [3], with 39.5% needing equipment to perform activities of daily life [4]. The Federal Emergency Management Association (FEMA) Household Survey on disaster preparedness [5] reported a disconnect between the older adult population’s perception of preparedness and their preparedness actions, prompting FEMA to urge communities and emergency managers to focus on and empower this population to meet their unique situations and challenges before disasters. FEMA’s survey results found a lack of planning for disasters, preparing for evacuation, family communication, participation in drills, and involvement with their community and neighbors among American older adults. Evidence suggests that while older adults are more resilient in disasters [6], they are also more isolated [7]. The National Academies of Sciences [8] reports isolation is exacerbated during disaster events, recommending improved connection with communities. Due to time horizons (the sense of how much time is left in life), older adults are uniquely susceptible to disaster mental health issues [9]. Mental health and lack of access to medical care post disaster require specific attention for this population. After Hurricane Sandy, Corley et al [10] found that older adults had increased anxiety, stress, and posttraumatic stress disorder.

Current risk management efforts focus on preparedness, mitigation, and planning for recovery, along with acute response to the disaster event [3]. While literature is scarce and dated on interventions that improve disaster preparedness and self-efficacy, Ashida et al [11] noted some convincing evidence of positive outcomes from postpreparedness education. Pre-event decisions should be planned before the disaster event to improve peace of mind and individual response times [12]. In addition, this population is motivated by a need to feel valued rather than expendable [13]. Older adults have had experience with disasters, wars, and challenges in their lives, expertise which may be reflected in the data supporting their increased resilience in emergencies [14,15]. They provide experience from previous successes or learning from previous failures, giving value to the older adults and hope to survivors. There are 1 primary and 4 secondary hypotheses for this study. The primary hypothesis is that the interventions will show significant improvement in disaster preparedness knowledge for community-dwelling older adults from the pre- to postintervention survey. This will be supported by four subhypotheses: (1) mitigation: the intervention will demonstrate improved mobility and safety from before to after the intervention; (2) preparedness: the seminar interventions will demonstrate significant improvement in household disaster readiness measured by the FEMA Household Survey [5] from before to after intervention; (3) response: the collaboration between supporting disaster management organizations will improve interagency teamwork and communication after the intervention as measured by the Assessment for Disaster Engagement with Partners Tool (ADEPT); and (4) recovery: the self-efficacy of community-dwelling older adults will statistically improve from before to after the intervention.

## Methods

### Study Design

#### Overview

This is a 4-phase, quasi-experimental study conducted in a Midwestern US metropolitan area. Population includes all community-dwelling older adults, defined as those equal to or older than 65 years of age living independently. Support from 12 organizations, including public health departments, emergency management councils, regional support councils, and disaster volunteer organizations, was obtained. Partner organizations provide a host location and recruitment for the seminars through organizational emails, flyers, and word of mouth. Childcare is offered, allowing older adults who are babysitting during daytime hours to attend. The 4 seminars include Spanish interpretation and materials. The option for study involvement is voluntary and private. The 4 phases follow disaster phase principles and are mitigation, preparedness, response, and recovery.

#### Mitigation

To improve mobility and mitigate the loss of life during disasters, we are recruiting a sample of older adults to participate in a 10-week, perspective, pre- and posttest, fall prevention and physical activity intervention.

#### Preparedness

Four seminars per month are being given for 1 hour with a PowerPoint presentation on disaster preparedness, decision making, communication, and county state resources available. The Your Very Personal Preparedness Inventory (Multimedia Appendix 1) [16] begins with assistance from a research assistant, ending with the presentation of a red critical information packet to be stored at their home for emergencies. In addition to the personal preparedness inventory, the critical information packet includes Your Guide to Emergency Preparedness in the Greater Kansas City Region (Multimedia Appendix 2), My Communication for Disaster Recovery (Multimedia Appendix 3), and My Action Steps to Avoid: Disaster Recovery Scams (Multimedia Appendix 4). All are welcome to attend whether they opt in for the study or not. In total, 2 test seminars were held to obtain feedback from participants on the value of the information given and the method of delivery. Feedback included having more interaction and making the information more concrete and less abstract.

The study participants orally consented and then asked for an abbreviated version of the FEMA Household Survey. The seminar presentation (Multimedia Appendix 5) is then presented to the participants. One week after the seminar, the same research assistant phones the participant and asks the same questions. All answers are documented in Qualtrics XM, a secure encrypted data gathering and analyzing program, for confidentiality. Media releases for educational sharing of photos are signed upon entering the seminars.

#### Response

Two city emergency management departments and 8 community support organizations and coalitions were engaged to participate in a review of their current emergency operations plan with a focus on improving integration of older adult needs. The organizations agreed to participate in writing before beginning work with the research team. Research assistants were then provided with copies of organization emergency plans and participated in discussions with stakeholders to create organization-specific recommendation guides based on current literature and community assessments to address identified challenges for older adults. Members of leadership and registered personnel of participating organizations were asked to rate their organization’s level of partner engagement by completing the ADEPT [17] through an email survey before engaging in the plan review and again after the research team presented their recommendations at the close of the project. Responses to both surveys were recorded in Qualtrics XM and compared with measure changes in 4 domains of partner engagement: communication outreach and coordination, resource mobilization, organizational capacity building, and partnership development and maintenance.

#### Recovery

As in the preparedness phase, four 20-minute seminars per month, in conjunction with the preparedness seminars, are also delivered to community-dwelling older adults on disaster recovery. Similarly, this phase of the project underwent 2 test seminars with nearly identical feedback to that of the preparedness phase. A team of research assistants collected oral consent from participants and administered the General Self-Efficacy Scale (GSES) [18] before the seminar. As an intervention study, research assistants re-administer the GSES through phone 1 week after the seminar to examine any changes in perceived self-efficacy pertaining to disaster recovery. Oral responses are recorded in Qualtrics XM by the research assistants.

### Ethical Consideration

All phases were submitted and approved by the University of Missouri-Kansas City institutional review board (2094344, 2097949, 2098365, and 2098527). Preparedness, response, and recovery were deemed exempt since identification of individuals was only for follow-up phone call communication and all data were and are deidentified after the 1-week follow-up phone call. The mitigation research review was expedited and approved due to the length of the intervention being over 2 hours. No compensation other than snacks was given.

### Description of the Intervention

#### Overview

“Seniors’ Positive Impact on Community Emergencies” is a community-based intervention targeting community-dwelling older adults aged 65 years or older. The program’s intent is 3-fold: To improve disaster preparedness for older adults whose independent living does not afford them the protections of extended care facilities, improve community involvement and respect for the known experiential depth of our older populations, and create a safer environment during disaster events by reducing fall hazards in the home. There is an overabundance of literature on the need for specific interventions during disasters [4,12,19,20] but few to no studies conducted on the effect of interventions to improve this situation.

#### Mitigation Programming

At enrollment, participants are provided an accelerometer to assess baseline physical activity for 1 week; research assistants determine a time to visit the participant’s home to collect the accelerometer and to conduct the first home visit. During the first session, research assistants assess home fall hazards using the Fall Prevention Checklist [21]. In response to the checklist, research staff developed a plan with the participant to modify their home environment to prevent fall risks increase mobility for a safe evacuation and have a safer home environment. A balanced assessment is also conducted using the Timed Up and Go test (TUG) [22]. In addition, research assistants and participants develop small, realistic physical activity goals for the 10-week intervention using the Go4Life goal-setting worksheet [23]. Potential goals include doing balance or chair exercises, walking around their home, and walking outside for 10-30 minutes. A research assistant goes to the participant’s home once per week for 1 hour to exercise with the participant. Measures are reassessed at the conclusion of the intervention for a posttest comparison.

#### Preparedness Programming

The 4 seminars per month are given with a 20-minute Microsoft PowerPoint interactive session and a 15-minute time block in which research assistants provide help completing the participants’ critical information packet and individualizing it for their community. The following disaster preparedness subjects are the content included: statistics on the cost and incidence of natural disasters, alerts versus warnings, decisions such as shelter-in-place or evacuation, supplies, disaster pet rescue, communication plans, transportation, medication, important documents, and medical equipment. These subjects all prepare the community-dwelling older adults so that they can act quickly if a disaster occurs. The critical information packet includes the booklet: Your Very Personal Preparedness Inventory [16], Your Guide to Emergency Preparedness in the Greater Kansas City Region [24] from the area emergency managers, and a sheet of resources particular to the area where the seminar is being given. It also includes an individual sheet with contact information or unique disaster details for the participant and their family, and a credit-card-sized magnifier so that all information can be easily viewed. During the breaks, a disaster go-bag with essential supplies is displayed near the refreshments with a list of basic go-bag needs.

#### Response Programming

Upon establishing partner agency enrollment, the researchers begin by introducing the ADEPT survey, explaining its goals, and distributing it electronically to emergency response community members [17]. This provides a baseline of current engagement metrics as reported by agency partners. Researchers then collaborate with emergency managers and community stakeholders to conduct a comprehensive review of current emergency operations plans for the participating municipalities. Research assistants also perform a community needs assessment identifying and verifying current resources for community-dwelling older adult populations and a literature review of evidence-based practices and model emergency operations plans addressing the needs of older adults, allowing guide designers to creatively incorporate older adults as contributing support resources in the construction of their recommendations. Research assistants then present their findings and recommendation guides in training seminar format at designated organizational meetings to inform and educate collaborators. After the presentation of these seminars and deliverables, participating agency leadership and affiliated members are resurveyed with the ADEPT inventory to assess reported engagement changes post intervention.

#### Recovery Programming

The 20-minute recovery seminars include a Microsoft PowerPoint presentation with the following content: defining disaster recovery, discussing common challenges in the recovery phase, explaining the role of FEMA in recovery, and descriptions of local disaster recovery centers, and other community resources. The presentation concludes by reminding participants that they are survivors and not victims in disaster recovery because they have resilience and are assets to their communities. In addition to the PowerPoint presentation, participants will also receive 2 handouts: one that provides information on recommended actions to avoid disaster recovery scams and another that provides communication resources for disaster recovery.

### Measures

#### Demographic Variables

The older adults’ demographic variables are measured by self-reporting questions at baseline. They are asked name and phone number for the follow-up survey access, age, sex, and zip code, worded identically to the FEMA Household Survey [5] questions.

#### Mitigation Measures

Home fall hazards are assessed before and after the intervention using the Fall Prevention Checklist [21]. The Fall Prevention Checklist assesses multiple aspects of a participant’s home, including stairs, loose carpets, handrails, etc. To assess balance before and after the intervention, the TUG assessment is used [22]. The TUG asks participants to get up from a chair, walk to a place marker about 10 feet away, return, and sit back down. The baseline level of physical activity is assessed using a Garmin Vivofit 4 wrist-mounted accelerometer for 1 week before the intervention [25]. At the end of the 10 weeks, the participant is asked to wear the Garmin device again for 1 week to assess physical activity after the intervention.

#### Preparedness Measures

Before and 1 week after the disaster preparedness seminars, an abbreviated version of the FEMA Household Survey [5] is orally administered by research assistants and documented in Qualtrics XM. FEMA Household Survey [5] questions are copied with permission verbatim from the original survey for national norming comparison. To abbreviate the survey to increase acceptance, disasters not appropriate for the Midwest such as tsunamis; types of alerts; and specific disaster questions such as coastal flooding, earthquake, etc, were removed. The time average for taking the oral survey is 13 minutes.

#### Response Measures

Partner engagement is assessed before and after the intervention using the ADEPT instrument [17]. The ADEPT survey assesses the impact on partner engagement relationships using a 15-item set of ordinal scale questions across 4 domains: communication outreach and coordination, resource mobilization, organizational capacity building, and partnership development and maintenance. This tool was validated using internal consistency reliability, and reasonable interitem reliability for the 4 hypothesized dimensions (Cronbach α=.71-.88). These dimensions were confirmed through correlation and factor analysis (Varimax rotation) [17]. Completion time is approximately 15 minutes. A link to the Qualtrics XM survey form is emailed to a roster of affiliated collaborator members at the beginning of the project and again at the end of the project after the presentation of community-dwelling older adult recommendation guides. The survey population includes disaster response organization leadership, members, and stakeholders. The survey form includes an electronic statement of consent and requires participants’ affirmative submission of their data to opt into the study. The survey form also collects demographic data that include the participant’s name, contact phone, and affiliated organization and role. To support response fidelity in a comparatively small sample population, participants receive 1 follow-up email reminder to complete the survey 1 week after distribution and 1 phone call reminder 2 weeks after distribution for each survey. If no response is received from either prompt, an in-person visit from a research assistant may follow to confirm opportunity awareness. The researchers do not use coercive methods to obtain survey responses; participation must remain strictly voluntary. Further, participant identifiers should not be used in analysis reporting and remain undisclosed.

The GSES [18] is a 10-question survey that generates ordinal-level responses pertaining to self-efficacy. Self-efficacy in this context refers to, “a person’s belief in his or her capability to successfully perform a particular task” [26]. After obtaining oral consent from participants, research assistants briefly define the meaning of disaster recovery and explain that participants will answer each question in the context of the particular task of recovering from a disaster. As previously mentioned, participants receive follow-up surveys through phone from the research assistants 1 week after the recovery intervention that are recorded in Qualtrics XM.

Deidentified data collected from these measures may be made available to researchers upon request from the corresponding author after completion of the project.

### Statistical Analysis

Data preparation and analysis will be conducted in R (R Core Team) [27] using a variety of base and add-on libraries available through the Comprehensive R Archive Network [28]. In preparing the data, we will examine missing responses to identify the mechanism most likely responsible for the missingness to determine the appropriateness of multiple imputation procedures for data recovery [29,30]. In analyzing the data, we will examine the descriptive statistics for all measured variables, evaluate the reliability of the instruments used to measure the constructs of interest and perform appropriate cross-sectional and repeated measures analyses to address the aims guiding the project. Specifically, we will examine the associations between constructs measured at each wave using appropriate correlation techniques, such as Pearson, Spearman, or phi correlation, to adequately account for the distributional characteristics of the variables. In addition, we will examine changes to the constructs of interest (eg, knowledge, self-efficacy, physical activity) using repeated measures techniques that account for the scale of measurement (eg, categorical, continuous) and distributional characteristics (eg, approximate normality), such as the paired-samples 2-tailed *t* tests, repeated measures ANOVA, Wilcoxon signed-rank test, or a generalized estimating equation. We will test for statistical significance against an α threshold of .05.

## Results

This study began in November 2023 and will continue through July 2025 (20 months). Trial seminars occurred in December 2023 with the feedback of increasing interaction and delivering concrete actions for disaster response based on location, community, and individual needs. This was incorporated before the study. Results will be communicated to the professional community by publications, and results will also be given in presentation form to stakeholders, organizations, and governmental entities. Published study results can be expected in early 2025.

## Discussion

### Expected Findings

With the older adult population increasing, it is important to support efforts that allow aging in place. This decreases loneliness and provides a sense of security [31,32]. Older adults are more vulnerable during disasters due to mobility, cognitive, and functional needs [1,33,34]. This study tests community-based interventions to improve household disaster literacy and community awareness of these specific needs while using the population’s resilience to improve community response.

Like related studies, we expect to find statistically significant improvements in mobility, preparedness, collaboration between response organizations, community involvement, and self-efficacy will be demonstrated. For example, this project is similar to the Japan Earthquake project, providing support for older adults. Miyadera et al [35] in Japan conducted a quasi-experiment trial of 89 older adults aged 65 years or older that targeted improvements in physical activities in daily life. The older adults’ quality of life was significantly improved (*P*<.01, effect size 0.51) when focused program interventions were administered. Ashida et al [36] studied 194 members in a Midwestern community. After an Emergency Network Form was completed with assistance, they found statistically significant gains in nonfamilial networks and emotional support.

### Limitations and Strengths

Limitations of this study include self-reporting surveys, which are subjective, with no control group to assess if the interventions influenced participants. Randomization was not used in this convenience sample. Isolated community-dwelling older adults are difficult to reach, resulting in a population already prone to action during disasters and not the general population.

Strengths include support from emergency managers, public health departments, disaster response agencies, faith-based organizations, and regional governmental organizations. The overarching respect and noted value of this population in all study endeavors results in community inclusion and interaction. The acceptance and feasibility of this study occurring in their own communities allows participation from community-dwelling older adults that is often overlooked.

### Conclusion

Disaster preparedness of community-dwelling older adults is a priority for many organizations in the United States. Research on the effectiveness of interventions to improve older adults’ safety before, during, and after disasters is lacking. This protocol describes an all-encompassing project to improve postdisaster outcomes by addressing mitigation, preparedness, response, and recovery factors that influence disaster outcomes for this population. Further research needs to be conducted in this area with this population.

## Appendix

Multimedia Appendix 1 Your Very Personal Preparedness Inventory.

Multimedia Appendix 2 Your Guide to Emergency Preparedness in the Greater Kansas City Region.

Multimedia Appendix 3 My Communication for Disaster Recovery.

Multimedia Appendix 4 My Action Steps to Avoid: Disaster Recovery Scams.

Multimedia Appendix 5 Seminar presentation.

## Abbreviations

ADEPT: Assessment for Disaster Engagement with Partners Tool
FEMA: Federal Emergency Management Association
GSES: General Self-Efficacy Scale
TUG: Timed Up and Go test
