# Correction: Disaster Preparedness Intervention for Older Adults (Seniors’ Positive Involvement in Community Emergencies): Protocol for a Quasi-Experimental Study

**DOI:** 10.2196/71160

**Published:** 2025-01-16

**Authors:** Sharon White-Lewis, Joseph Lightner, Julia Crowley, Amanda Grimes, Kathleen Spears, Steven Chesnut

**Affiliations:** 1 School of Nursing and Health Sciences University of Missouri Kansas City Kansas City, MO United States; 2 School of Medicine University of Missouri Kansas City St. Joseph, MO United States

In “Disaster Preparedness Intervention for Older Adults (Seniors’ Positive Involvement in Community Emergencies): Protocol for a Quasi-Experimental Study” JMIR Res Protoc 2024;13:e58895) the authors made one correction.

The last name of author JC, “Crowely”, has been revised to “Crowley”.

The correction will appear in the online version of the paper on the JMIR Publications website on January 16, 2025 together with the publication of this correction notice. Because this was made after submission to PubMed, PubMed Central, and other full-text repositories, the corrected article has also been resubmitted to those repositories.

