# Supplementary material for: Disaster Preparedness Intervention for Older Adults (Seniors’ Positive Involvement in Community Emergencies): Protocol for a Quasi-Experimental Study
Source: JMIR Res Protoc. 2024 Dec 4;13:e58895. doi: 10.2196/58895 (PMC11656111; doi:10.2196/58895)
Supplement: Multimedia Appendix 1 [file resprot_v13i1e58895_app1.pdf]

Your Very Personal Preparedness Inventory:

Assessing the important additional resources you will need before, during and after a disaster.

Community Disaster Resiliency Network

MARC
MID-AMERICA REGIONAL COUNCIL

Margaret A. Cargill Foundation
A MARGARET A. CARGILL PHILANTHROPY

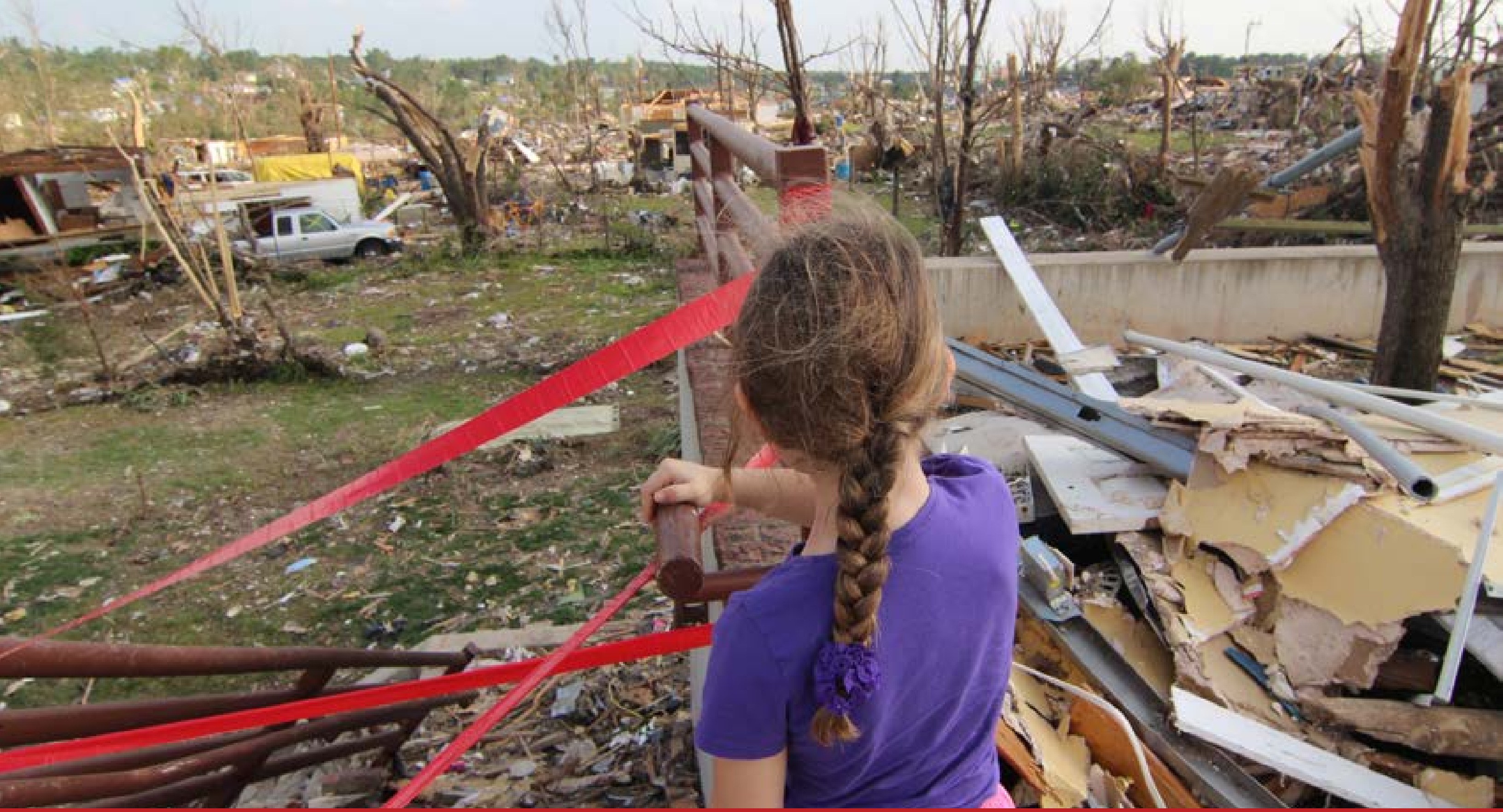

Why plan for disaster?

Disasters can happen anytime and anywhere. Severe thunderstorms, tornadoes, floods, fires, snow and ice storms, and hazardous material spills are all hazards that are real possibilities in our region. When they occur, you may not have much time to respond.

It is important to take steps ahead of time, such as putting an emergency kit together, making plans, and having ways to stay informed. Planning in advance can help you cope with resources which are limited or cut off as a result of a disaster. Local officials or relief workers may not be able to assist you right away. You will need to rely on the people and resources available at the time.

The good news is you have resources around you to help meet your daily needs and carry out your regular activities.

Preparing for disasters includes identifying which supplies, devices, medications and equipment are most important to you and then taking action to make sure they are accessible in an emergency.

Some supplies are standard items that everyone depends on, such as food, water, clothing, lodging, transportation and communication devices. These items should be included in a standard emergency kit.

Remember, this booklet is YOURS! It is also a tool to help other people help you. Once you've completed the sections that apply to you, provide a copy to the people you rely on the most during and after a disaster.

What are your specific needs?

In addition to standard emergency supplies, you may require additional resources to meet your specific needs. Extra consideration must be given to include these additional resources into your emergency preparedness efforts.

Examples include:

- If you rely on a motorized wheelchair or device requiring electrical power to move around, do you have a backup plan if there is a power outage?
- If you take medications to manage a potentially life-threatening medical condition, do you have enough on hand to include some in your emergency kit? Do you have information written down in your emergency kit that will help you get more if needed?
- If you have specific dietary needs, have you included food that you can eat in your emergency kit?

This booklet is meant to help you 1) assess the additional resources you need the most; 2) gather important information about those resources; and 3) identify possible alternative sources.

GET A KIT | MAKE A PLAN | STAY INFORMED

This booklet will help you identify additional supplies that should be in your emergency kit, which resources need to be part of your emergency planning process and which information sources will help you stay informed.

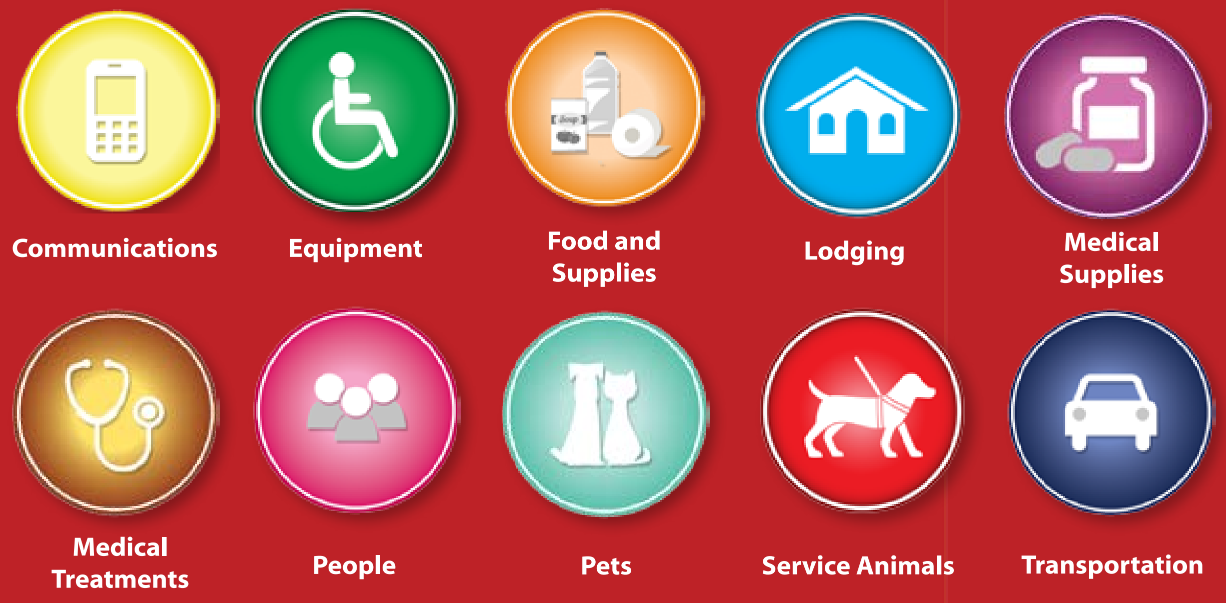

Fill out the sections that are applicable to YOUR needs.

What's important to you?

Before diving into this booklet, take a moment to determine which sections are meaningful to you. Once you have selected the most important areas, this page will help guide you to the appropriate sections to address those topics as they relate to putting your kit together, making a plan and staying informed. In the boxes below, please check all topics important to you.

TOPIC	IMPORTANT TO ME	KIT	PLAN	INFO
		(section page numbers below)		
Communications — getting information about hazards and communicating with others.	☐	6		41
Equipment — durable medical equipment and assistive devices.	☐	7		
Food and supplies — special dietary needs and preferences; important supplies not already in your kit.	☐	10, 19		
Lodging — alternate places to stay; need for assistance at a shelter.	☐		23	
Medication — prescription and over-the-counter medicines.	☐	12		
Medical treatment — regular medical treatments/procedures received.	☐		26	
People — family, friends, physicians, care assistants, translators.	☐		27	
Pets — pet supplies and possible care providers.	☐	17	36	
Service Animals — supplies needed for service animals; contact info for veterinarian.	☐	18		
Transportation — alternate transportation providers.	☐		38	

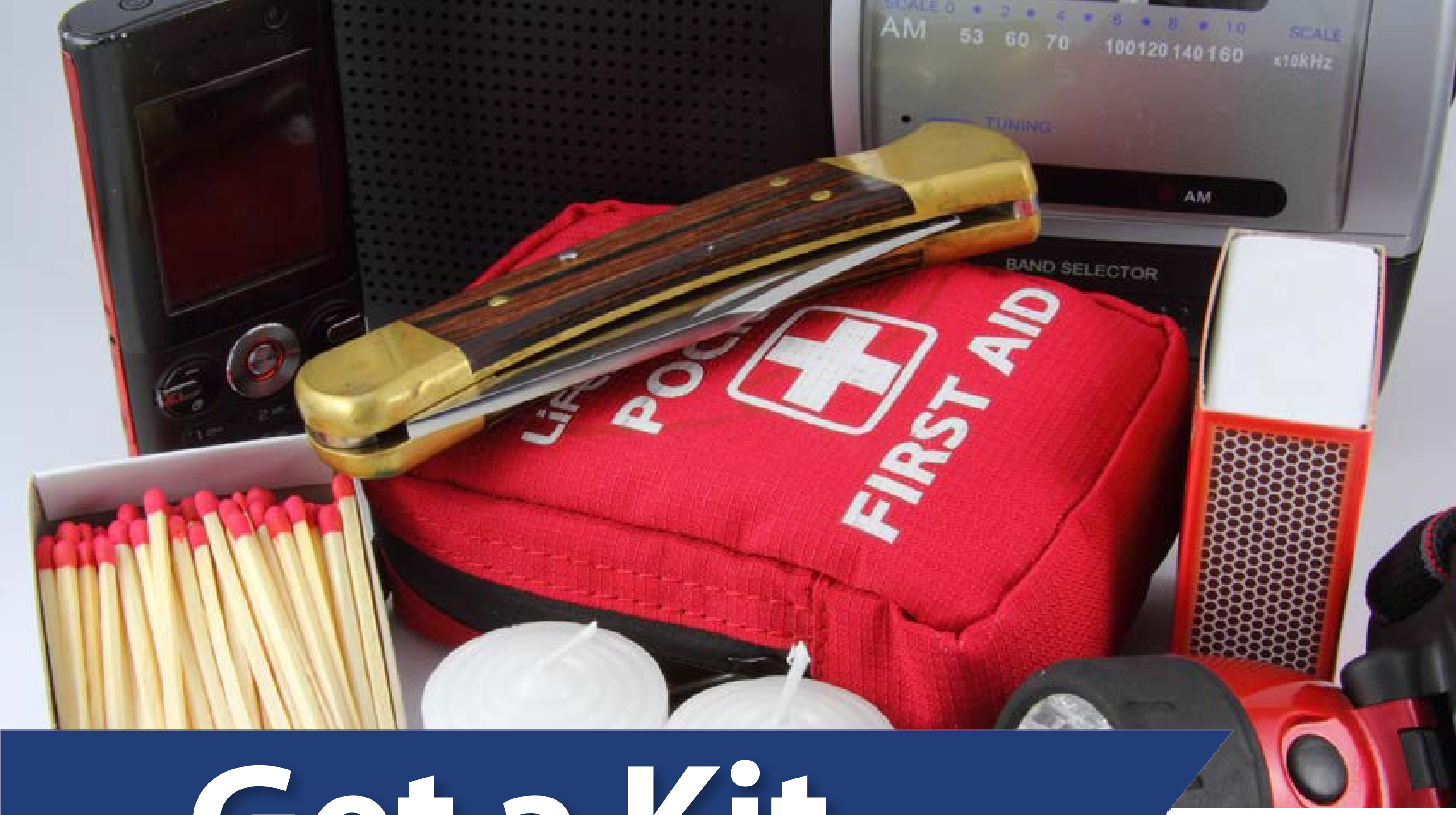

Get a Kit

Action Item!

If you haven't already put together your standard emergency kit, please start immediately. For resources on putting together a kit, go to the following websites:

PrepareMetroKC	preparemetrokc.org/memc-brochure.pdf<br>preparemetrokc.org/Be_Prepared/supplykit.asp
Federal Emergency Management Agency	ready.gov/build-a-kit
American Red Cross	redcross.org/prepare/location/home-family/get-kit
Centers for Disease Control and Prevention	emergency.cdc.gov/preparedness/kit/disasters

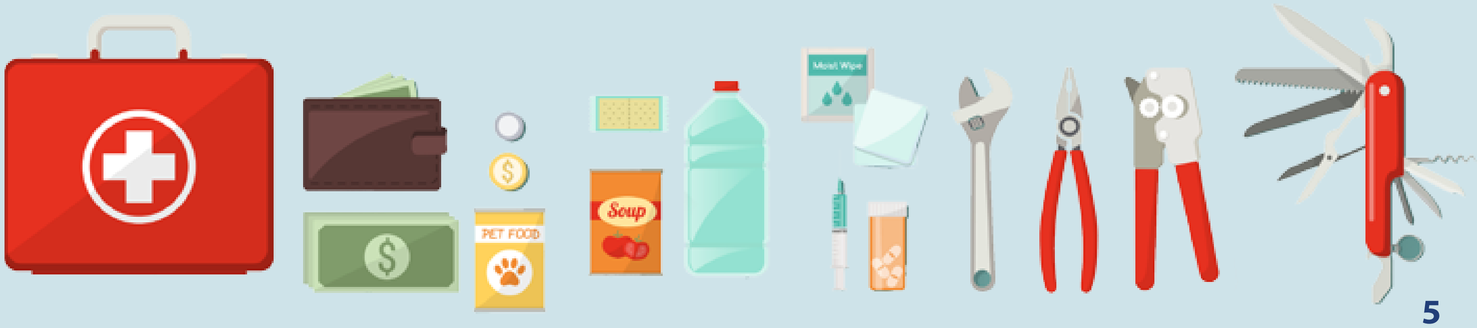

Communications

Communication during and after a disaster is extremely important, but it can sometimes be challenging. You may have a variety of phones, tablets and computers you use to communicate with others. It is good to have more than one type of device in case one is not functioning, such as during a power outage or cell towers being overloaded.

How will you stay in touch?

1. If you use a cell phone, do you have an extra phone charger for your phone? Do you have a car charger or additional way to charge your phone? YES_____ NO_____

    a. Have you included your extra phone charger, car charger or other charging device in your emergency kit? YES_____NO_____

2. Do you use an aided Augmentative and Alternative Communication system, such as electronic tablet, message board or TTY? YES_____NO_____

    a. What is the device? ______________________________

    b. Do you have a backup device in your emergency kit? YES_____NO_____

    c. If your device requires a battery, do you have extra batteries and/or a battery charger in your emergency kit? YES_____NO_____

    d. Who is your service provider? ______________________________

    e. Telephone number of provider: ______________________________

Action Items!

If you answered no to any of the questions above, take action to make sure you have the items needed. List your action items here:

____

____

____

____

Equipment

Do you use specific equipment in your regular everyday activities — to get around, eat, hear, see and read, or breathe easier? If you did not have that equipment, would it affect your ability to function or communicate? Do not assume these items will be provided by emergency responders, relief workers and shelter providers.

What equipment do you need to function?

List the pieces of equipment that are either Extremely Important (vital for life functions) or Important (significantly affects ability to function):

1. Piece of equipment: ____

   a. Do you have this piece of equipment or a duplicate in your emergency kit?
   YES______NO______

   b. Provider that assists you with this equipment: ______________________

   c. Address: ____

   d. Phone number: ____

   e. Does this provider have a plan for providing services after an emergency?
   YES______NO______

   f. Alternate source that could provide assistance with this piece of equipment:
   ____

   g. Address: ____

   h. Phone number: ____

   i. Does this provider have a plan for providing services after an emergency?
   YES______NO______

Equipment, continued

2. Piece of equipment: ____

   a. Do you have this piece of equipment or a duplicate in your emergency kit?
   YES_____NO_____

   b. Provider that assists you with this equipment: ______________________

   c. Address: ____

   d. Phone number: ____

   e. Does this provider have a plan for providing services after an emergency?
   YES_____NO_____

   f. Alternate source that could provide assistance with this piece of equipment:
   ____

   g. Address: ____

   h. Phone number: ____

   i. Does this provider have a plan for providing services after an emergency?
   YES_____NO_____

3. Piece of equipment: ____

   a. Do you have this piece of equipment or a duplicate in your emergency kit?
   YES_____NO_____

   b. Provider that assists you with this equipment: ______________________

   c. Address: ____

   d. Phone number: ____

   e. Does this provider have a plan for providing services after an emergency?
   YES_____NO_____

   f. Alternate source that could provide assistance with this piece of equipment:
   ____

   g. Address: ____

   h. Phone number: ____

   i. Does this provider have a plan for providing services after an emergency?

4. Piece of equipment: ____

a. Do you have this piece of equipment or a duplicate in your emergency kit?
YES______NO______

b. Provider that assists you with this equipment: ______________________

c. Address: ____

d. Phone number: ____

e. Does this provider have a plan for providing services after an emergency?
YES______NO______

f. Alternate source that could provide assistance with this piece of equipment:
____

g. Address: ____

h. Phone number: ____

i. Does this provider have a plan for providing services after an emergency?
YES______NO______

Action Items!

If you answered no to any of the questions above, take action to make sure you have the items needed. List your action items here:

____

____

____

____

____

____

____

____

____

____

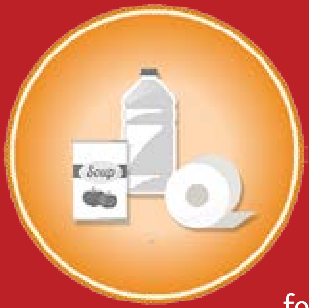

Food

Food is one of the most important things we need. Standard emergency kits recommend non-perishable foods that require no refrigeration, preparation or cooking, such as certain canned foods, high energy foods, like granola bars or peanut butter, and comfort snacks like cookies. If you have special dietary restrictions or food allergies, some standard suggested foods may not be appropriate. Remember, emergency shelters may not be able to accommodate your dietary restrictions.

What foods can you eat?

1. Do you have dietary restrictions or special dietary needs? YES_____NO_____
2. Describe the restrictions: ____

   ____

   a. Do you have documentation in your kit that describes your dietary needs? YES_____NO_____

   b. Does your kit include food that accommodates your dietary restrictions? YES_____NO_____

3. Do you have food allergies? YES_____NO_____
4. If so, please list your allergies here:

**FOOD ALLERGY:**	**Treatment included in emergency kit?** YES_____NO_____
Reaction:	
Treatment:	
**FOOD ALLERGY:**	**Treatment included in emergency kit?** YES_____NO_____
Reaction:	
Treatment:	
**FOOD ALLERGY:**	**Treatment included in emergency kit?** YES_____NO_____
Reaction:	
Treatment:	
**FOOD ALLERGY:**	**Treatment included in emergency kit?** YES_____NO_____
Reaction:	
Treatment:	

**FOOD ALLERGY:**	**Treatment included in emergency kit?** YES_____NO_____
Reaction:	
Treatment:	
**FOOD ALLERGY:**	**Treatment included in emergency kit?** YES_____NO_____
Reaction:	
Treatment:	
**FOOD ALLERGY:**	**Treatment included in emergency kit?** YES_____NO_____
Reaction:	
Treatment:	
**FOOD ALLERGY:**	**Treatment included in emergency kit?** YES_____NO_____
Reaction:	
Treatment:	

Action Items!

If you answered no to any of the questions above, take action to make sure you have the items needed. List your action items here:

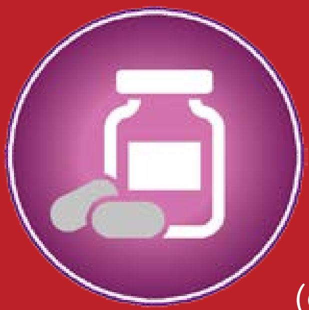

Medication

Medications fall into many categories: prescription and over-the-counter; those that are actively treating a condition, (e.g., antibiotics), those that maintain a condition (e.g., blood pressure medication) and those taken periodically in response to symptoms (e.g., Tylenol or ibuprofen for a headache). You need to know which medications are important for your health and if there are any restrictions that would make replacing your prescriptions more difficult in case of an emergency. If you're not sure, talk to your physician and or pharmacist.

What medicines do you take?

List medications in this section that are Extremely Important, Important or Moderately Important using the following definitions:

- Extremely Important — vital for life.
- Important — needed to manage serious health conditions.
- Moderately Important — needed to manage moderate health conditions that are not life threatening.

Prescription Medications Taken Regularly

Please list any prescription medications you take on a regular basis in the following tables.

**Medication name:**		
**Dosage:**	**How many times per day?**	**Specific instructions:**
**Prescriber:**	**Prescriber's phone number:**	**Three-day supply included in emergency kit?** YES______NO______
**Pharmacy name:**	**Address:**	**Phone:**
**Can you get replacement or temporary medication for this prescription from this location or a related pharmacy, during or after an emergency?**		YES______NO______
**IMPORTANCE OF MEDICATION:** *(circle one)*		
Extremely Important	*Important*	*Moderately Important*

**MEDICATION:**		
**Dosage:**	**How many times per day?**	**Specific instructions:**
**Prescriber:**	**Prescriber's phone number:**	**Three-day supply included in emergency kit?** YES_____NO_____
**Pharmacy name:**	**Address:**	**Phone:**
**Can you get replacement or temporary medication for this prescription from this location or a related pharmacy, during or after an emergency?**		YES_____NO_____
**IMPORTANCE OF MEDICATION:** *(circle one)*		
Extremely Important	*Important*	*Moderately Important*

**MEDICATION:**		
**Dosage:**	**How many times per day?**	**Specific instructions:**
**Prescriber:**	**Prescriber's phone number:**	**Three-day supply included in emergency kit?** YES_____NO_____
**Pharmacy name:**	**Address:**	**Phone:**
**Can you get replacement or temporary medication for this prescription from this location or a related pharmacy, during or after an emergency?**		YES_____NO_____
**IMPORTANCE OF MEDICATION:** *(circle one)*		
Extremely Important	*Important*	*Moderately Important*

**Medication name:**		
**Dosage:**	**How many times per day?**	**Specific instructions:**
**Prescriber:**	**Prescriber's phone number:**	**Three-day supply included in emergency kit?** YES_____NO_____
**Pharmacy name:**	**Address:**	**Phone:**
**Can you get replacement or temporary medication for this prescription from this location or a related pharmacy, during or after an emergency?**		YES_____NO_____
**IMPORTANCE OF MEDICATION:** *(circle one)*		
Extremely Important	*Important*	*Moderately Important*

Prescription Medications Taken Only When Needed

Please list any prescription medications you take only when needed.

MEDICATION:		
Dosage:	How many times per day?	Specific instructions:
Prescriber:	Prescriber's phone number:	Three-day supply included in emergency kit? YES_____NO_____
Pharmacy name:	Address:	Phone:
Can you get replacement or temporary medication for this prescription from this location or a related pharmacy, during or after an emergency?		YES_____NO_____
IMPORTANCE OF MEDICATION: *(circle one)*		
Extremely Important	*Important*	*Moderately Important*

MEDICATION:		
Dosage:	How many times per day?	Specific instructions:
Prescriber:	Prescriber's phone number:	Three-day supply included in emergency kit? YES_____NO_____
Pharmacy name:	Address:	Phone:
Can you get replacement or temporary medication for this prescription from this location or a related pharmacy, during or after an emergency?		YES_____NO_____
IMPORTANCE OF MEDICATION: *(circle one)*		
Extremely Important	*Important*	*Moderately Important*

MEDICATION:		
Dosage:	How many times per day?	Specific instructions:
Prescriber:	Prescriber's phone number:	Three-day supply included in emergency kit? YES_____NO_____
Pharmacy name:	Address:	Phone:
Can you get replacement or temporary medication for this prescription from this location or a related pharmacy, during or after an emergency?		YES_____NO_____
IMPORTANCE OF MEDICATION: *(circle one)*		
Extremely Important	*Important*	*Moderately Important*

Over-the-Counter Medications Taken Regularly

Please list any over-the-counter medications you take on a regular basis.

**MEDICATION:**		
**Dosage:**	**How many times per day?**	**Specific instructions:**
**IMPORTANCE OF MEDICATION:** *(circle one)*		
Extremely Important	*Important*	*Moderately Important*

**MEDICATION:**		
**Dosage:**	**How many times per day?**	**Specific instructions:**
**IMPORTANCE OF MEDICATION:** *(circle one)*		
Extremely Important	*Important*	*Moderately Important*

**MEDICATION:**		
**Dosage:**	**How many times per day?**	**Specific instructions:**
**IMPORTANCE OF MEDICATION:** *(circle one)*		
Extremely Important	*Important*	*Moderately Important*

Over-the Counter Medications Taken Only When Needed

Please list any over-the-counter medications you take only when needed.

**MEDICATION:**		
**Dosage:**	**How many times per day?**	**Specific instructions:**
**IMPORTANCE OF MEDICATION:** *(circle one)*		
Extremely Important	*Important*	*Moderately Important*

**MEDICATION:**		
**Dosage:**	**How many times per day?**	**Specific instructions:**
**IMPORTANCE OF MEDICATION:** *(circle one)*		
Extremely Important	*Important*	*Moderately Important*

Do you have any allergies to medications?

YES_____NO_____

If so, please list them below.

**MEDICINE ALLERGY:**	**Treatment included in emergency kit?**
Reaction:	YES_____NO_____
Treatment:	
**MEDICINE ALLERGY:**	**Treatment included in emergency kit?**
Reaction:	YES_____NO_____
Treatment:	
**MEDICINE ALLERGY:**	**Treatment included in emergency kit?**
Reaction:	YES_____NO_____
Treatment:	
**MEDICINE ALLERGY:**	**Treatment included in emergency kit?**
Reaction:	YES_____NO_____
Treatment:	
**MEDICINE ALLERGY:**	**Treatment included in emergency kit?**
Reaction:	YES_____NO_____
Treatment:	
**MEDICINE ALLERGY:**	**Treatment included in emergency kit?**
Reaction:	YES_____NO_____
Treatment:	
**MEDICINE ALLERGY:**	**Treatment included in emergency kit?**
Reaction:	YES_____NO_____
Treatment:	

Action Items!

If you answered no to any of the questions above, take action to make sure you have the items needed. List your action items here:

Pets

Pets are a very important part of our lives. We are responsible for their well-being and need to prepare for their needs during and after a disaster. Items such as food, bedding, carriers or cages, leashes, harnesses and collars should all be included in your emergency kit.

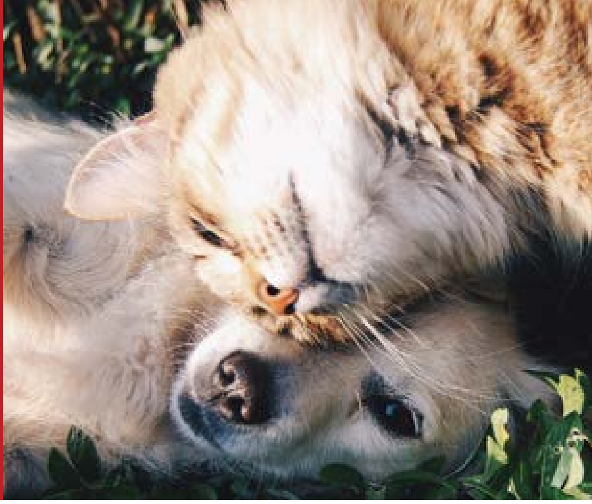

Don’t forget your furry friends!

Just like humans, some pets require specific food, medication and other items not readily available at general retail stores.

Have you included supplies for your pets in your emergency kit, such as food, toys, harnesses, leashes and tethers, etc.? YES______NO______

**PET #1**	**Source:** (Name and Phone number)
Name and Kind:	
Specific Need:	
**PET #2**	**Source:** (Name and Phone number)
Name and Kind:	
Specific Need:	
**PET #3**	**Source:** (Name and Phone number)
Name and Kind:	
Specific Need:	

Action Items!

If you answered no to any of the questions above, take action to make sure you have the items needed. List your action items here:

Service Animals

A service animal is a guide dog, signal dog or other animal trained to provide assistance to an individual with a disability. It's important to include service animals in your emergency planning.

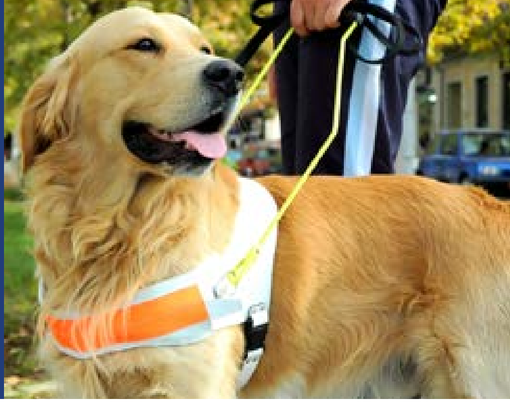

Do you have a service animal you depend on to provide assistance? YES_____NO_____?

Have you included copies of up-to-date veterinary documents and vaccination records related to your service animal in your emergency kit? YES______NO_____

Have you included supplies for your service animal in your emergency kit, such as food, harnesses and leashes and tethers, etc.? YES_____NO_____

**VETERINARIAN CONTACT INFORMATION:**
Name:
Address:
Phone:
Email:

Action Items!

If you answered no to any of the questions above, take action to make sure you have the items needed. List your action items here:

____

____

____

____

Learn more

For more information on emergency preparedness steps for service animals and pets in general, go to the following websites:

PrepareMetroKC	preparemetrokc.org/Be_Prepared/pets.asp
American Red Cross	redcross.org/images/MEDIA_CustomProductCatalog/m3640126_PetSafety.pdf<br>redcross.org/prepare/location/home-family/pets

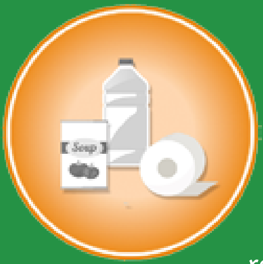

Supplies

Supplies that get used regularly or wear out make up a significant portion of an emergency kit. Do not assume items you need will be provided by emergency responders, relief workers and shelter providers.

Do you need specific supplies?

Supplies can be divided into three categories, all of which should be included in your emergency kit:

1. Standard supplies, such as food, water, clothes, first aid items, basic toiletries and sanitation supplies, including toilet paper, baby wipes and hand sanitizer, etc. (These are supplies that everyone needs to have in their kit and do not need to be listed in this section).

2. Additional supplies specific to your needs that can be readily replaced at a variety of retail outlets, such as hearing aid batteries and adult disposable briefs.

3. Additional supplies specific to your needs that are only sold by particular outlets or sources, such as medical oxygen tanks or ostomy supplies.

List the supplies that are important for your daily functioning and those that are hard to find.

1. Supply item: ____

   a. Where can you find the item? ______________________________

   b. Address: ____

   c. Phone: ____

   d. If you get this item from a specific supplier or vendor, do they have a plan for providing services during and after an emergency? YES_____NO_____

   e. Alternate source for the supply item:___________________________

   f. Address:____

   g. Phone: ______________________________________

   h. Will the supplier provide services during an emergency? YES_____NO_____

   i. How often do you use this item?____________________________

   j. Do you have a three-day supply of this item in your kit? YES_____NO_____

2. Supply item: ____

   a. Where can you find the item? ______________________________

   b. Address: ____

   c. Phone: ____

   d. If you get this item from a specific supplier and vendor, do they have a plan for providing services during and after an emergency? YES_____NO_____

   e. Alternate source for the supply item:___________________________

   f. Address:____

   g. Phone: ____

   h. Will the supplier provide services during an emergency? YES_____NO_____

   i. How often do you use this item? ____________________________

   j. Do you have a three-day supply of this item in your kit? YES_____NO_____

3. Supply item: ____

   a. Where can you find the item? ______________________________

   b. Address: ____

   c. Phone: ____

   d. If you get this item from a specific supplier and vendor, do they have a plan for providing services during and after an emergency? YES_____NO_____

   e. Alternate source for the supply item:___________________________

   f. Address:____

   g. Phone: ____

   h. Will the supplier provide services during an emergency? YES_____NO_____

   i. How often do you use this item? ____________________________

   j. Do you have a three-day supply of this item in your kit? YES_____NO_____

Action Items!

If you answered no to any of the questions on the previous page, take action to make sure you have the items needed. List your action items here:

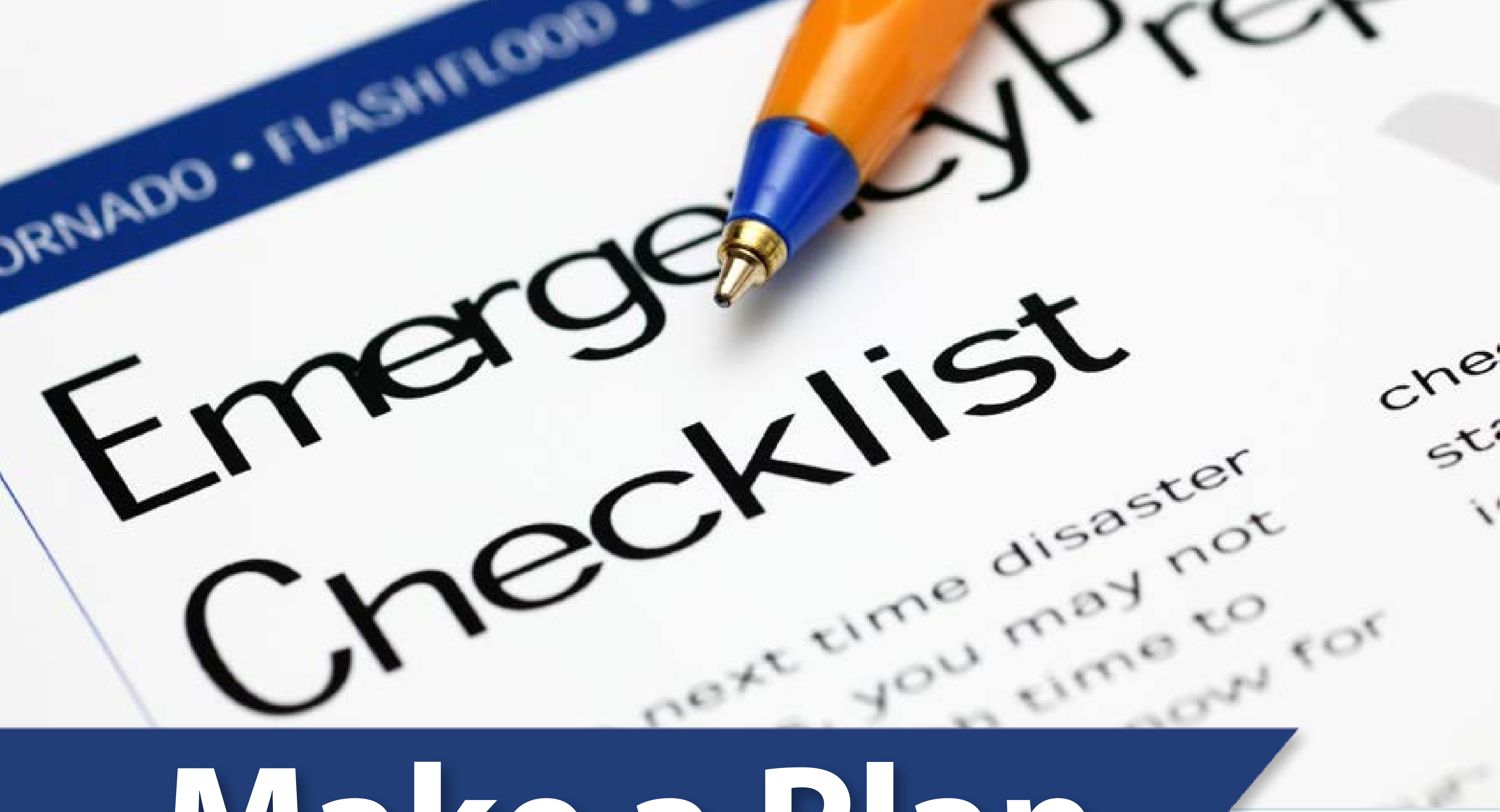

Make a Plan

Action Item!

If you haven't already put together your standard emergency plan, please start immediately. For resources on making a plan, go to the following websites:

PrepareMetroKC	preparemetrokc.org/memc-brochure.pdf<br>preparemetrokc.org/be_prepared/plan.asp
Federal Emergency Management Agency	http://goo.gl/zKZZZ3
American Red Cross	redcross.org/prepare/location/home-family/plan
Centers for Disease Control and Prevention	emergency.cdc.gov/preparedness/index.asp

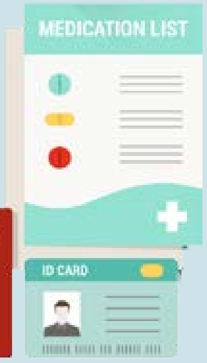

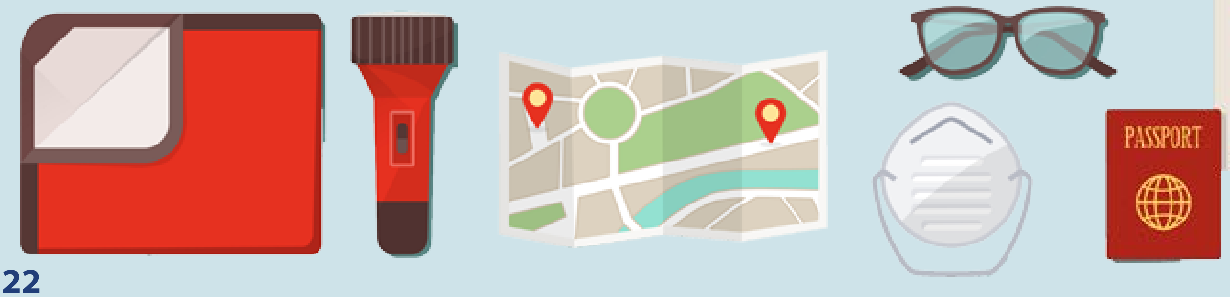

Lodging/Shelter

Different types of disasters require different approaches to lodging or sheltering, but seeking safe shelter is a priority in emergency situations. Having multiple options for obtaining immediate shelter and for long-term lodging allows you to be prepared should a disaster strike.

Unsafe to stay? Where will you go?

If you have to evacuate your home during an emergency, you will need to find a safe place to go and decide how to get there. You may choose to go to a hotel or motel, stay with friends or relatives, or find an evacuation shelter.

1. If you need to leave your home suddenly due to a disaster, such as a fire or flash flooding, do you have an escape plan for your residence? YES_____NO_____

2. If you usually take an elevator to get to a level where you can exit the building, do you have an alternate way to get to that level? YES_____NO_____

3. Where are the potential meeting places (within your neighborhood and outside your neighborhood) that you, your family, neighbors and friends have agreed upon in case of evacuation?

   Locations near residence:____
   Locations outside your neighborhood:____________________________________

4. If you must seek shelter safely outside the affected area before or after a predicted hazard strikes, such as a snow or ice storm, where would you go?

   Name of place or contact: ____
   Address:____
   Phone:____

   Can this location accommodate your daily essential needs and supply important equipment to help you function? YES_____NO_____

   Name of place or contact: ____
   Address:____
   Phone:____

   Can this location accommodate your daily essential needs? YES_____NO_____

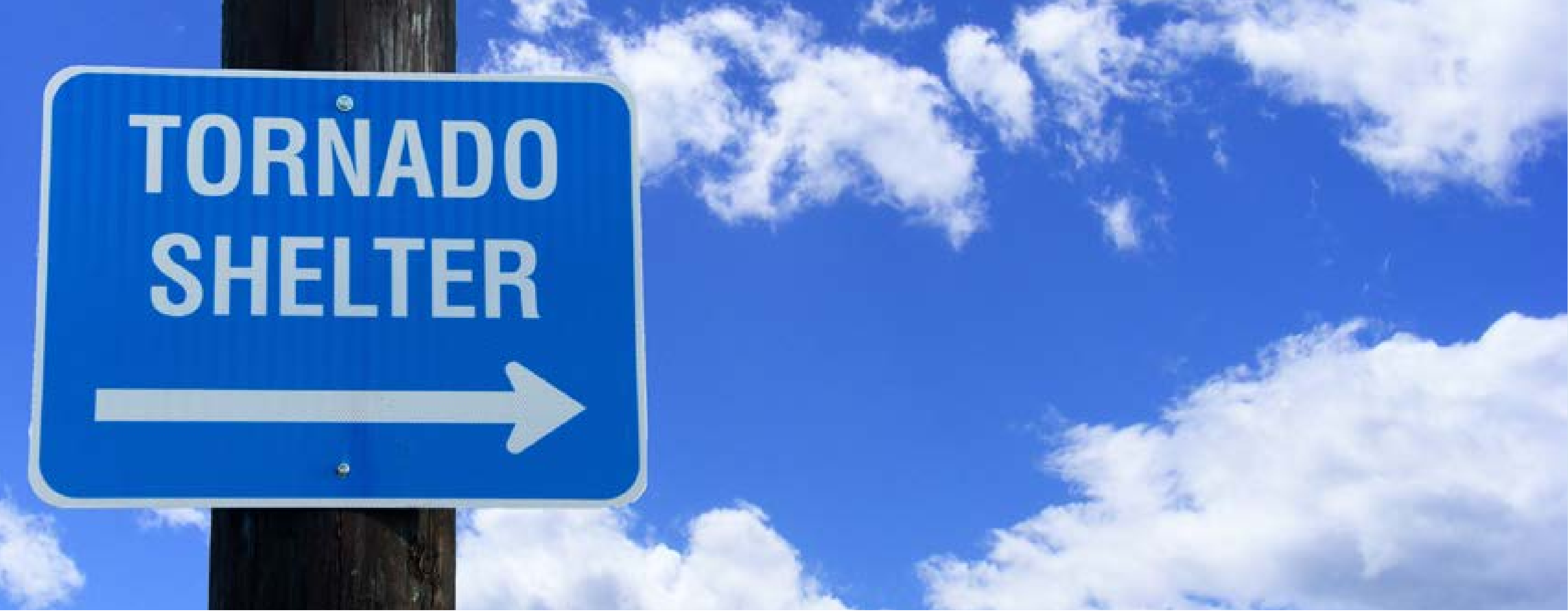

Sheltering for severe weather

In the case of severe thunderstorms and tornadoes, the safest place to be is inside an interior room without windows on the lowest level of the structure you are in. However, in many multi-level residences, the basement or the lower level is only accessible by elevator or stairs. It is not advisable to take an elevator to the lowest level. You will need an alternate way to get to safe shelter or find another safe place to go.

1. Does your residence have a basement or a lower level where you can go for safety?
   YES_____NO_____

2. If you have mobility issues and if the basement or lower level of your residence is only accessible by elevator and/or stairs, do you have an alternate way to get to that level?
   YES_____NO_____

3. If you have mobility issues and you cannot get to a basement or lower level, does your residence have a small, interior room without windows, such as a closet or bathroom, where you could seek shelter if necessary? YES_____NO_____

Sheltering in place

"Shelter-in-place" means to take shelter where you are at home, work or school, and to seal the room you are sheltering in as thoroughly as possible. This action may be needed when the air quality has become unhealthy due to the release of chemical, biological or radiological substances.

1. Does your residence or building have an interior room without windows, such as a closet or bathroom, that you can access for safe shelter? YES_____NO_____

2. Are you able to turn your central heat and air system off? YES_____NO_____

Additional Resources

For more information on escape and evacuation plans, go to:

PrepareMetroKC	preparemetrokc.org/Be_Prepared/homeescape.asp<br>preparemetrokc.org/Be_Prepared/evacuation.asp
American Red Cross	redcross.org/prepare/location/home-family/plan

For more information regarding safe lodging, check out the following resources:

PrepareMetroKC	preparemetrokc.org/Be_Prepared/shelter-in-place.asp
American Red Cross	redcross.org/images/MEDIA_CustomProductCatalog/<br>m4340182_shelterinplace.pdf
Federal Emergency Management Agency	ready.gov/shelter
Centers for Disease Control and Prevention	emergency.cdc.gov/preparedness/shelter/

Action Items!

If you answered no to any of the questions on the previous page, take action to make sure you have the items needed. List your action items here:

Medical Treatments

If you recieve medical treatments or procedures on a regular basis from a clinic, treatment center, doctor's office or hospital, it's important to have a backup option in the event a medical facility isn't accessible.

Do you receive regular treatments?

If so, list any medical procedures or treatments that you receive regularly here:

1. Medical treatment or procedure: ______________________________

   a. Importance of treatment or procedure:

      Extremely Important   Important   Moderately Important

   b. Where do you get the treatment or procedure?__ ______________________

      Location name: ______________________________
      Address: ______________________________
      Phone:______________________________

   c. Is the provider able to provide services after an emergency? YES_____NO_____

   d. In what ways will the provider try to communicate with you during or after an emergency event?______________________________

   e. Alternate source for the treatment or procedure: ______________________

      Location name: ______________________________
      Address: ______________________________
      Phone:______________________________

   f. Is the provider able to provide services during an emergency? YES_____NO_____

Action Items!

If you answered no to any of the questions above, list your action items here:

____

____

____

____

People

Throughout our lives there are many times we might need to rely on other people to help us. Whether we are very young or growing older, fighting a chronic illness or living with a disability, struggling to communicate or make ends meet, assistance from others can make a difference. During a disaster, it is important to have a support network of individuals you can contact for help.

Who do you depend on most?

The people that help us every day — caregivers, family members, friends, medical professionals, neighbors and co-workers are important resources. In a disaster situation, some of these people may be able to help you start recovering, while others may not be able to provide assistance.

Action Item!

Think about the people you depend upon for essential daily functions, such as getting dressed, preparing meals and transportation. Have conversations with these people about your and their emergency preparedness plans. Are they willing to be listed as a resource to call upon should a disaster take place? Are there others who are willing to be a "backup" in case your normal providers are not available?

Tip #1 Gather information for several people who would be willing to help you during an emergency.

Tip #2 For those people that provide services to you, such as case workers, personal care assistants and medical professionals, ask if they plan to offer service after a disaster if their location is not affected and modify their normal service to meet the needs of the situation.

Family, friends and others

Who are the local people who know you and your capabilities best? These could be family members, friends, roommates or coworkers you might call on for assistance. List at least three people with their contact information.

**NAME:**
Phone:
Email:
**NAME:**
Phone:
Email:
**NAME:**
Phone:
Email:
**NAME:**
Phone:
Email:
**NAME:**
Phone:
Email:

Who are other people you know who you could call for assistance, such as your landlord, neighbor or driver? List at least three people with their contact information.

**NAME:**
Relationship:
Phone:
Email:
**NAME:**
Relationship:
Phone:
Email:
**NAME:**
Relationship:
Phone:
Email:

Physicians

If you have a medical condition that a physician regulary monitors, talk to your doctor about his or her emergency plans. Work with your doctor to identify backup service providers within your area and any place you might evacuate to.

Are you regularly seeing a doctor?

Do you have a primary care physician who actively monitors your condition? YES_____NO_____

1. Physician and practice name: ____

   a. Phone:____

   b. Website: ____

   c. Does this provider have a plan for providing services during emergency events? YES_____NO_____

   d. In what ways will this physician's office try to communicate with you during an emergency?

   ____

Are there other medical providers that provide care for you? YES_____NO_____

1. Physician and practice name: ____

   a. Type of service provided: ____

   b. Phone:____

   c. Website: ____

   d. Does this provider have a plan for providing services during emergency events? YES_____NO_____

   e. In what ways will this physician's office try to communicate with you during an emergency?

   ____

Physicians, continued

2. Physician and practice name: ______________________________

    a. Type of service provided: ______________________________

    b. Phone:______________________________

    c. Website: ______________________________

    d. Does this provider have a plan for providing services during emergency events? YES______NO______

    e. In what ways will this physician's office try to communicate with you during an emergency?

    ____

3. Physician and practice name: ______________________________

    a. Type of service provided: ______________________________

    b. Phone:______________________________

    c. Website: ______________________________

    d. Does this provider have a plan for providing services during emergency events? YES______NO______

    e. In what ways will this physician's office try to communicate with you during an emergency?

    ____

Action Items!

If you answered no to any of the questions above, list your action items here:

____

____

____

____

____

____

Translators/Interpreters

Translators and interpreters who are physically present during a crisis help provide immediate services, such as medical interpreting and enabling communication between rescue teams, technically-skilled professionals and disaster assistance organization personnel.

Does someone help you communicate?

Do you have a translator or language interpreter who helps you? YES_____NO_____

1. Translator/interpreter name: ____

   a. Type of service provided: ____

   b. Phone:____

   c. Website: ____

   d. Does your translator/interpreter have a plan for providing services during emergency events? YES_____NO_____

   e. In what ways will the translator/interpreter try to communicate with you during an emergency?____

   f. Is an additional contract needed for services provided after a disaster? YES_____NO_____

   g. If an additional contract is necessary, have you and the translator/interpreter signed that type of agreement? YES_____NO_____

2. Name of additional translator/interpreter: ____

   a. Type of service provided: ____

   b. Phone:____

   c. Website: ____

   d. Does this translator/interpreter have a plan for providing services during emergency events? YES_____NO_____

   e. In what ways will the translator/interpreter try to communicate with you during an emergency?____

   f. Is an additional contract needed for services provided after a disaster? YES_____NO_____

   g. If an additional contract is necessary, have you and the translator/interpreter signed that type of agreement? YES_____NO_____

Action Items!

If you answered no to any of the questions above, list your action items here:

____

____

____

____

Case manager/ service coordinator

Case managers and service coordinators provide helpful information and access to services, resources and benefits. After a disaster, they can help get you on the road to recovery.

1. Case manager/service coordinator's name: ______________________________

    a. Phone:______________________________

    b. Email: ______________________________

    c. Does this case manager/service coordinator have a plan for providing services during emergency events? YES_____NO_____

    d. Does this case manager/service coordinator have a plan for emergency events? YES_____NO_____

    e. In what ways will this person try to communicate with you during a disaster?

    ____

Action Items!

If you answered no to any of the questions above, list your action items here:

____

____

____

____

In-home caregivers

If you cannot perform certain daily functions on your own, emergency shelters will likely require you to have a caregiver or personal care attendant with you in the shelter. Do you have a caregiver or personal attendant who provides services in your home regularly?

Do you regularly need help with personal care?

If the answer is yes, please list all the people who qualify as caregivers or personal attendants that provide essential services for daily functions. Use the questions below when you discuss preparedness planning efforts.

NOTE: If you cannot perform certain daily functions on your own, emergency shelters will likely require you to have a caregiver/personal care attendant with you in the shelter.

1. Caregiver's name: ____

   a. Type of service provided: ______________________________________

   b. Phone:____

   c. Email: ____

   d. Does this caregiver have a plan for providing services during emergency events?
   YES______NO______

   e. In what ways will this caregiver try to communicate with you during an emergency?
   ____

   f. Is this caregiver available to provide services during and after a disaster in a location other than your home, such as a shelter? YES______NO______

   g. Is this caregiver able to transport you to a location other than your home?
   YES______NO______

   h. Is an additional contract needed for services provided after a disaster?
   YES______NO______

   i. If an additional contract is necessary, have you and your caregiver signed that type agreement? YES______NO______

Caregivers, continued

2. Caregiver's name: ____________________

   a. Type of service provided: ____________________

   b. Phone: ____________________

   c. Email: ____________________

   d. Does this caregiver have a plan for providing services during emergency events? YES_____NO_____

   e. In what ways will this caregiver try to communicate with you during an emergency? ____________________

   f. Is this caregiver available to provide services during and after a disaster in a location other than your home, such as a shelter? YES_____NO_____

   g. Is this caregiver able to transport you to a location other than your home? YES_____NO_____

   h. Is an additional contract needed for services provided after a disaster? YES_____NO_____

   i. If an additional contract is necessary, have you and your caregiver signed that type agreement? YES_____NO_____

3. Caregiver's name: ____________________

   a. Type of service provided: ____________________

   b. Phone: ____________________

   c. Email: ____________________

   d. Does this caregiver have a plan for providing services during emergency events? YES_____NO_____

   e. In what ways will this caregiver try to communicate with you during an emergency? ____________________

   f. Is this caregiver available to provide services during and after a disaster in a location other than your home, such as a shelter? YES_____NO_____

   g. Is this caregiver able to transport you to a location other than your home? YES_____NO_____

   h. Is an additional contract needed for services provided after a disaster? YES_____NO_____

   i. If an additional contract is necessary, have you and your caregiver signed that type agreement? YES_____NO_____

4. Caregiver's name: ____

a. Type of service provided: ______________________________________

b. Phone: ____

c. Email: ____

d. Does this caregiver have a plan for providing services during emergency events? YES_____NO_____

e. In what ways will this caregiver try to communicate with you during an emergency? ____

f. Is this caregiver available to provide services during and after a disaster in a location other than your home, such as a shelter? YES_____NO_____

g. Is this caregiver able to transport you to a location other than your home? YES_____NO_____

h. Is an additional contract needed for services provided after a disaster? YES_____NO_____

i. If an additional contract is necessary, have you and your caregiver signed that type agreement? YES_____NO_____

Action Items!

If you answered no to any of the questions above, list your action items here:

____

____

____

____

____

____

____

____

____

____

____

____

Pets

For many people, pets are an important member of the family. The likelihood that you and your pets will survive an emergency, such as a fire, flood or tornado, depends largely on emergency planning done today.

Who will care for your pets?

Whether you decide to stay put in an emergency or evacuate to a safer location, you need to plan ahead for your pets. If you must evacuate, take your pets with you if possible. It's important to know that animals may not be allowed inside public shelters, so plan in advance for shelter alternatives that will work for both you and your pets.

Do you have pets? YES_____ NO_____ If so, what type?________________________________

If you have pets, do you have friends, family or neighbors who are willing to take care of them after a disaster if you are unable to take them with you? YES_____ NO_____

Name: ____

Phone:____

Name: ____

Phone:____

Name: ____

Phone:____

List veterinarian contact information:

Name: ____

Address:______________________________________

Phone:____

Email: ____

Will your veterinarian provide shelter for your pets during or after an emergency if they are not affected by the disaster? YES_____ NO_____

List boarding facility contact information:

Name: ____________________

Address: ____________________

Phone: ____________________

Email: ____________________

Will your boarding facility provide shelter for your pets during or after an emergency if they are not affected by the disaster? YES______ NO______

List grooming facility contact information:

Name: ____________________

Address: ____________________

Phone: ____________________

Email: ____________________

Will your grooming facility provide shelter for your pets during or after an emergency if they are not affected by the disaster? YES______ NO______

Action Items!

If you answered no to any of the questions above, list your action items here:

Transportation

Transportation can be a critical resource when it comes to disasters. Some disasters provide enough warning time that the best option may be to evacuate before the disaster hits. Other emergencies happen with little notice and you may not be able to go anywhere until after the situation has stabilized.

How will you get around after a disaster?

In any disaster situation, please consider what your safest options are when seeking transportation services. Do you rely on a transportation service provider for your regularly scheduled transportation or periodic transportation needs? It is good to have several options for transportation in case your normal source is affected by the disaster.

Please list transportation providers who can accommodate your particular needs.

1. Transportation provider's name:________ ______________________________________
   a. Phone:____
   b. Email: ____
   c. Does this transportation provider have a plan for providing services during emergency events? YES_____NO_____
   d. In what ways will this provider try to communicate with you during a disaster?

      ____

2. Alternate transportation option:______________________________________
   a. Phone:____
   b. Email: ____
   c. Does this transportation provider have a plan for providing services during emergency events? YES_____NO_____
   d. In what ways will this provider try to communicate with you during a disaster?

      ____

3. Alternate transportation option:________ ____

a. Phone:____

b. Email: ____

c. Does this transportation provider have a plan for providing services during emergency events? YES_____NO_____

d. In what ways will this provider try to communicate with you during a disaster?

____

Action Items!

If you answered no to any of the questions above, list your action items here:

____

____

____

____

____

____

____

____

____

____

____

____

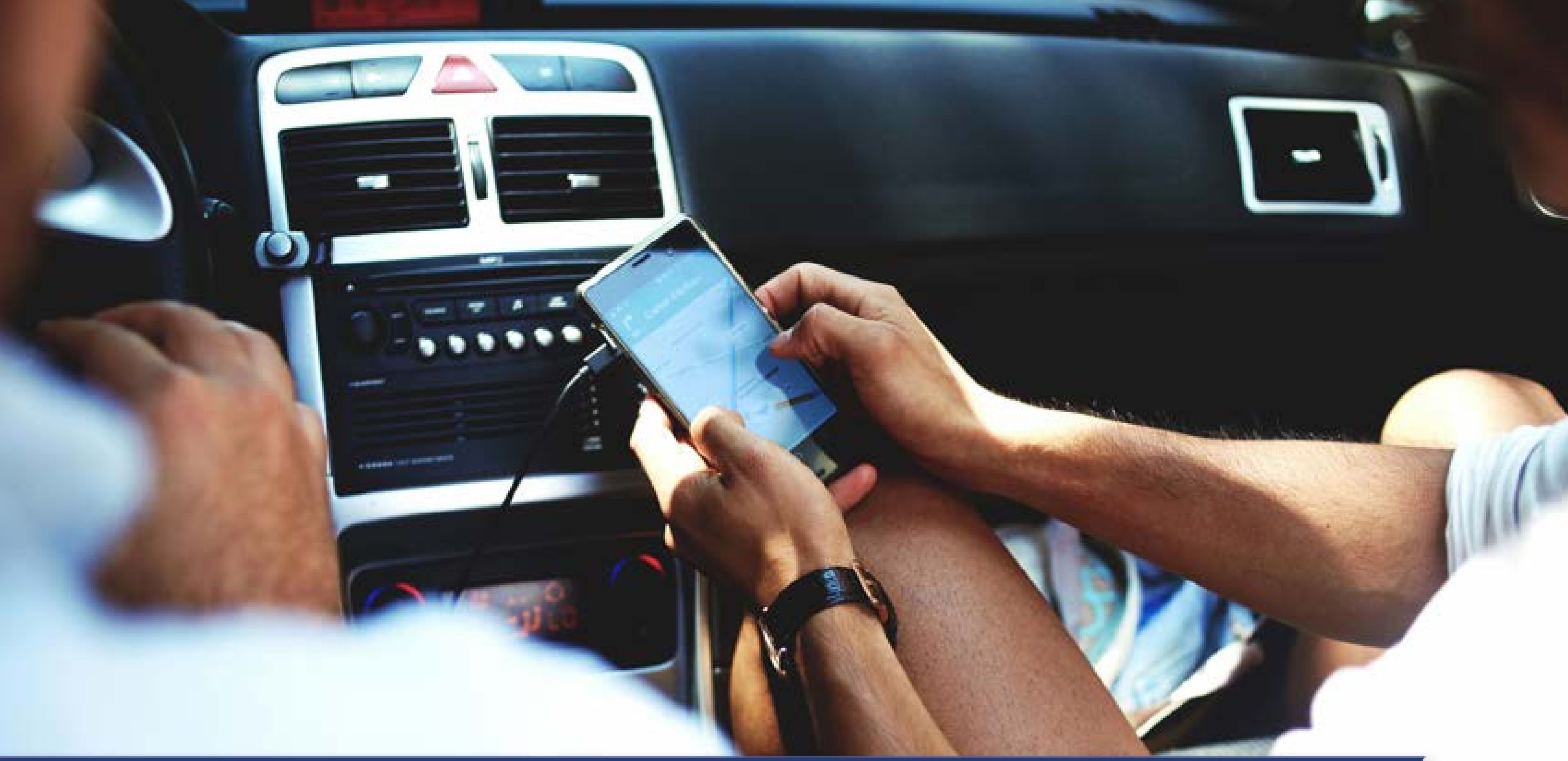

Stay Informed

Action Item!

Knowing what to do before, during and after an emergency is a critical part of being prepared and may make all the difference when seconds count. Take the time now to identify how local authorities and news organizations will notify you during a disaster. For additional resources on staying informed, go to the following websites:

PrepareMetroKC	preparemetrokc.org/social.asp
National Oceanic and Atmospheric Administration Weather Radio All Hazards	nws.noaa.gov/nwr
Federal Emergency Management Agency	fema.gov/mobile-app
American Red Cross	redcross.org/get-help/prepare-for-emergencies/mobile-apps

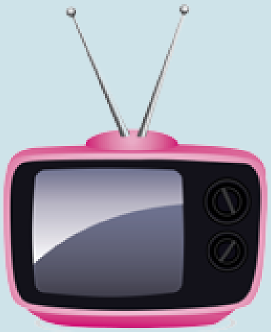

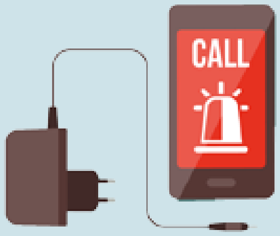

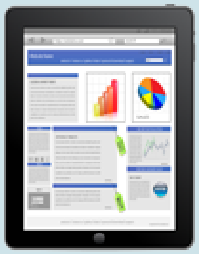

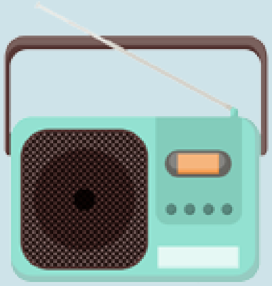

Communications

Before, during and after an emergency, it is important to follow instructions from local officials and news organizations. This information can help you take appropriate action and keep you safe. It's also a good idea to have multiple ways to send and receive information in case one of your regular communication devices does not function.

How will you stay informed during a disaster?

In order to prepare for any type of emergency, you must be informed on the latest news. Watch television, listen to the radio and check websites for instructions from local and state officials.

Which of the following communication tools do you have? Check all that apply and answer the questions that follow.

☐ Cell Phone

1. Are you able to download apps on your phone? YES_____NO_____

2. If yes, have you downloaded any weather alert or emergency preparedness apps, such as the Wireless Emergency Alerts app and the Red Cross Emergency app? YES_____NO_____

3. If you need assistance communicating with others and have the ability to download apps to your phone, have you downloaded any assistive communication apps? YES_____NO_____

☐ Landline Phone

1. Does your residence have a landline telephone? (Hardwired landlines that are not cordless may not need electricity or batteries.) YES_____NO_____

2. Does your phone or handset have adaptation features that help you use the device? YES_____NO_____

3. If you have a cordless landline phone, do you have a backup method to charge the phone or a different type of phone in your emergency kit? YES_____NO_____

Communications, continued

☐ Television

1. If you have a hearing impairment, do you have closed-captioning capabilities for your television to stay informed of hazards that may be imminent?
   YES_____NO_____

2. Do you have other equipment that connects with your television to help you understand the content coming from the television better? YES_____NO_____

3. If yes, and if the equipment is portable, have you included it in your emergency plans?
   YES_____NO_____

☐ Mass Notification Systems

1. Does your community have a mass notification system which allows you to get updates about urgent situations? YES_____NO_____

2. Have you signed up to receive notifications from that system? YES_____NO_____

☐ Video Phone, Video Relay Service or Telecommunications Relay Service

1. Do you use a video phone, video relay service or telecommunications relay service to communicate with others? YES_____NO_____

2. Have you explored ways that these devices/systems can be portable if you have to leave your home due to a disaster? YES_____NO_____

☐ Medical Alert System

1. Do you use a medical alert system, such as Life Alert, LifeStation, Medical Alert, MobileHelp and Philips Lifeline, that you can contact in an emergency or one that will check on you in certain circumstances? YES_____NO_____
   a. If yes, list the service provider: ____
   b. Phone:____

2. Does your medical alert system have a battery backup? YES_____NO____
   a. If yes, is it in your emergency kit or readily accessible? YES_____NO____

☐ Augmentative and Alternative Communication (AAC) System

1. Do you have a type of AAC tool or device, such as electronic tablet or message board? YES_____NO_____

2. If the system is reliant on a battery, do you have a backup battery or another way to power the device? YES_____NO_____

☐ Other Portable Electronic Devices

1. If you have a hearing or speech impairment, do you have a portable device that is specifically meant to help you receive information or communicate with others? Examples include assistive listening devices or one-to-one communication devices, such as UbiDuo? YES_____NO_____

2. If so, have you included the portable devices in your emergency plans, so you can take them with you if you have to leave your home? YES_____NO_____

Life-saving devices

It is also important to have life-saving devices, such as smoke and carbon monoxide detectors, that will alert you to dangerous situations. Which devices do you have? Check all that apply.

☐ Smoke Alarms

1. Do you have smoke alarms in your home? YES_____NO_____
   - a. If so, are your smoke alarms working? YES_____NO_____
   - b. Have you changed your smoke alarm batteries in the past six months? YES______NO______
   - c. Do you have extra batteries for your smoke alarms? YES_____NO_____
   - d. If you have a hearing impairment, do you have a signaler on your smoke alarm that helps ensure you are aware of the presence of smoke or fire? YES_____NO_____

☐ Carbon Monoxide Detectors

1. Do you have carbon monoxide detectors in your home? YES_____NO_____
   - a. If so, are the carbon monoxide detectors working? YES_____NO_____
   - c. If your carbon monoxide detectors are battery powered, have you changed the batteries in the past six months? YES_____NO_____
   - d. Do you have extra batteries for your carbon monoxide detector? YES_____NO_____

Action Items!

If you answered no to any of the questions above, list your action items here:

Sponsors

Community Disaster Resiliency Network

MARC
MID-AMERICA REGIONAL COUNCIL

Margaret A. Cargill
FOUNDATION
A MARGARET A. CARGILL PHILANTHROPY

Contact:

Jennifer Wyatt, The Whole Person,
jenniferwyatt.lac@gmail.com

Mary Carter, Mid-America Alliance for Access,
marycatcar@gmail.com

Jake Jacobs, Developmental Disability Services of Jackson County - eitas,
jjacobs@eitas.org, 816-363-2000, ext. 207

Donna Martin, Mid-America Regional Council,
dmartin@marc.org, 816-701-8369
