# Supplementary material for: Disaster Preparedness Intervention for Older Adults (Seniors’ Positive Involvement in Community Emergencies): Protocol for a Quasi-Experimental Study
Source: JMIR Res Protoc. 2024 Dec 4;13:e58895. doi: 10.2196/58895 (PMC11656111; doi:10.2196/58895)
Supplement: Multimedia Appendix 2 [file resprot_v13i1e58895_app2.pdf]

MEMC

Metropolitan Emergency Managers Committee

Your guide to

EMERGENCY PREPAREDNESS

in the Greater Kansas City region

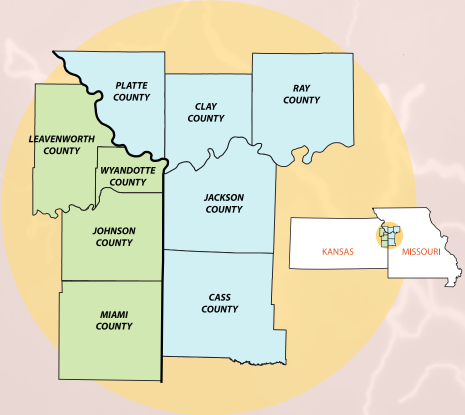

Published in cooperation with the Regional Homeland Security Coordinating Committee

MID-AMERICA REGIONAL COUNCIL

HOMELAND SECURITY

Preparedness Starts With You!

Disasters and emergencies can happen anytime and anywhere.

Whether it's a pandemic, hazardous materials spill or a tornado, the time to prepare is now. Different disasters require different preparations. A spill of hazardous material could mean immediate evacuation. A winter storm could confine your family at home. An earthquake, tornado or any other disaster could cut off basic services — gas, water, electricity and telephone — for days.

After a disaster, local officials and relief workers will not be able to reach everyone immediately. Help could come in hours, or it may take days. Will your family be ready?

You'll cope best by preparing for disaster before it strikes. In this booklet, we offer simple guidelines that will help you and your family prepare for emergencies.

The time to prepare is now — before disaster strikes.

Table of Contents

Know the Risks

Calling 911 2
NOAA All-Hazards Weather Radios 3
Tornadoes 4
Severe Thunderstorms 5
Flash Floods 6
Earthquakes 7
Excessive Heat 8
Drought 9
Winter Weather 10
Food Safety/Power Outages 11
Portable Generator Safety 12
Carbon Monoxide Dangers 13
Fire 14
HazMat/Shelter in Place 15
Terrorism/Active Shooter 16
Transportation Safety 17
Pandemic Flu/Infectious Disease 18

Get Prepared

First Aid 19
Make an Emergency Plan 20
Create a Disaster Supply Kit 22
Older Adults 24
Children and their Trusted Adults 25
Access and Functional Needs 26
Pet Safety 27
Web Resources 28
Get Involved 29
Your Evacuation Plan 30
Your Utility Information 31
Home Hazard Hunt 32
Family Medical Information 33
Important Numbers 34
Local Emergency Numbers 35
Emergency Management Offices 36

Calling 911

Call 911 to stop a crime, to report a fire, to save a life — or anytime an emergency response is required by law enforcement, fire or emergency personnel. You should call 911 anytime you believe there is an actual emergency. If you are unsure, call 911 and the dispatcher will make the final determination. When you call 911, the dispatcher will ask you five basic questions:

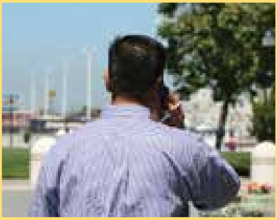

- Where is this happening?
- When did this happen?
- What is happening now? Why?
- Who is involved?
- Is anyone injured?

If you accidentally dial 911, stay on the line and explain to the dispatcher. Otherwise the dispatcher will have to call you back.

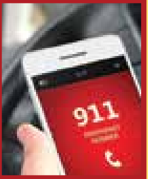

Call if you can, text if you can't!

Text to 911 service is now available in the Kansas City metro area. While calling 911 is still the best way to report an emergency, text to 911 is useful in situations where you cannot speak safely, and is ideal for people who are deaf, hard of hearing or have difficulty speaking.

NOAA All-Hazards Radios

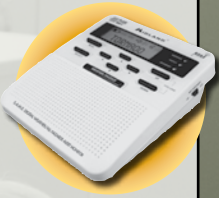

NOAA (National Oceanic and Atmospheric Administration) all-hazards weather radios provide constant, up-to-date weather information. During severe weather, the National Weather service broadcasts a special tone that activates weather radios in affected areas. You can program your radio to sound warnings for specific counties.

By sounding alarms inside homes, schools and businesses, all-hazards weather radios can alert people who are indoors — and save lives.

Project Community Alert is a community-wide effort to distribute all-hazards weather radios in the Greater Kansas City area. The Metropolitan Emergency Managers Committee partners with local Price Chopper grocery stores to sell the radios at a special price.

While these radios are primarily used for severe weather alerts, authorities can also use them to issue warnings about other types of emergencies, such as chemical spills or biological hazards.

For more information about Project Community Alert, visit **marc.org/ProjectCommunityAlert**.

For more information about NOAA weather radios, visit **www.weather.gov/nwr**.

Tornadoes

Tornadoes are capable of tremendous destruction. Be alert for watches and warnings in your area.

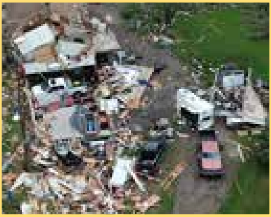

Tornado safety tips

- Take your emergency kit with you to the lowest level possible in a structure.
- Put as many walls between you and the outside as you can. Avoid windows and glass.
- In a basement, stay under a center support beam, a stairwell or heavy piece of furniture.
- If you have no basement, go to a small interior room (bathroom or closet) away from outside walls and windows.
- If a tornado is visible when driving, you may be able to drive out of its path by moving at right angles to the tornado. Otherwise, stay in your car with your seat belt on and head below window glass. Avoid seeking shelter under bridges or overpasses.
- Mobile homes offer no protection from tornadoes. Get to a safe shelter if possible.

Tornado Warning: A tornado has been sighted or indicated by weather radar.

Tornado Watch: Tornadoes are possible in your area. Remain alert. Find out what counties are in the watch area by listening to your NOAA all-hazards radio or local media.

Severe Thunderstorms

Not all thunderstorms are classified as severe, but all are dangerous. Thunderstorms may include strong winds, lightning, hail, heavy rain, flooding, downbursts and tornadoes. Although they are most likely in spring and summer, they can occur year round.

Thunderstorm safety tips

- If you can hear thunder, go indoors.
- Avoid using electrical appliances and stay off the phone except in emergencies.
- Do not take a bath or a shower during a thunderstorm.
- If you are caught outdoors, find a low spot away from trees, fences and poles.
- If you are boating or swimming, get to land and find shelter immediately!

The 30-30 rule:

If there's 30 seconds or less between a flash of lightning and the sound of thunder, seek shelter immediately.

Wait at least 30 minutes after the last clap of thunder before leaving shelter.

During a thunderstorm, each flash of cloud-to-ground lightning is a potential killer. If you feel your skin tingle or your hair stand on end, squat low on the balls of your feet, with your hands on your knees and your head between them.

Floods/Flash Floods

Flash floods can occur within minutes or hours of excessive rainfall. Whether you are driving or walking, if you come to a flooded road, the National Weather Service advises you to "Turn Around — Don't Drown." Even six inches of fast-moving floodwater can knock you off your feet, and a depth of two feet will float your car.

Flood Safety Tips

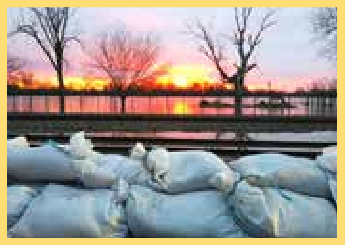

- Monitor your NOAA all-hazards weather radio or local television and radio stations for weather-related information.
- If flooding occurs, get to higher ground. Get out of areas that are normally subject to flooding.
- Avoid areas that are already flooded, especially if the water is flowing fast. Do not attempt to cross flowing streams.
- Remember, road beds may be washed out under flood waters. NEVER drive through flooded roadways.
- Do not park your vehicle near streams or creeks, particularly during threatening conditions.
- Take extra precautions at night when it is harder to recognize flood dangers.

Earthquakes

One of the largest earthquakes ever recorded in the U.S. took place in Missouri. Earthquakes are most common in the western states, but they can happen any time, anywhere.

Safety rules and precautions

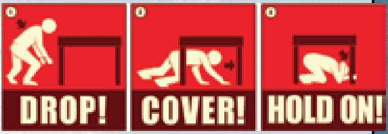

Image courtesy of Great ShakeOut Earthquake Drills, ShakeOut.org

- Before an earthquake, identify places in your house to take shelter, such as under a sturdy table or next to an inside wall.
- Don't place beds by windows, and don't hang heavy items over beds.
- Secure things that might fall, such as heavy TVs.
- Put strong latches on cupboards.
- During an earthquake, stay indoors. When you do leave a building, move away from it quickly.
- Get away from windows to avoid breaking glass.
- Don't use elevators.
- Wait until the shaking stops completely before venturing out.
- Outdoors, find a spot away from buildings, trees and power lines. Drop to the ground.
- If you are in a car, stop in a clear location as quickly as you can. Stay in the car with your seat belt fastened.
- Avoid the use of candles or open flame after an earthquake in case of gas leaks.

Excessive Heat

Many people do not realize how deadly a heat wave can be. More people die in an average year in Kansas City from heat-related conditions than from all other weather types combined.

Heat Safety Tips

- Do not leave children or pets in a vehicle. Temperatures can reach over 140° within minutes.
- Drink plenty of water, even if you're not thirsty. Avoid alcohol and caffeine.
- Wear loose-fitting, lightweight, light-colored clothing.
- Avoid going out during the hottest times of the day. If you must go out, use sunscreen and wear a wide-brimmed hat.
- Inside during the day, keep shades drawn and blinds closed. Use air conditioning when available.
- Fans should not be your primary source of cooling. Blow hot air out a window with a fan during the day, and blow cooler air in at night.
- Listen to your NOAA all-hazards weather radio to keep up with the latest heat watches, warnings and advisories.

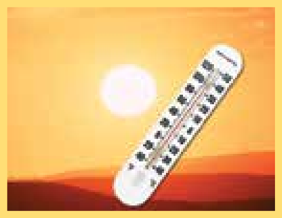

Drought

Droughts are unique among natural disasters. We don't know we're in a drought until weeks after it begins, making preparation difficult. Once a drought is underway, water conservation is the only way we can lessen its effect.

Water conservation tips

- Fix leaky faucets and plumbing joints.
- Install water-saving shower heads and take shorter showers.
- Run only full loads in the washing machine and dishwasher and use the shortest wash cycle.
- Turn off the water while brushing teeth, shaving, etc.
- Chill drinking water in the refrigerator instead of letting the tap run.
- Use a broom instead of a hose to clean driveways and sidewalks.
- Water your lawn only when it needs it, and water during the cool parts of the day.
- Adjust sprinklers so that water lands on your lawn, not on pavement.
- Put a layer of mulch around trees and plants.
- Use a rain barrel to collect stormwater runoff from downspouts for reuse.
- Landscape with native plants that require less water.

Winter Weather

Winter weather can cause many dangerous conditions, including cold temperatures, snow and ice storms. Before winter sets in, take time to winterize your home with storm windows, weather stripping and insulation.

Winter Storm Safety Tips

- Stay indoors during the storm if possible.
- If you must go outside, dress appropriately.
- Walk carefully on snowy, icy sidewalks.
- Avoid overexertion, especially when shoveling snow.
- If your heat goes off, close off unused rooms and use blankets to seal off drafts from doors and windows. Dress in layers, and keep your hands and feet warm.

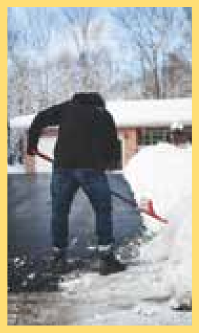

If you must travel by car during a storm:

- Take emergency supplies, including thermal blankets and a cell phone.
- Keep your gas tank full.
- Let someone know your destination, your route, and when you expect to arrive.
- If you get stuck, stay with your car. Do not try to walk to safety. Start the car and use the heater for about 10 minutes every hour.

Food Safety/Power Outages

Food safety is important to your family's health anytime, but especially during a power outage.

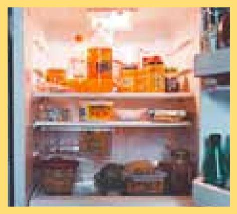

A full freezer will stay frozen for about two days, and a half-full freezer for about one day. Keep your freezer door closed as much as possible to conserve the cold.

Refrigerated foods should be safe if power is not out more than four hours.

Food Safety Guidelines

- Freezer food may be safely refrozen when power is restored, if it still contains ice crystals or is at 40° F or below.
- As food thaws, separate raw meat products from other items.
- Foods such as meat, poultry, seafood, dairy products, creamy dressings, eggs, cooked pastas, casseroles, soups, cut fruit and vegetables should to be discarded if power is out more than just a few hours.
- Hard or processed cheeses, butter, margarine, peanut butter, jelly, whole fruit, vegetables, and vinegar-based dressings should be safe.

Remember: When in doubt, throw it out.

Portable Generator Safety

Portable electric generators offer great benefits when outages affect your home, but can also be hazardous. The primary hazards to avoid when using a generator are carbon monoxide (CO) poisoning from toxic engine exhaust, electric shock and fire.

Safety Tips:

- Never use a generator indoors or in an attached garage.
- Don't connect your generator directly to your home's wiring, or to a regular household outlet.
- Don't overload the generator.
- Use the proper power cords.

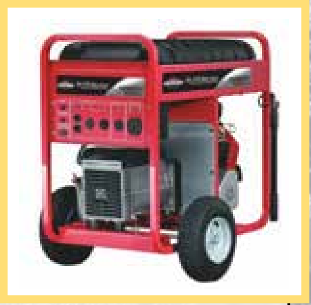

- Read and adhere to the manufacturer's instructions for safe operation.
- To prevent electrical shock, make sure your generator is properly grounded.
- Do not store fuel indoors or try to refuel a hot generator or one that is running.
- Turn off all equipment powered by the generator before starting or shutting down your generator.
- Avoid getting burned.
- Keep children away from portable electric generators at all times.

Carbon Monoxide Dangers

Carbon monoxide (CO) is a deadly colorless and odorless gas produced by burning things. It is poisonous to people and animals. To prevent CO poisoning, never use unvented grills or generators indoors. Faulty heating systems can also produce CO.

Test and replace the batteries in your CO detector twice a year when you check your smoke detectors.

Protect your family by installing a CO detector according to manufacturer's instructions. Have your fuel-burning appliances — including heaters, stoves and fireplaces — inspected each year.

The initial symptoms of CO poisoning are similar to the flu (but without fever). They include headache, fatigue, shortness of breath, nausea and dizziness. Many people with CO poisoning mistake their symptoms for the flu.

If you think you or your family are experiencing any of the symptoms of CO poisoning, immediately leave the house with your family and pets (pets are in danger also), go outside to fresh air and call 911.

Fire

In the event of a fire, remember — time is the biggest enemy and every second counts! Escape plans help you get out of your home quickly.

Create and practice a family fire escape plan

- Plan two ways out of every room, and practice your escape plan regularly.
- Make sure everyone in the family knows how to open locked or barred doors and windows and that everyone can exit out of windows or second stories safely to the ground.
- When a fire occurs, leave immediately. Don't waste time saving property.

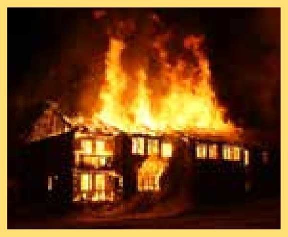

- If you must escape through smoke, crawl low and cover your mouth.
- Never open doors that feel hot. Use your secondary escape route.
- Designate a meeting location away from the home, but not too far. Make sure everyone has gotten out safely and no one will be hurt looking for someone who is already safe.
- Install working smoke alarms on all levels of your home and in every sleeping area. Test batteries every month and change them twice a year.
- If your smoke detector is more than 10 years old, replace it.

Hazardous Materials/Shelter in Place

In an emergency where hazardous materials may have been released into the atmosphere, authorities may ask you to shelter in place — to take refuge in a small, interior room with few or no windows. If the need to shelter in place ever arises, you will hear announcements on TV, radio and NOAA all-hazards weather radio.

If you are told to shelter in place:

- Take action immediately and go indoors.
- Close and lock all windows and exterior doors.
- Turn off all fans, heating and air conditioning systems. Close fireplace dampers.
- Get your family disaster supplies kit and make sure your battery-operated radio is working.
- Gather people and pets in an interior room at or above ground level with few / no windows.
- Use duct tape and plastic sheeting (heavier than the kind used to wrap food) to seal around the door and vents.
- Keep listening to your radio or TV for an "all clear" or further instructions.

Sheltering-in-place is meant to protect you and your family for just a few hours.

If you are asked to evacuate, please refer to page 30 for tips.

Terrorism/Active Shooter

Be aware of suspicious activity

You can help prevent and detect terrorism — and other types of crime — by watching for suspicious activities and reporting them to the proper authorities.

Signs to watch for:

1. Observation / surveillance / photography.
2. Expressed or implied threat.
3. Materials acquisition / storage.
4. Weapons collection.
5. Eliciting information.
6. Testing or probing of security.
7. Attempted intrusion.
8. Misrepresentation.
9. Theft / loss / diversion.
10. Sabotage / tampering / vandalism.

If you SEE something, SAY something.

To report suspicious activity, contact your local law enforcement agency or, to remain anonymous, call 816-474-TIPS. Describe specifically what you observed, including who or what you saw, when and where you saw it and why it's suspicious.

Know what to do

In an emergency, call 911 when it's safe to do so. In an active shooter incident, take action based on the situation.

- Flee the threat — Escape immediately if you can.
- Hide / fortify — If you can't run, lock / block the door and conceal yourself behind a solid object.
- Fight — As a last resort; commit to you actions.
- Stop the bleed — Assist injured persons when safe to do so. Find bleeding source, pack wound with clean cloth and apply continuous pressure.

Transportation Safety

Unsafe driving behaviors increase the risk of roadway fatalities and serious injuries. Reduce your risk of death and injury by being informed and following some simple rules:

- Always wear a seatbelt. Insist that passengers are buckled up too.
- Do not drive aggressively. Slow down, drive wisely and obey speed limits, especially in school and construction zones.
- Don't drink and drive. Have a designated driver or use a transportation provider.
- Don't use your phone while driving. Focus on the road. If you have to use your phone, pull over to a safe location to avoid distractions.
- Be safe, be seen and follow the rules of the road.

Traveling by air? Visit www.tsa.gov for the latest security tips.

If you are in an accident:

- Pull off the road, if it's possible to do so safely.
- Turn on hazard lights.
- Stay in your vehicle until help arrives.
- If someone stops, crack your window and ask them to call the police.

Pandemic / Infectious Disease

Influenza, or flu, is a contagious respiratory disease that is caused by viruses. Seasonal flu occurs every year, typically in the fall and winter. Pandemic flu is different and can be much worse. It can cause a worldwide outbreak of a new form of flu virus, which spreads easily from person to person because people have no immunity.

Protect yourself and others

- Everyone six months of age and older should get a flu vaccine during flu season. Keep all immunizations up to date.
- Avoid close contact with people who are sick.
- Stay home when you're sick or have flu-like symptoms.
- Cover your mouth and nose when coughing or sneezing. If no tissue is available, direct the cough or sneeze into the bend of your elbow, not into your hands.
- Wash your hands often to protect against germs.
- Avoid touching your eyes, nose or mouth. Germs are often spread when a person touches something that is contaminated with germs, then touches his or her eyes, nose or mouth.
- Practice good health habits. Get plenty of sleep, be physically active, manage stress, drink plenty of fluids, eat nutritious foods and avoid smoking.
- Follow guidance from local health departments or visit www.cdc.gov. Keep an emergency contact list for family, friends or others who might need help.

First Aid

Knowledge of basic first aid is an important part of emergency preparedness. Consider taking a certified CPR and first-aid class from your local American Red Cross chapter, local hospital or other community organization. Keep basic first-aid supplies in your family's disaster supply kit:

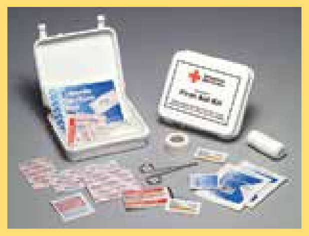

- Sterile bandages, gauze pads, adhesive tape, cotton balls.
- Elastic bandages for sprains.
- Aspirin and non-aspirin pain relievers, cough syrup, antihistamines, decongestants, ointment.
- Antacids and anti-diarrhea medication.
- Thermometer.
- Shears, tweezers, safety pins.
- Alcohol wipes, antibiotic towelettes or cleansing agent / soap to clean wounds.
- Iodine or hydrogen peroxide.
- Disposable gloves.
- A 30-day supply of prescription drugs and other supplies such as insulin and needles, glucose strips or tablets, and oxygen tanks.
- Emergency mylar heat blanket.
- Tourniquet.
- A list of family members' allergies, health conditions and medicines (See page 33).

Make an Emergency Plan

Meet with your family and discuss why you need to prepare for disaster. Explain the dangers of fire, severe weather, and earthquakes to children. Include your pets in your plan, share responsibilities and work together as a team.

- Discuss the types of disasters that are most likely to happen. Learn what to do in each case.
- Pick two places to meet:
  - Right outside your home in case of a sudden emergency, like a fire.
  - Outside your neighborhood in case you can't return home.
- Make sure everyone knows the addresses and phone numbers of your meeting places.
- Ask an out-of-state friend to be your "family contact." After a disaster, it's often easier to call long distance than local.
- Check on the emergency plans of all schools your children attend.

Practice and maintain your plan

- Quiz your kids every six months.
- Conduct fire and emergency drills.
- Replace stored water and stored food every six months.

- Test and recharge your fire extinguisher(s) regularly.
- Test your smoke detectors monthly and change the batteries twice a year.

Disaster Plan Checklist

- Post emergency numbers by phones (ambulance, police, fire, etc.).
- Teach children how and when to call 911 for emergency help.
- Show each family member how and when to turn off the utilities (water, gas, and electricity).
- Check your insurance coverage.
- Take Community Emergency Response Team (CERT), first aid and CPR training. Learn how to use a fire extinguisher (ABC type), and show family members where it's kept.
- Install smoke detectors on each level of your home, especially in bedrooms.
- Conduct a home hazard hunt.
- Stock emergency supplies and assemble a disaster supplies kit.
- Take first aid, CPR and "You're the Help Until Help Arrives" classes. Check with your local Emergency Managers for locations.
- Determine the best escape routes from your home. Find two ways out of each room.
- Find the safe places in your home for each type of disaster.

Create a family emergency plan now, before disaster strikes. Learn more at www.redcross.org/get-help/how-to-prepare-for-emergencies/make-a-plan

Create a Disaster Supply Kit

Keep supplies in an air-tight, easy-to-carry container, such as a large, covered trash can, a backpack or a duffle bag. Rotate your stored food and water supply every six months so it stays fresh.

Re-think your family needs and refresh your kit at least once a year, replacing batteries and updating clothing and other supplies.

Water

- ☐ Store one gallon of water per person per day.
- ☐ Keep at least a three-day supply of water.

Food

Store at least a three-day supply of non-perishable foods that require no refrigeration, preparation or cooking.

- ☐ Canned meats, fruits and vegetables, juices, soups.
- ☐ Basics — salt, sugar, pepper.
- ☐ High energy foods — peanut butter, jelly, crackers, granola bars, trail mix.
- ☐ Food for infants, the elderly, or special diets.
- ☐ Comfort / stress foods — cookies, candy, cereals, instant coffee, tea bags.
- ☐ Store extra pet food for animals.

First aid kit

Assemble a first aid kit for your home and one for each car. A first aid kit should include the items on page 19.

Tools and supplies

- ☐ Flashlight, battery-operated radio and extra batteries.
- ☐ Non-electric can opener, utility knife, multi-tool.
- ☐ Matches, signal flare, fire extinguisher.
- ☐ Wrench to turn off household gas and water.
- ☐ Duct tape, plastic sheeting.

Clothing and bedding

- ☐ Sturdy shoes or work boots.
- ☐ Rain gear, hats, gloves.
- ☐ Blankets or sleeping bags.

Special items

- ☐ Baby formula, diapers, bottles.
- ☐ Face masks, hand sanitizer and other personal hygiene supplies.
- ☐ Prescription drugs.
- ☐ Pet emergency supplies. (See page 27.)
- ☐ Games and entertainment.
- ☐ Cash.

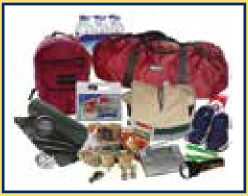

For more detailed guidelines on what to include, visit www.redcross.org

Important family documents

Keep these records in a waterproof, portable container:

- ☐ Wills, insurance policies, deeds, stocks and bonds.
- ☐ Passports, identification, immunization records.
- ☐ Bank account numbers.
- ☐ Credit card account numbers and companies.
- ☐ Inventory of valuable household goods, important telephone numbers.
- ☐ Family records (birth, marriage, death certificates).

Older Adults

The Kansas City region's older adult population is growing. Older adults may be more vulnerable in disasters due to increased social isolation, decreased sensory awareness, physical impairment, chronic medical conditions and socioeconomic limitations.

Preparedness Tips

- Assemble a disaster supplies kit. (See page 22.)
- Make and emergency plan. This checklist is pertinent for all citizens. (See page 20.)
- Arrange for someone to check on you regularly.
- Plan and practice the best escape routes from your home.
- Plan for transportation if you need to evacuate to a shelter.
- Find the safe places in your home for each type of emergency.
- Have a plan to signal the need for help.
- Post emergency phone numbers near the phone.
- If you have home healthcare service, plan with your service provider for emergency procedures.
- Teach those who may need to assist you in an emergency how to operate necessary equipment; be sure they will be able to reach you.

In some communities, people who need help or transportation during an evacuation may need to register with their local government. Call your local emergency management office for information and suggestions about what to do during an evacuation.

Children and their Trusted Adults

Considering that children comprise approximately 25 percent of our population, disaster planning, response and recovery efforts must consider the unique needs that children have. Children's response to a disaster relies on calm, prepared and trained adults. Children who practice preparedness are more confident during emergencies and disasters.

Tips for Children and their Trusted Adults

- Assemble a disaster supplies kit. (See page 22.)
- Make and emergency plan. This checklist is pertinent for all citizens. (See page 20.)
- Children need their own emergency kit. Get them involved in building their own. Children's kits should include toys, games, comfort items and contact information.
- Create a child identification card, with fingerprint and current photo (remember to update it regularly), and keep it in your kit.
- Put child-sized dosages of over-the-counter medication in your first aid and emergency kits.
- Know the emergency plan at your child's childcare provider, babysitter or school.
- Practice your emergency plan and how to call 9-1-1 with your child.
- Teach children to approach first responders in a disaster in case they are separated from family.

Access and Functional Needs

If you have a disability, you should be ready to meet your specific disability-related needs for at least seven days after a disaster. Your usual means of support and assistance may not be available for a varying duration of time based on a given disaster event.

Your personal disaster plan

Make a personal disaster plan to help organize the information you will need and actions to take during and after a disaster. Share your plan with your family, friends and caregivers.

- Assemble a disaster supplies kit. (See page 22.)
- Make and emergency plan. This checklist is pertinent for all citizens. (See page 20.)
- Make a list of accessible transportation services that meet your needs.
- Develop a support network of individuals who can assist you after a major disaster. If you need disaster recovery assistance, call 2-1-1.
- Create a list of your medical providers, medications and dosages, when you take each medication, any adaptive equipment you use, your allergies and sensitivities, and any communication or cognitive difficulties you may have. Attach copies of health insurance cards and related information. (See page 33.)

To organize your emergency planning, use our Personal Preparedness Inventory booklet. Find it at www.marc.org/Emergency-Services-9-1-1/pdf/EP_Workbook_Web_062116.aspx

Pet Safety

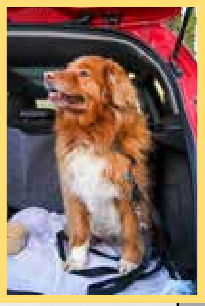

Different disasters require different responses, but in some cases you may have to evacuate your home. So prepare now for the day when both you and your pets may have to evacuate.

In a disaster, separate pet shelters will be established by local authorities, but supplies may not be available immediately, so plan ahead and make a pet supply kit. Get your pet microchipped now.

Pet supply kit

- A picture of yourself with your pet.
- Your pet's medical records.
- Collar with ID tag, harness, leash, pet carrier.
- Pet food and water.
- Hygiene and sanitation items such as litter box and litter, newspapers, paper towels, plastic bags.

Pet evacuation plans

- Locate hotels and motels outside your immediate area that will accept pets.
- Ask friends, relatives or others outside your area whether they could shelter your animals.
- Prepare a list of boarding facilities and veterinarians who could shelter animals in an emergency; include 24-hour phone numbers.

Planning and preparation will enable you to evacuate with your pets quickly and safely.

Web Resources

For more information about emergency preparedness, please visit the following websites:

- **www.preparemetrokc.org**
- **www.ready.gov**
- **www.fema.gov**
- National Weather Service
  www.weather.gov
- Kansas Division of Emergency Management
  www.kansastag.gov/kdem_default.asp
- Missouri State Emergency Management Agency
  http://sema.dps.mo.gov
- Kansas Homeland Security
  www.ksready.gov
- Missouri Homeland Security
  http://dps.mo.gov/dir/programs/ohs
- Missouri "Ready in 3" program
  https://health.mo.gov/emergencies/readyin3
- United Way 2-1-1
  www.unitedwaygkc.org

Get Involved

You can get involved in preparedness efforts in your community. Many local agencies are seeking volunteers.

- Amateur Radio — Metropolitan Emergency Communications Council
- American Red Cross
  www.redcross.org
- Community Emergency Response Teams (CERT)
- Community Disaster Resiliency Network (CDRN)
  www.marc.org/Emergency-Services-9-1-1/Preparedness/Community-Disaster-Resiliency-Network-Initiatives/Overview
- Fire Corps
- Medical Reserve Corps of Greater Kansas City
  www.mrckc.org
- Neighborhood Watch
- Team Rubicon USA
  www.teamrubiconusa.org
- Salvation Army
  www.salarmy-mokan.org
- Volunteers in Police Service

Your Evacuation Plan

If you are instructed to evacuate because of a disaster or emergency, you will need to do so quickly. Talk to your family about evacuation plans now.

Potential meeting places (both nearby and outside your neighborhood):

____

____

Out of town contact person (useful when local phone service is disrupted):

____

____

Pet arrangements:

____

____

Last minute checklist:

- ☐ Take a battery-powered radio.
- ☐ Wear protective clothing and sturdy shoes.
- ☐ Take your disaster supplies kit. (See page 22.)
- ☐ Lock your doors.
- ☐ Leave your home. Do not take the time to shut off water, gas and electricity.
- ☐ Let someone know where you are going.

Your Utilities

Gas

Service Provider ____________________

Contact Information ____________________

Shutoff Location ____________________

Instructions ____________________

Electricity

Service Provider ____________________

Contact Information ____________________

Shutoff Location ____________________

Instructions ____________________

Water

Service Provider ____________________

Contact Information ____________________

Shutoff Location ____________________

Instructions ____________________

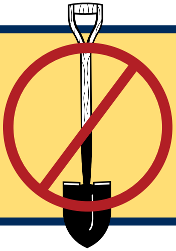

Call Before You Dig!

Missouri: 1-800-DIG-RITE

Kansas: Call 8-1-1

Home Hazard Hunt

In a disaster, ordinary items in the home can cause injury and damage. Anything that can move, fall, break or cause a fire is a potential hazard.

- Repair defective electrical wiring and leaky gas connections.
- Fasten shelves securely.
- Place large, heavy objects on lower shelves.
- Hang pictures and mirrors away from beds.
- Brace overhead light fixtures.
- Secure water heater. Strap to wall studs.
- Repair cracks in ceilings or foundations.
- Store weed killers, pesticides, and flammable products away from heat sources.
- Place oily polishing rags or waste in covered metal cans.
- Clean and repair chimneys, flue pipes, vent connectors and gas vents.

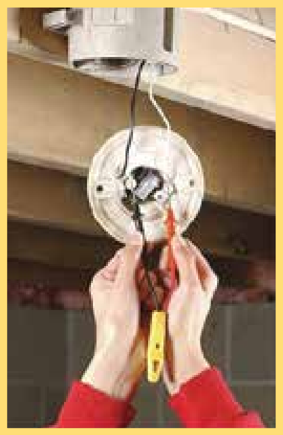

- Don't use extension cords for extended periods of time.
- Hire a professional to repair chimneys, foundations, electrical wiring, gas lines.

Family Medical Information

List information for each family member. Use additional sheets of paper if needed.

Name: ______________________________

Allergies: ______________________________

Medical conditions: ______________________

Prescriptions: ___________________________

____

Name: ______________________________

Allergies: ______________________________

Medical conditions: ______________________

Prescriptions: ___________________________

____

Name: ______________________________

Allergies: ______________________________

Medical conditions: ______________________

Prescriptions: ___________________________

____

Important Numbers

Doctor(s) ______________________________

____

Hospital ______________________________

Pharmacy ______________________________

Poison Control ______________________________

Insurance:

Medical ______________________________

Home ______________________________

Auto ______________________________

Other important numbers:

____

____

____

____

Not sure who to call for help? Start with United Way 2-1-1 — one number that can connect you with community resources and volunteer opportunities around the region.

United Way 2-1-1 Get Help. Give Help.

Local Emergency Numbers

Police Department ________________________

Fire Department ________________________

Ambulance ________________________

City Hall ________________________

Emergency Management ________________________

City/County Health Department ________________________

Other:

____

____

____

____

____

____

____

____

____

____

____

In an emergency, call 911.

Metropolitan Emergency Management

The following are a list of local city and county emergency managers. Call the numbers below to learn more about the resources available, or how you can get involved in volunteer and training opportunities.

County Emergency Management

Cass County, Missouri 816-380-8584
Clay County, Missouri 816-407-3730
Jackson County, Missouri 816-881-4625
Johnson County, Kansas 913-782-3038
Leavenworth County, Kansas* 913-684-0455
Miami County, Kansas 913-294-4444
Platte County, Missouri 816-858-3521
Ray County, Missouri 816-776-4507
Wyandotte County, Kansas 913-573-6300

City Emergency Management

Belton, Missouri 816-331-5522
Buckner, Missouri 816-650-3191
Fairway, Kansas 913-782-0720
Edgerton, Kansas 913-893-6231
Gardner, Kansas 913-856-7312
Gladstone, Missouri 816-423-4081
Grandview, Missouri 816-457-0791
Independence, Missouri 816-325-7167
Kansas City, Missouri 816-513-8640
Lake Lotawana, Missouri 816-541-8017
Lansing, Kansas 913-727-3000
Leawood, Kansas 913-681-6788
Lee's Summit, Missouri 816-969-1300
Lenexa, Kansas 913-888-6380
Liberty, Missouri 816-439-4701
Merriam, Kansas 913-322-5560
Olathe, Kansas 913-971-7974

Overland Park, Kansas	913-895-8308
Parkville, Missouri	816-741-4454
Pleasant Hill, Missouri	816-540-9109
Prairie Village, Kansas	913-642-6868
Raymore, Missouri	816-892-3032
Raytown, Missouri	816-737-6020
Riverside, Missouri	816-741-1191
Shawnee, Kansas	913-742-6139
Weatherby Lake, Missouri	816-741-8111
West Peculiar Emergency Management	816-779-5766

Fire Protection Districts

Central Jackson County Fire Protection District	816-229-2522
Oak Grove/Sni Valley Fire Protecton District	816-834-1625
South Metro Fire Protection District	816-331-3008

Other Agencies

American Red Cross	816-931-8400
Kansas Division of Emergency Management	785-646-1409
Missouri State Emergency Management Agency (SEMA)	573-526-9100
National Weather Service	816-540-6021
Salvation Army	816-756-1455

A current list of MEMC member organizations is available on MARC's website at **www.marc.org/memcmembers**.

** Contact Leavenworth County Emergency Management for cities of Lansing, Tonganoxie and Basehor.*

If you don't find your city or county listed here, call your city hall or county offices and ask for the emergency management department.

The Metropolitan Emergency Managers Committee includes emergency managers from cities and counties in the Greater Kansas City region.

This publication produced with funds provided by the U.S. Department of Homeland Security

Metropolitan Emergency Managers Committee
c/o Mid-America Regional Council
600 Broadway, Suite 200
Kansas City, MO 64105-1659

Printed August 2020
