# Supplementary material for: Disaster Preparedness Intervention for Older Adults (Seniors’ Positive Involvement in Community Emergencies): Protocol for a Quasi-Experimental Study
Source: JMIR Res Protoc. 2024 Dec 4;13:e58895. doi: 10.2196/58895 (PMC11656111; doi:10.2196/58895)
Supplement: Multimedia Appendix 3 [file resprot_v13i1e58895_app3.pdf]

AmeriCorps SPICE UMKC

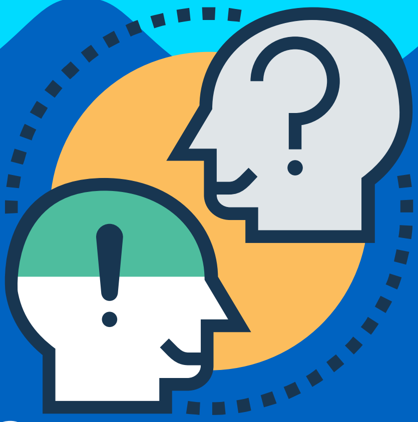

My Communication For Disaster Recovery

My Neighborhood Organization

My Neighbor/Friend/Relative

My Library

Independence Emergency Management

950 N Spring St
(816) 325-7167

Independence Health Department

111 E Maple Ave
(816) 325-7803

KC Health Department

2400 Troost Ave
(816) 513-6008
kcmo.gov/city-hall/departments/health

KC Emergency Management

KC EM
635 Woodland Ave
Suite 2107
(816) 513-8640
OEM@KCMO.org

Call 211

United Way
Staffed 24/7
Emergency
Resource Call Line

Aging and Adult Services

marc.org/aging-health/aging-and-adult-services

02/09/24 (RC)
