# Supplementary material for: Disaster Preparedness Intervention for Older Adults (Seniors’ Positive Involvement in Community Emergencies): Protocol for a Quasi-Experimental Study
Source: JMIR Res Protoc. 2024 Dec 4;13:e58895. doi: 10.2196/58895 (PMC11656111; doi:10.2196/58895)
Supplement: Multimedia Appendix 4 [file resprot_v13i1e58895_app4.pdf]

My Action Steps to Avoid:

Disaster Recovery Scams

Know YOUR FEMA Application Number:

Applying for FEMA Aid is Always FREE!

Federal, State, U.S. Small Business Administration, and FEMA Personnel NEVER Charge.

FEMA HELP LINE
(800) 621-3372

Region 7

Disaster.Assistance.gov

NEVER pay upfront for ANY repairs. Have a Written Contract First with No blanks.

Do NOT provide personal information over the phone including bank account.

I WILL BE A DISASTER SURVIVOR!$

Most Contractors will require a REASONABLE down payment AFTER the written contract.

There is No Such Thing as "FEMA Certified"

FEMA neither certifies nor endorses any private-sector contractor.

Written Contracts should include everything the contractor will do and prices for labor, materials, clean up.

Written Contracts should include estimated start and finish dates.

FEMA Reps carry a laminated photo ID Badge. Ask to See Identification.

Non-Profits Work at No Cost. Disaster Relief Workers Will Not Ask for Money.

Confirm Status of Charities with Charity Navigator.org

Report SCAMS to www.justice.gov/DisasterComplaintForm

Adapted From: teamrubiconusa.org/news-and-stories/tips-to-avoid-disaster-relief-scams/
AmeriCorps SPICE UMKC draft (RC) 1/26/2024
