# Supplementary material for: Disaster Preparedness Intervention for Older Adults (Seniors’ Positive Involvement in Community Emergencies): Protocol for a Quasi-Experimental Study
Source: JMIR Res Protoc. 2024 Dec 4;13:e58895. doi: 10.2196/58895 (PMC11656111; doi:10.2196/58895)
Supplement: Multimedia Appendix 5 [file resprot_v13i1e58895_app5.pptx]

## Slide 1
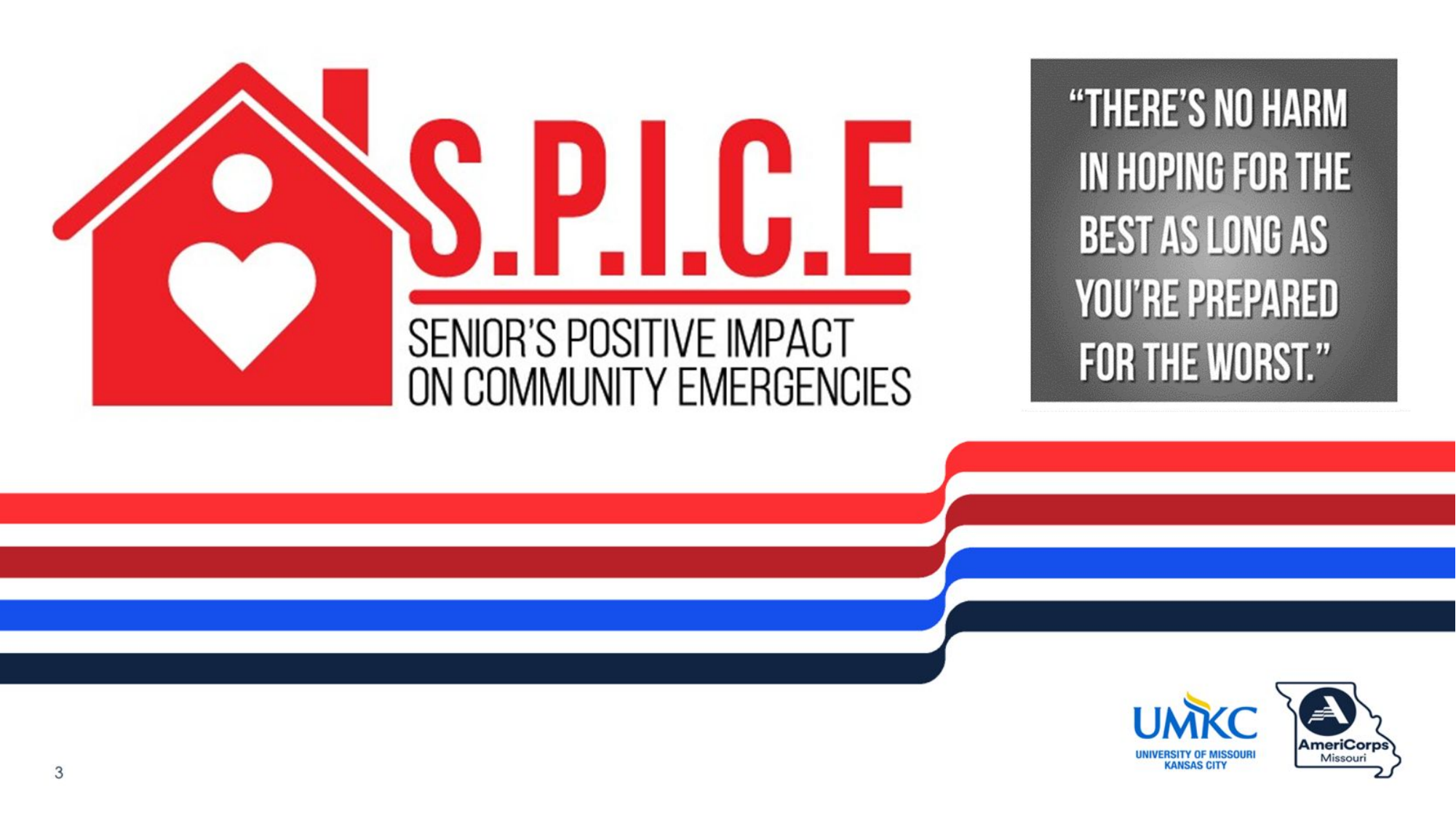

## Slide 2
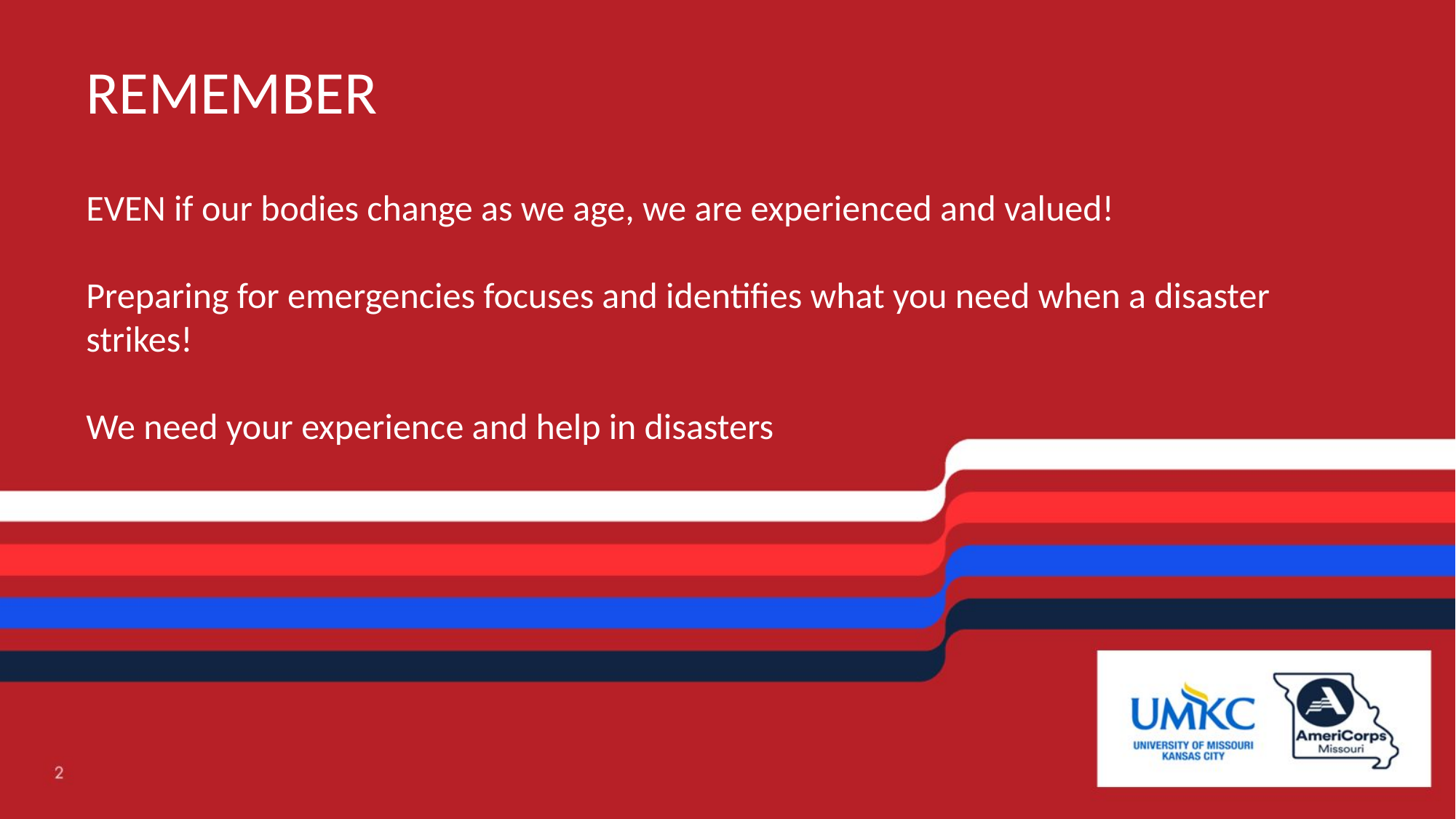

REMEMBER
EVEN if our bodies change as we age, we are experienced and valued!
Preparing for emergencies focuses and identifies what you need when a disaster strikes!
We need your experience and help in disasters

## Slide 3
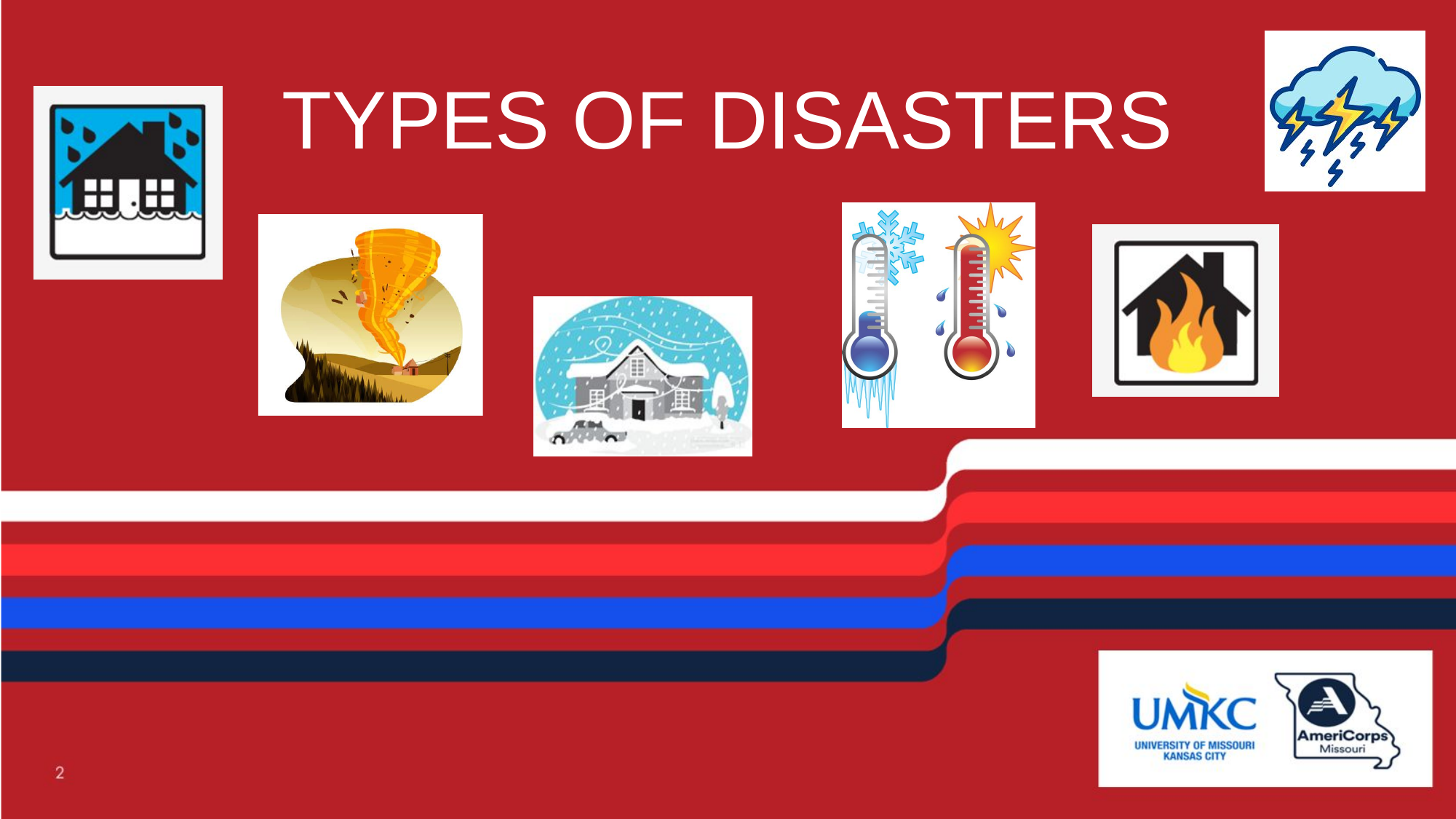

# TYPES OF DISASTERS

## Slide 4
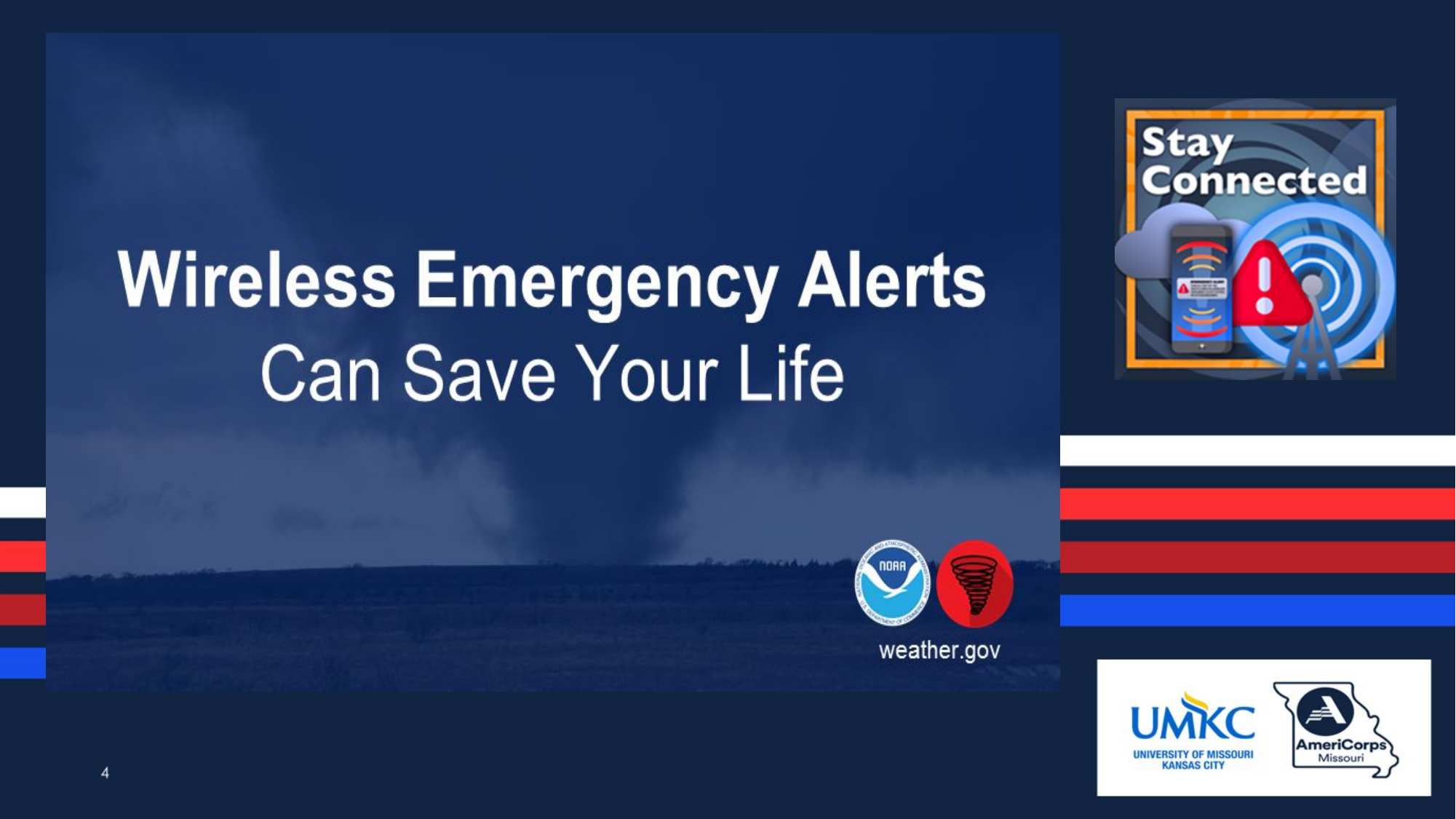

## Slide 5
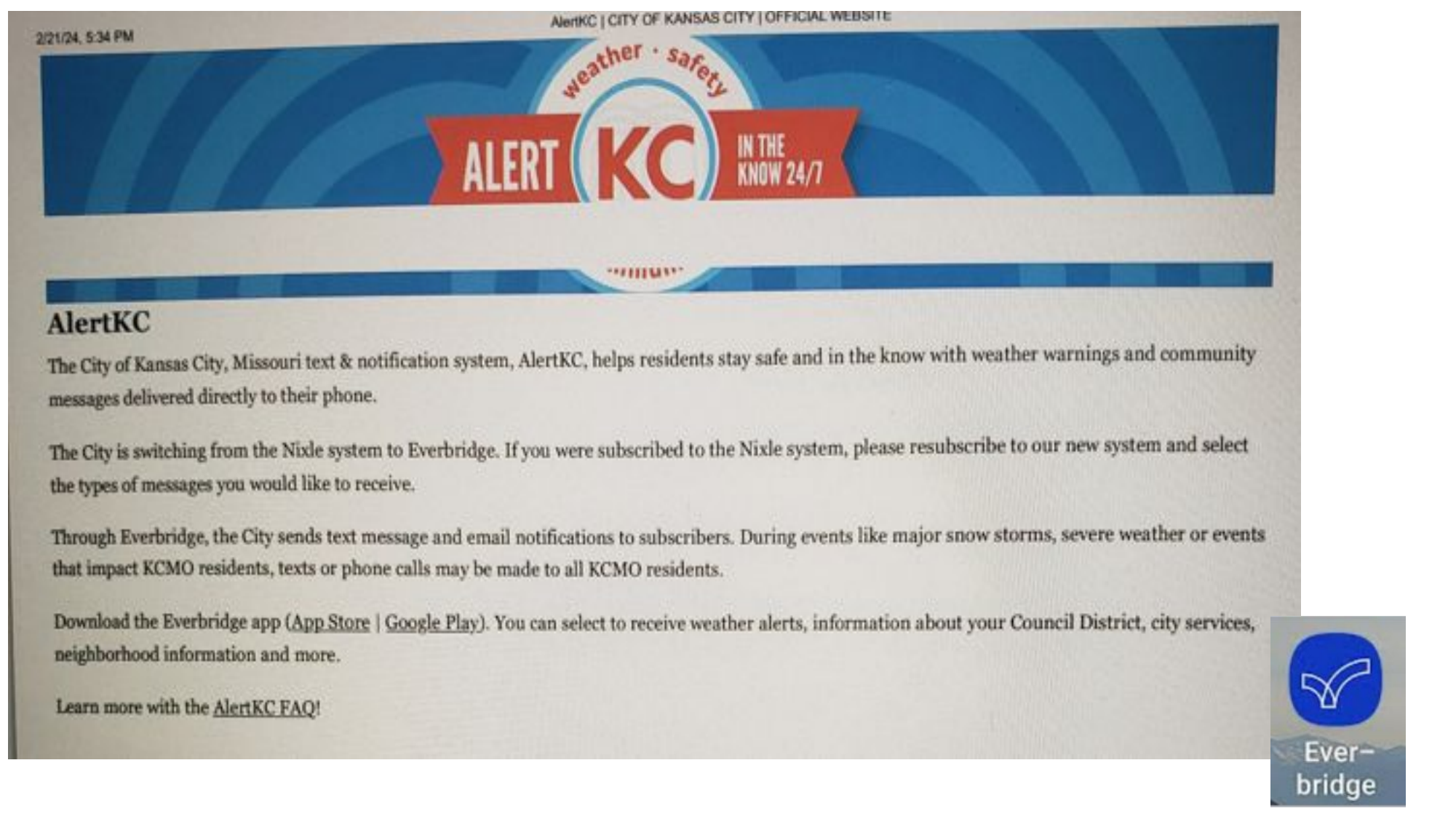

## Slide 6
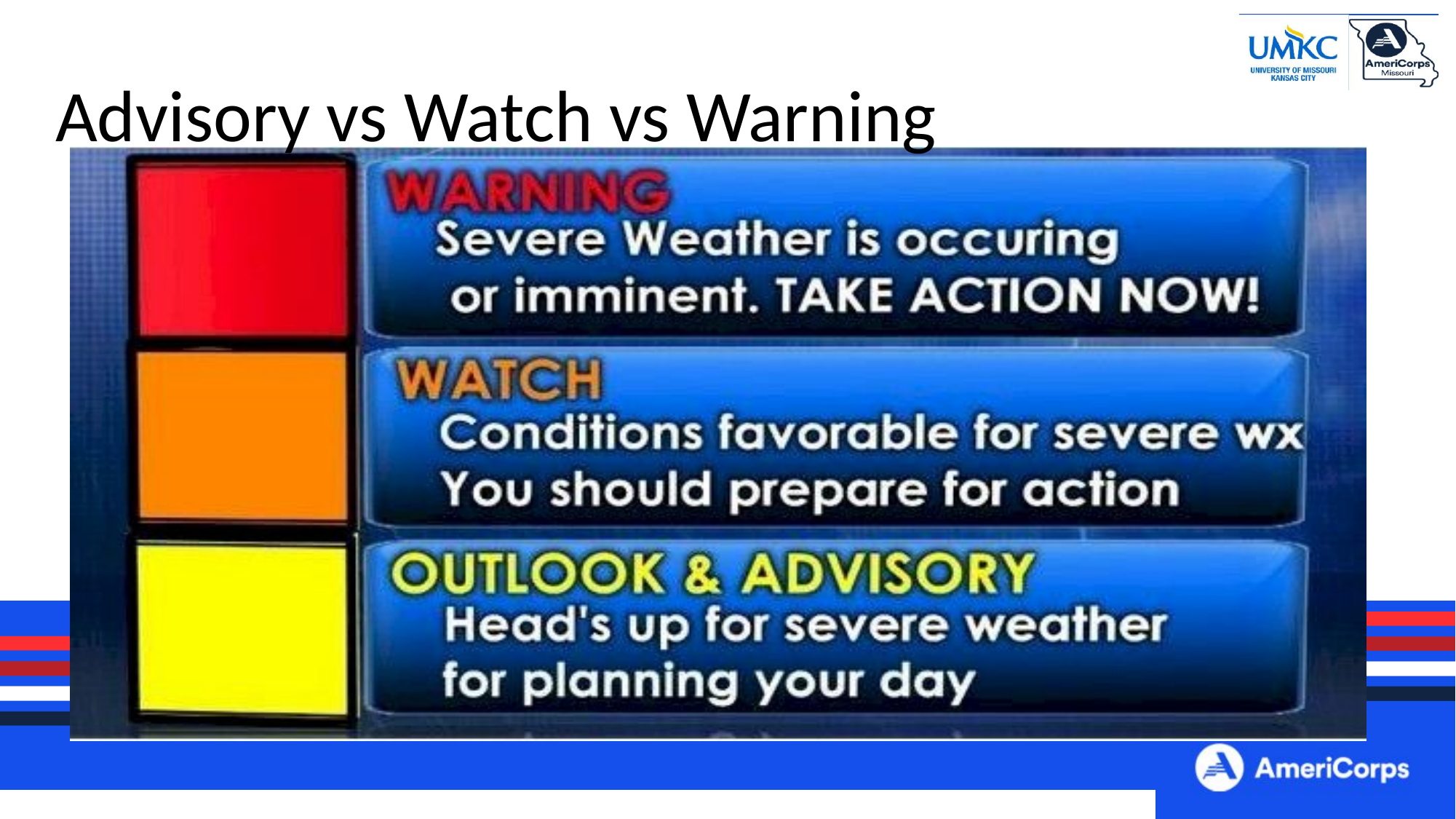

Advisory vs Watch vs Warning

## Slide 7
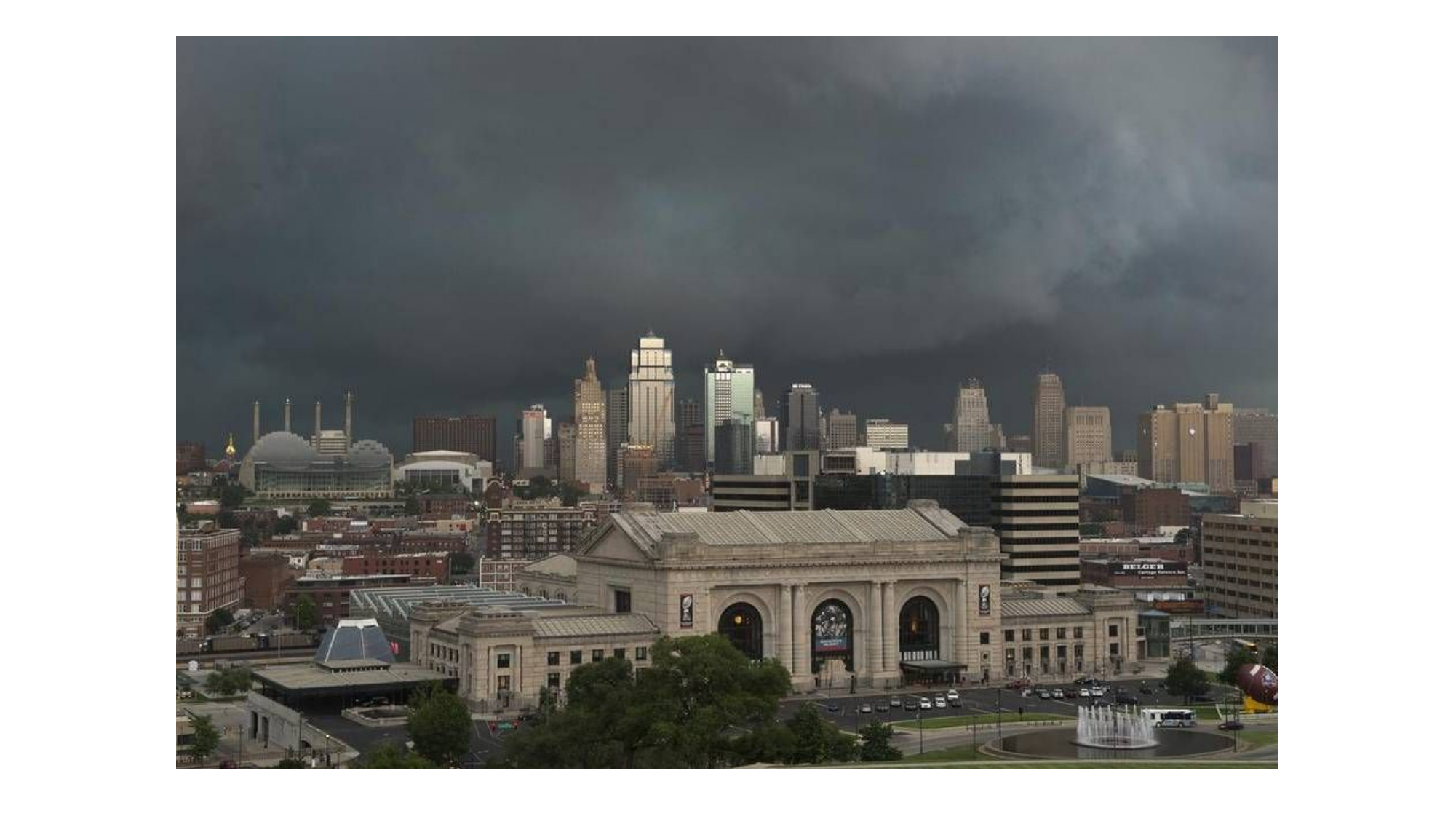

## Slide 8
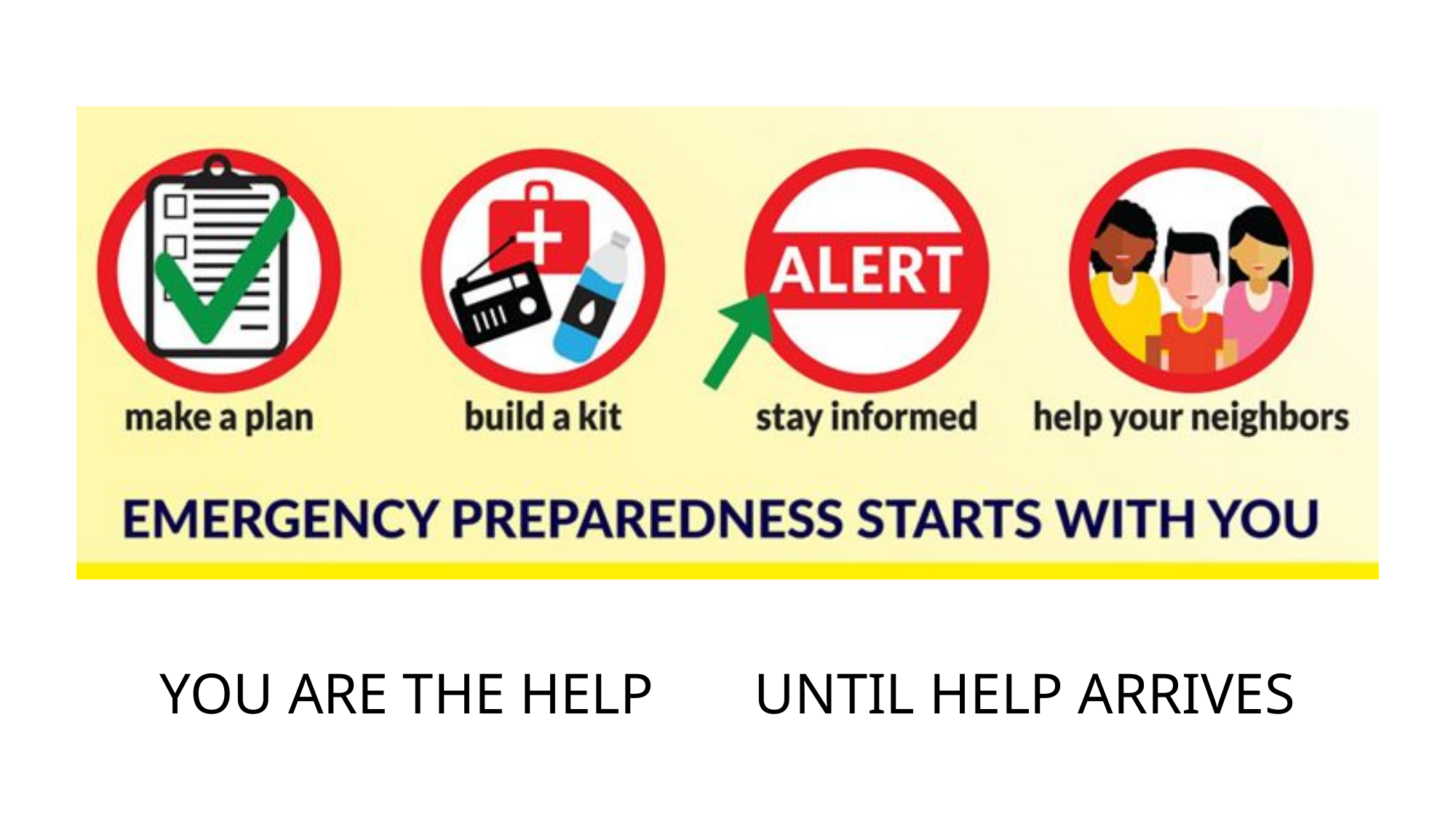

YOU ARE THE HELP UNTIL HELP ARRIVES

## Slide 9
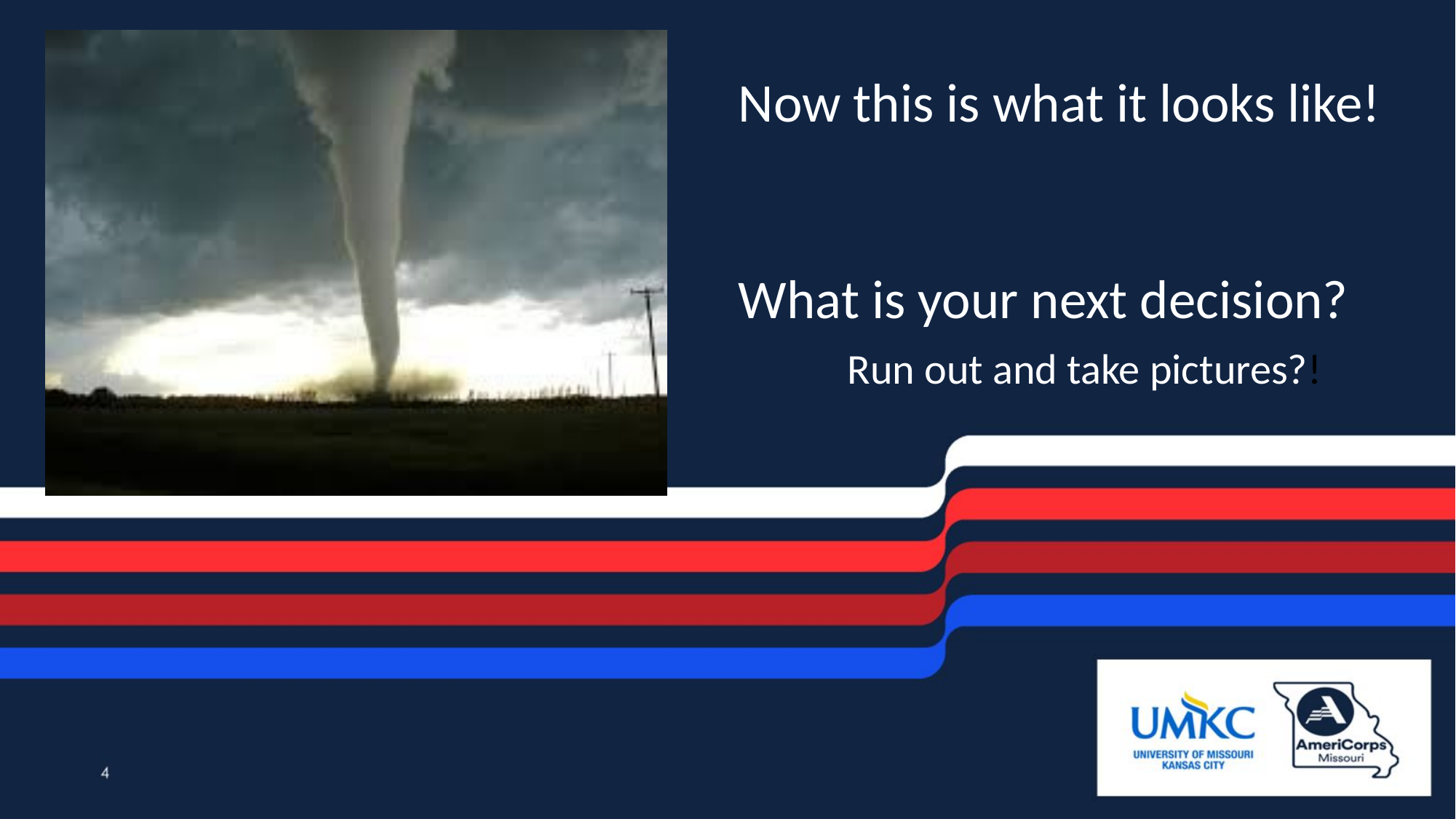

Now this is what it looks like!
What is your next decision?
	Run out and take pictures?!

## Slide 10
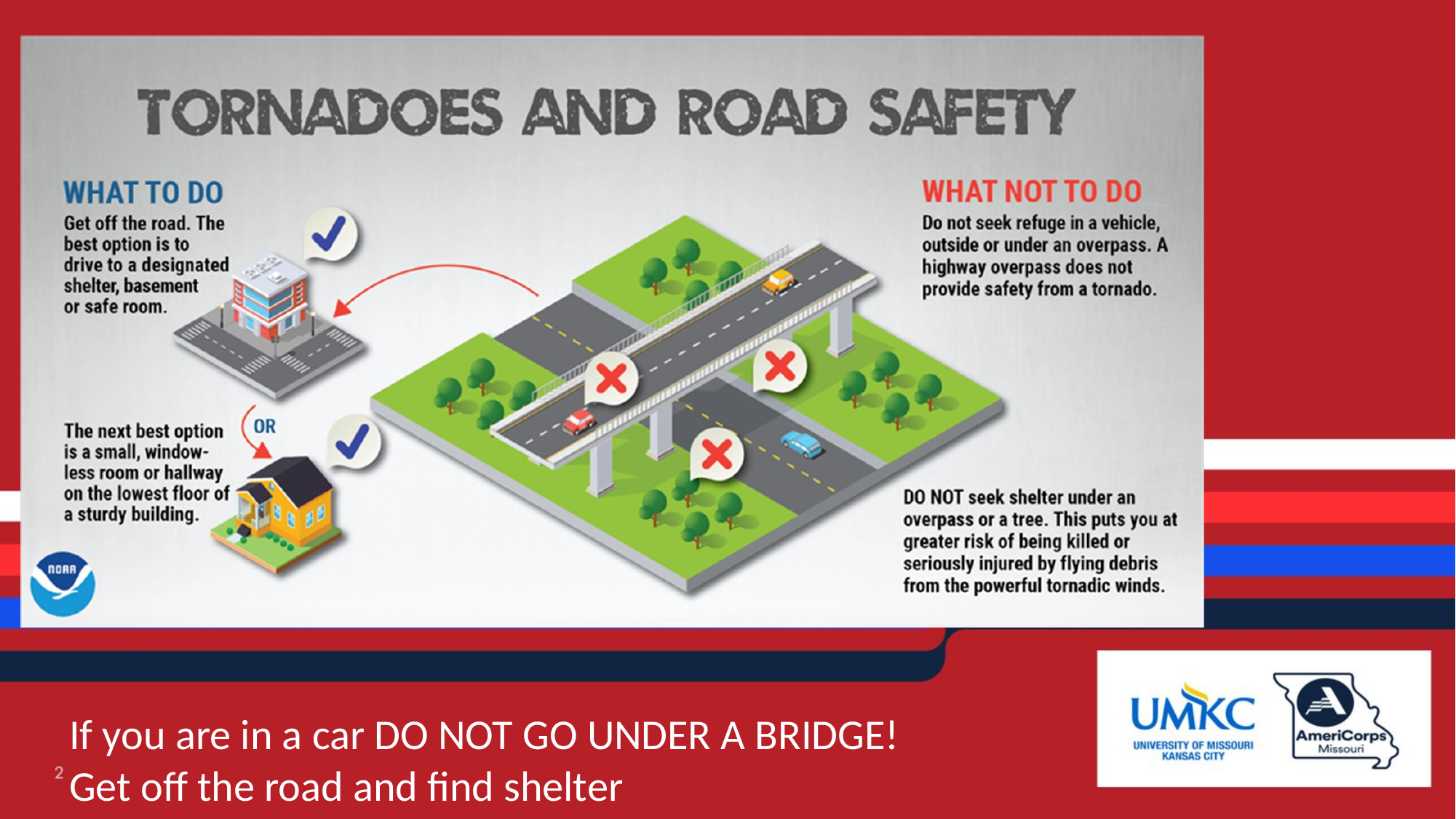

If you are in a car DO NOT GO UNDER A BRIDGE!
Get off the road and find shelter

## Slide 11
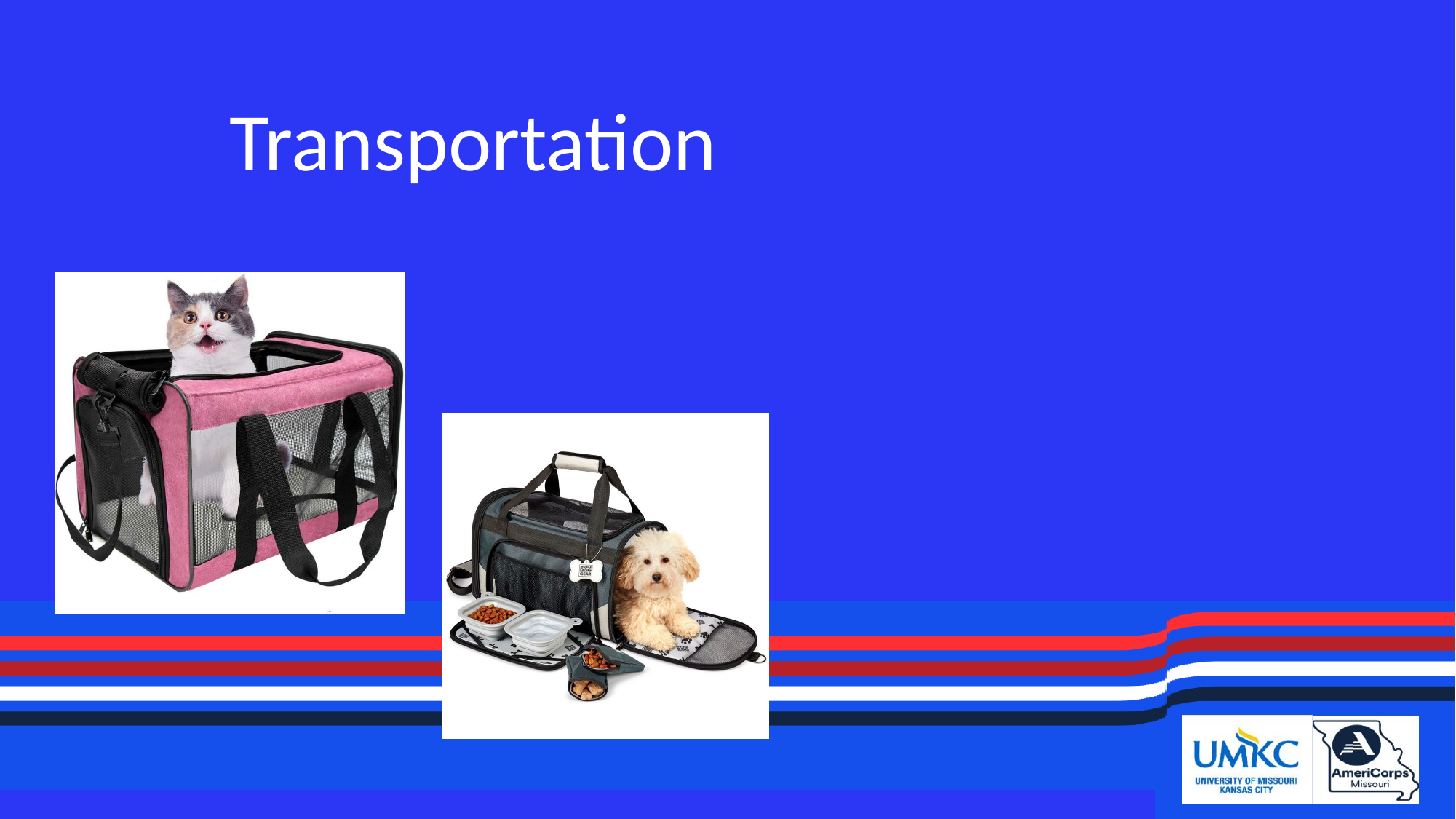

Transportation

## Slide 12
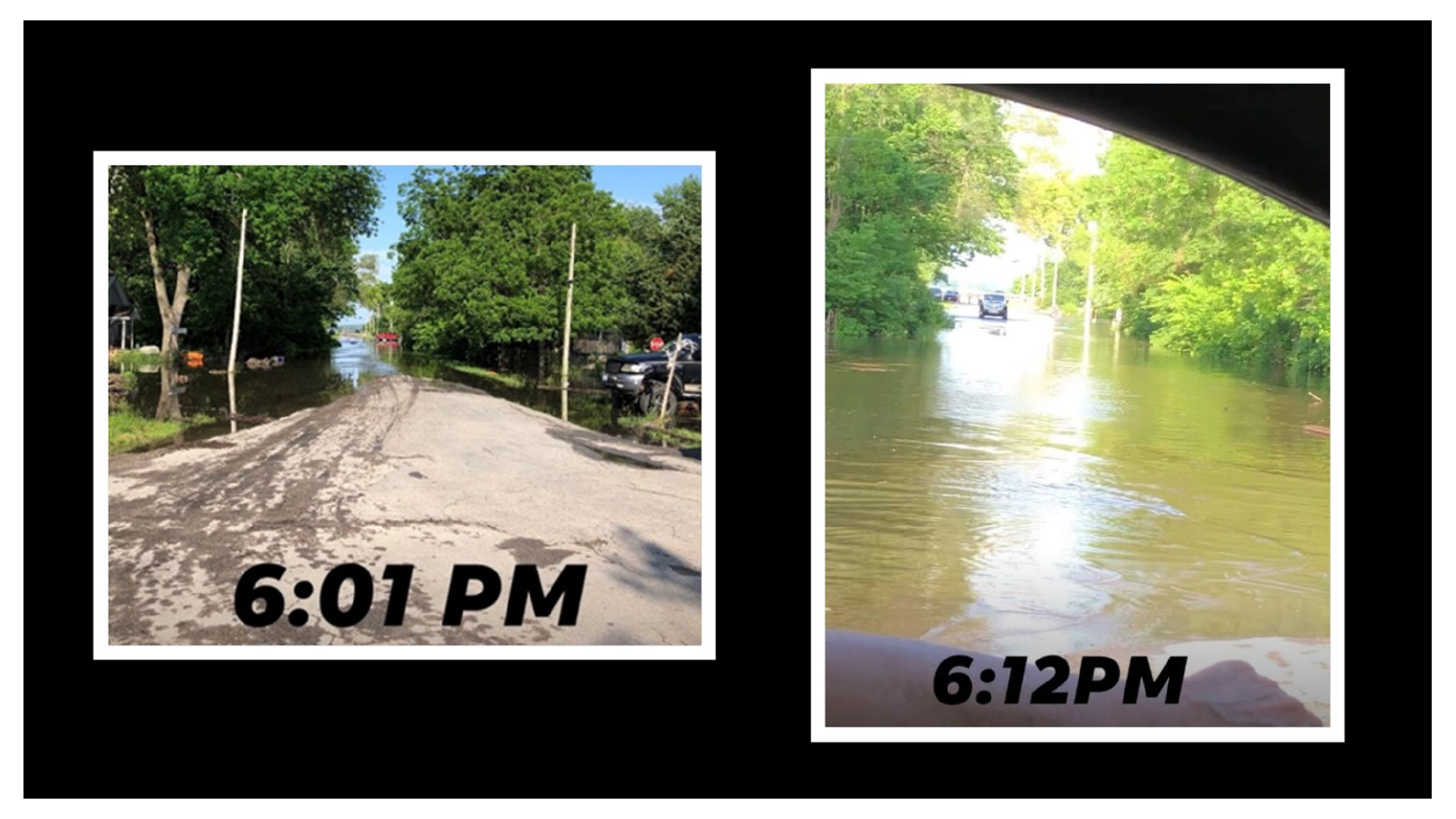

## Slide 13
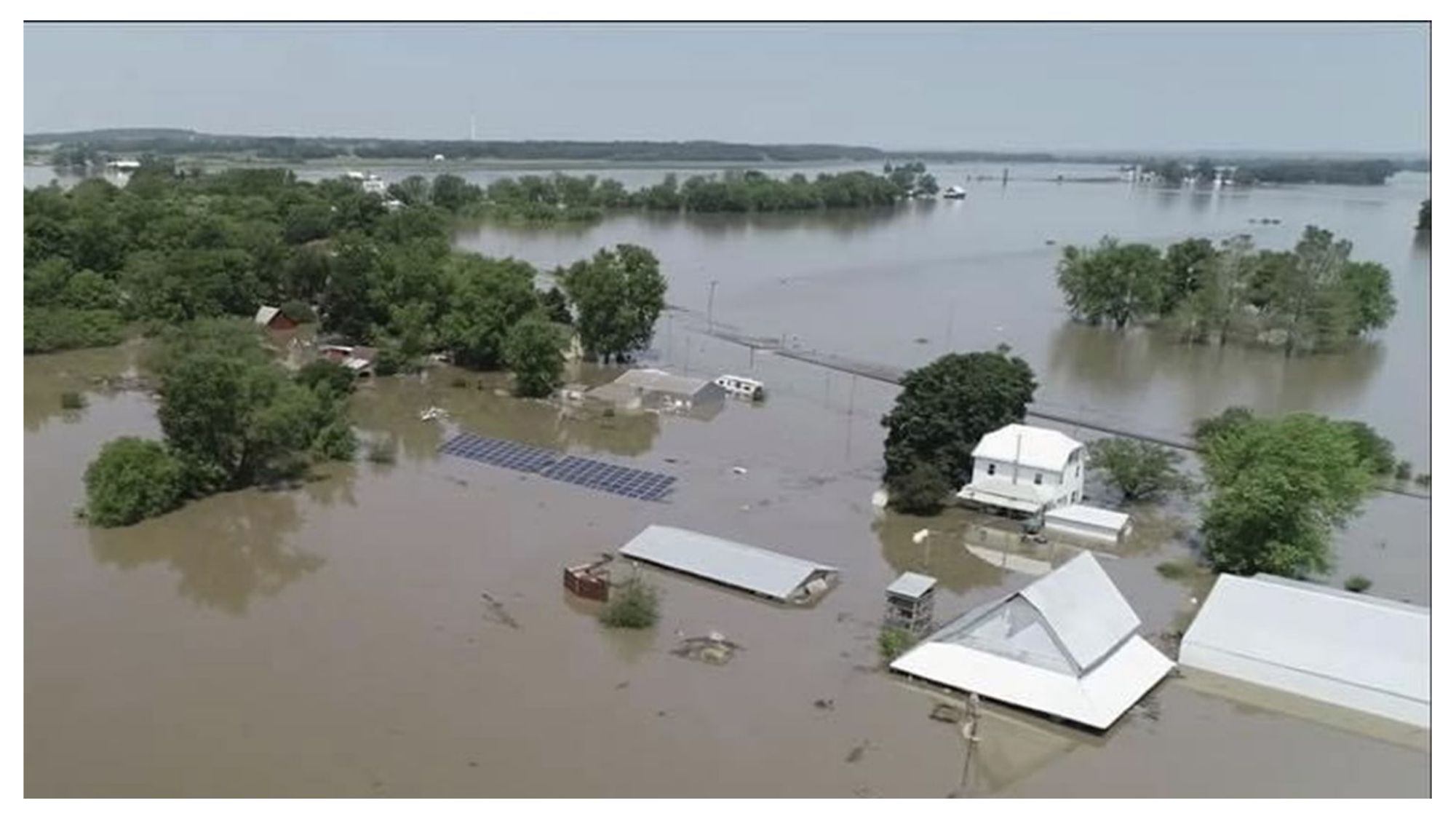

## Slide 14
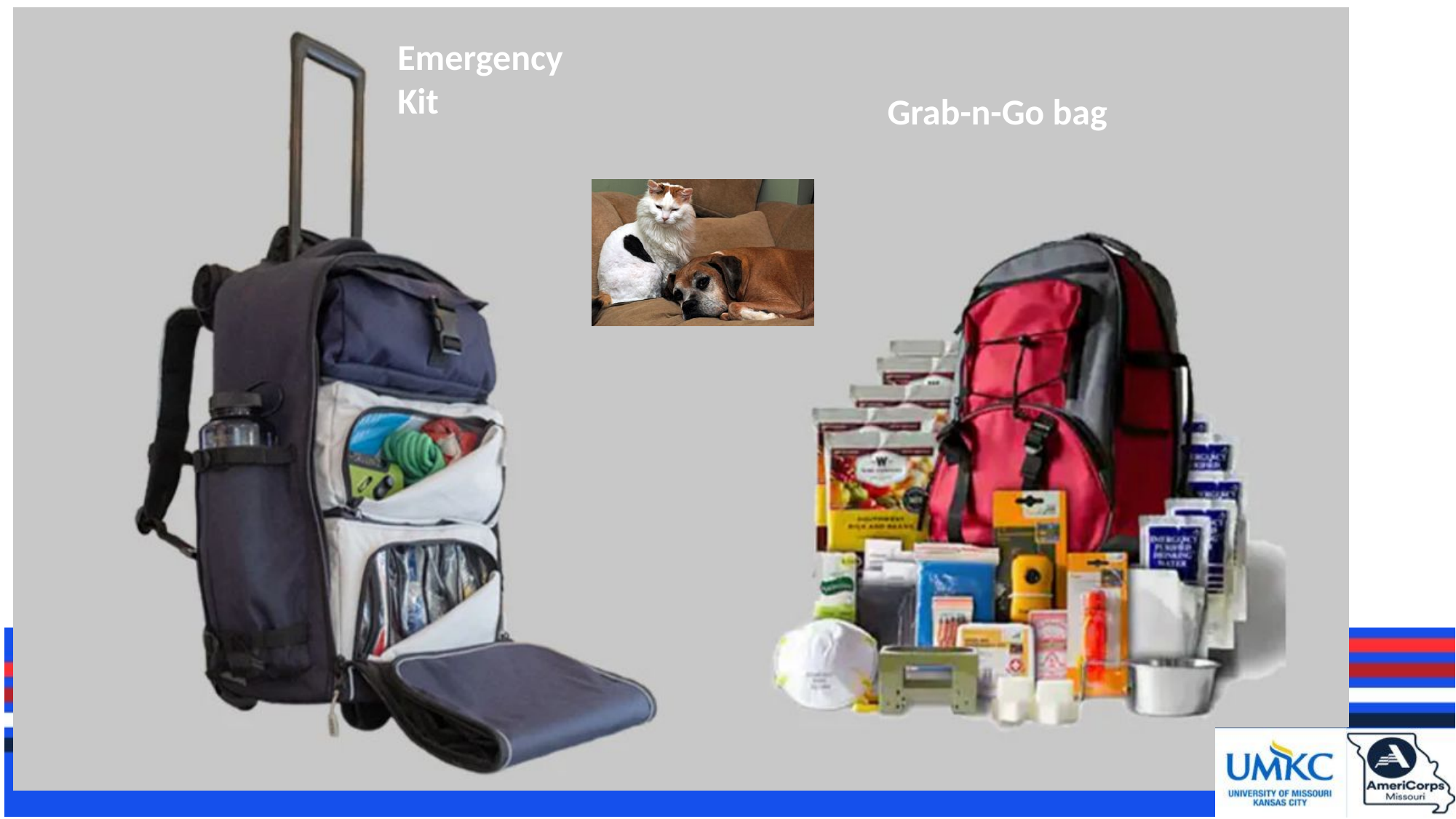

Emergency Kit
Grab-n-Go bag

## Slide 15
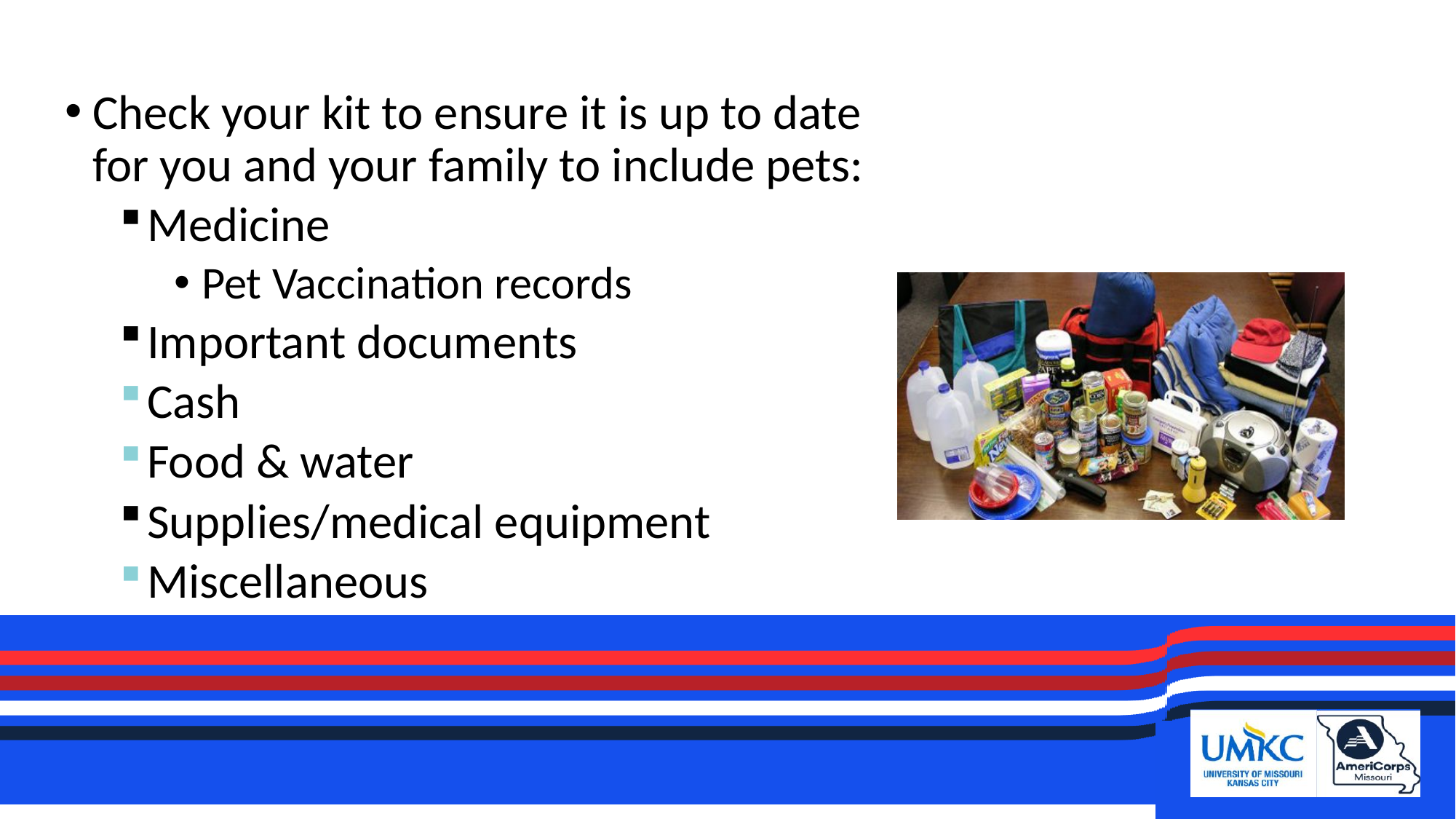

Check your kit to ensure it is up to date for you and your family to include pets:
Medicine
Pet Vaccination records
Important documents
Cash
Food & water
Supplies/medical equipment
Miscellaneous

## Slide 16
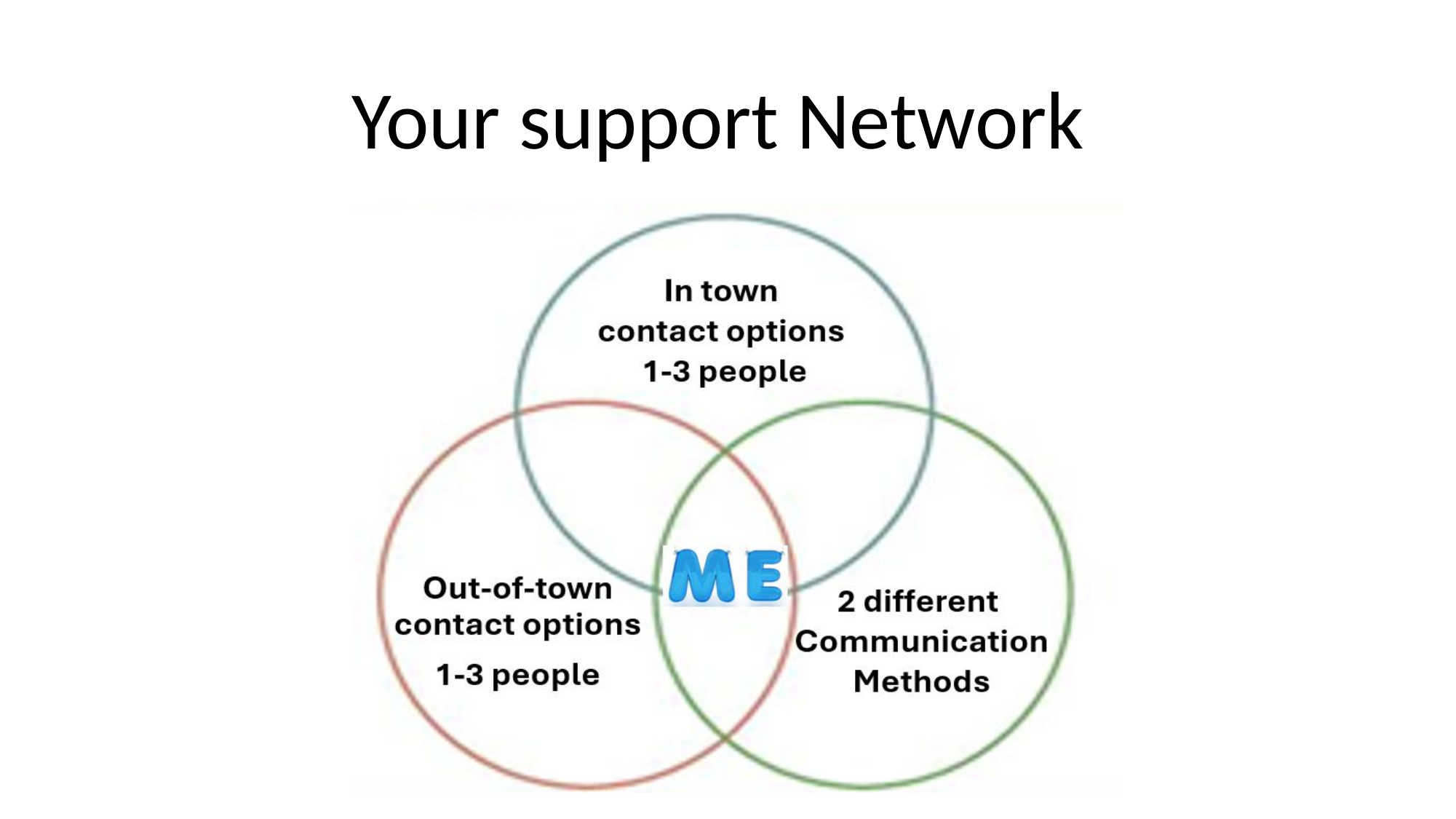

# Your support Network

## Slide 17
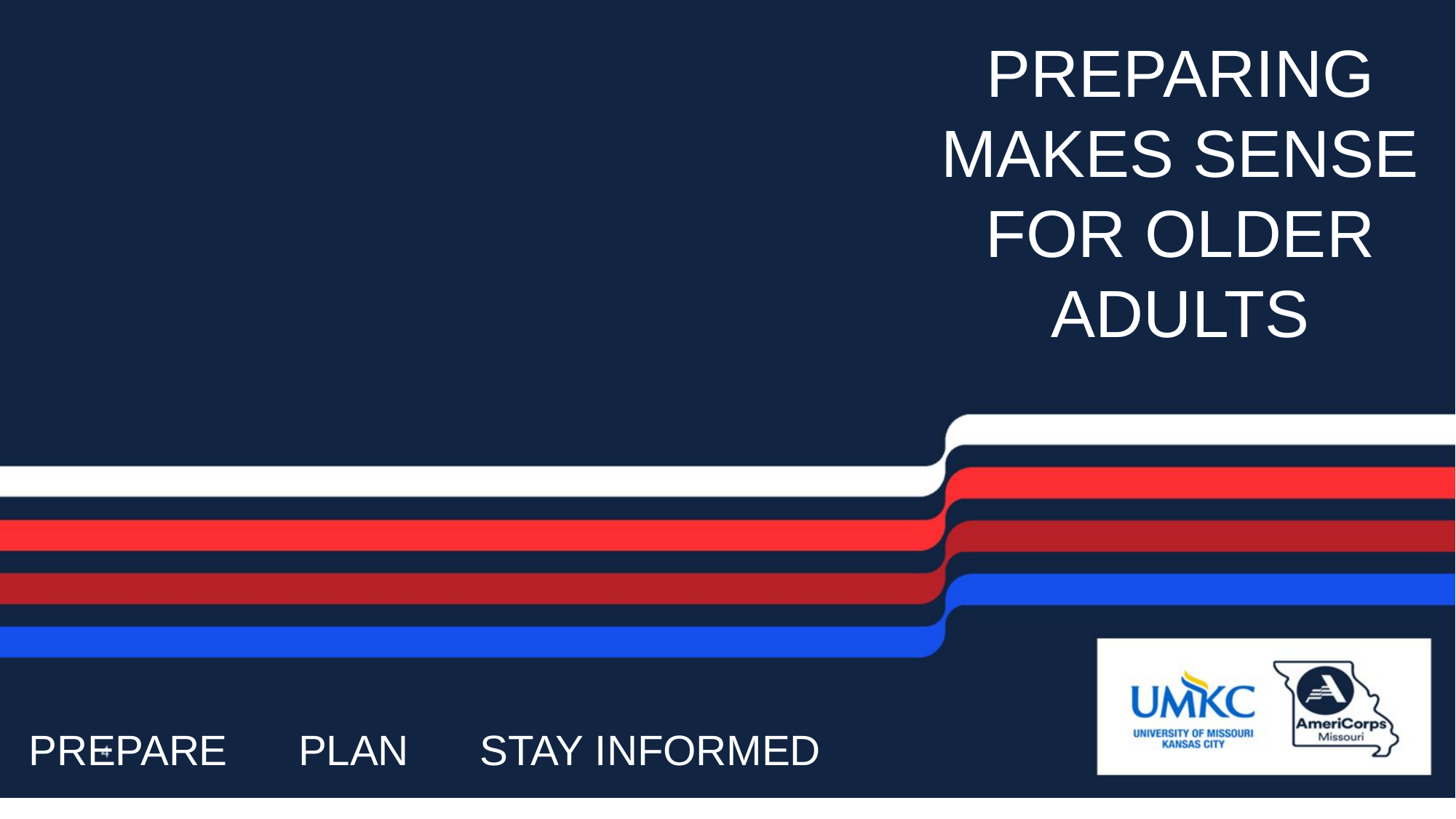

PREPARING MAKES SENSE FOR OLDER ADULTS
PREPARE PLAN STAY INFORMED

## Slide 18
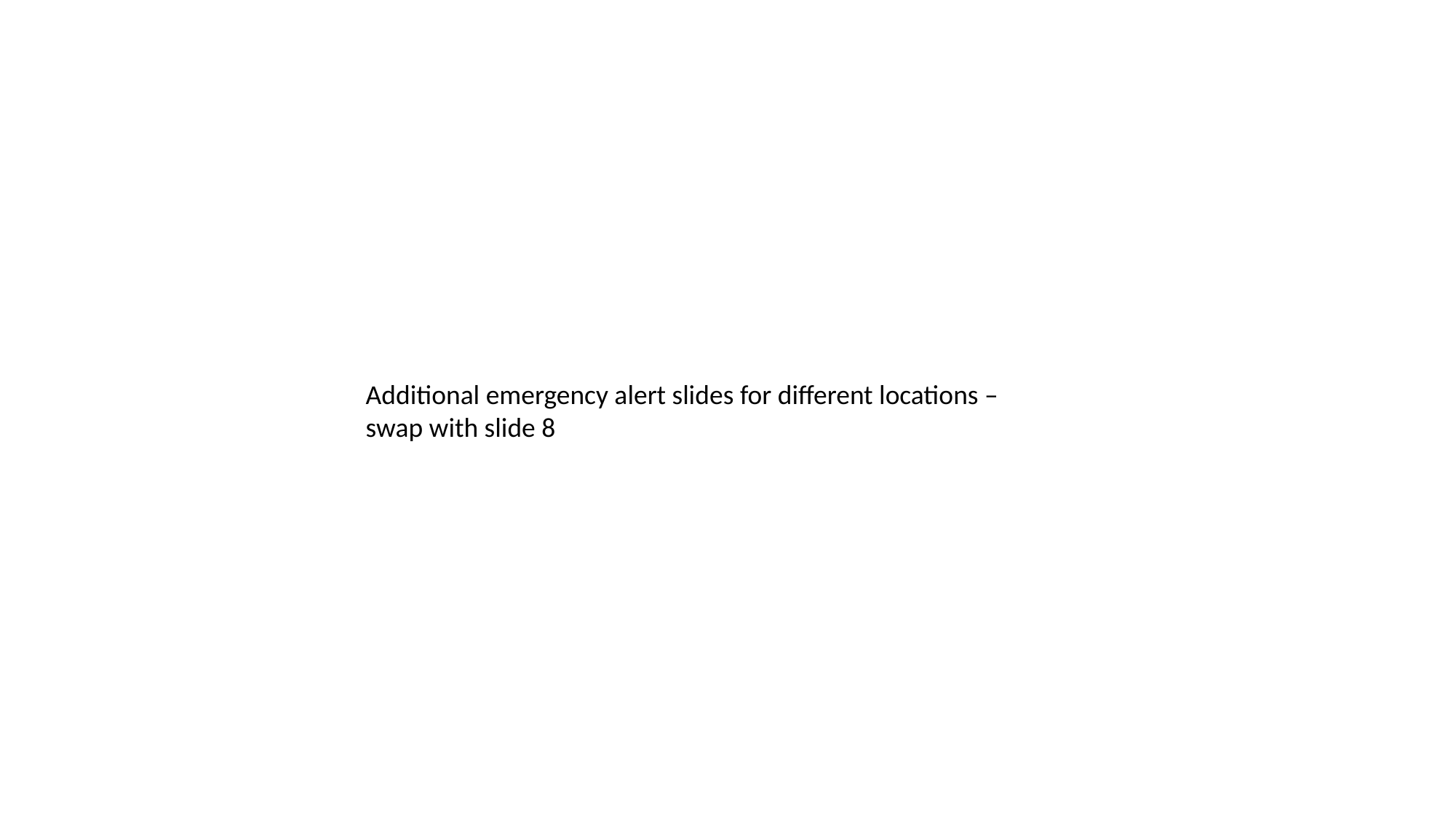

Additional emergency alert slides for different locations – swap with slide 8

## Slide 19
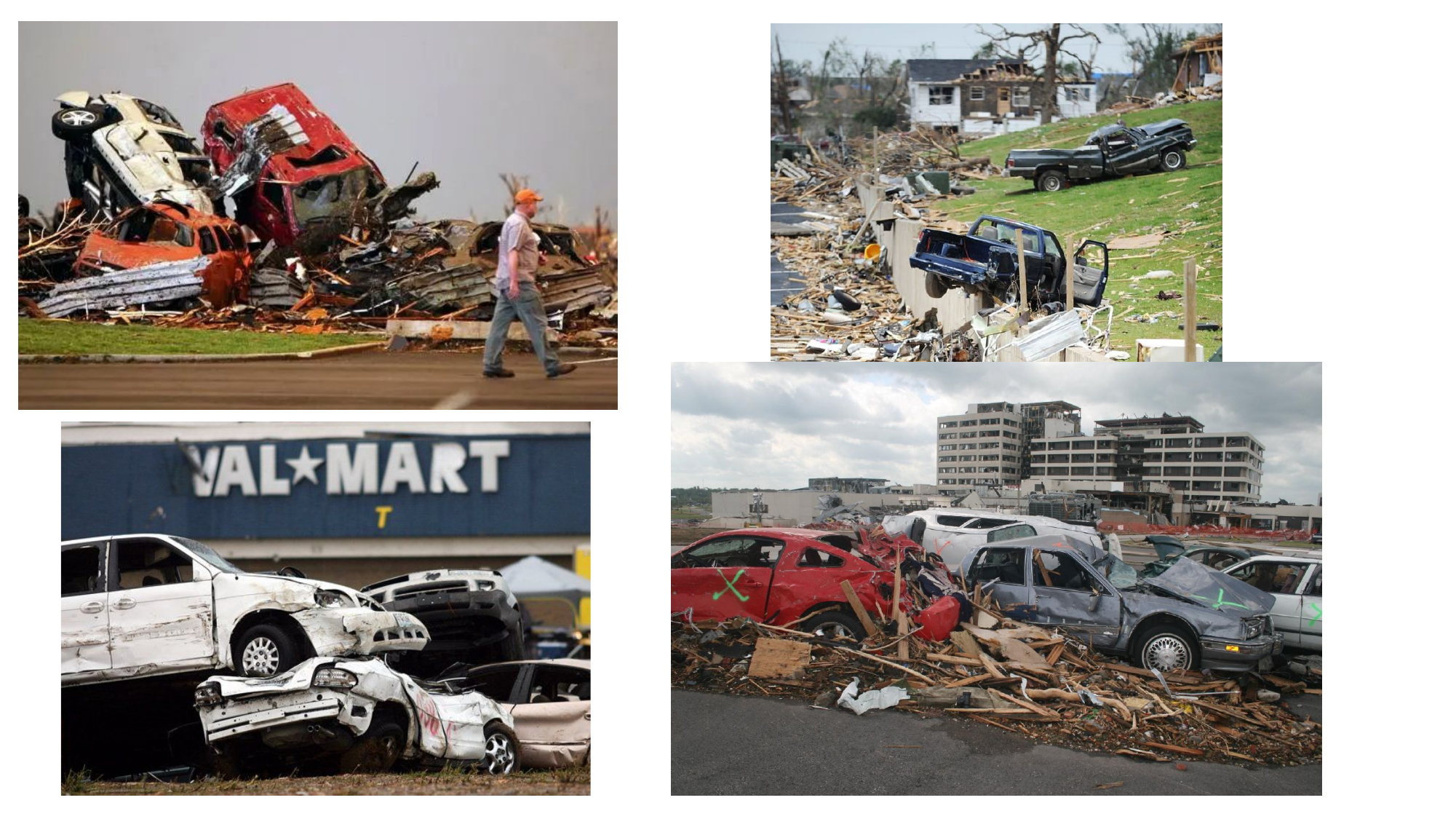

## Slide 20
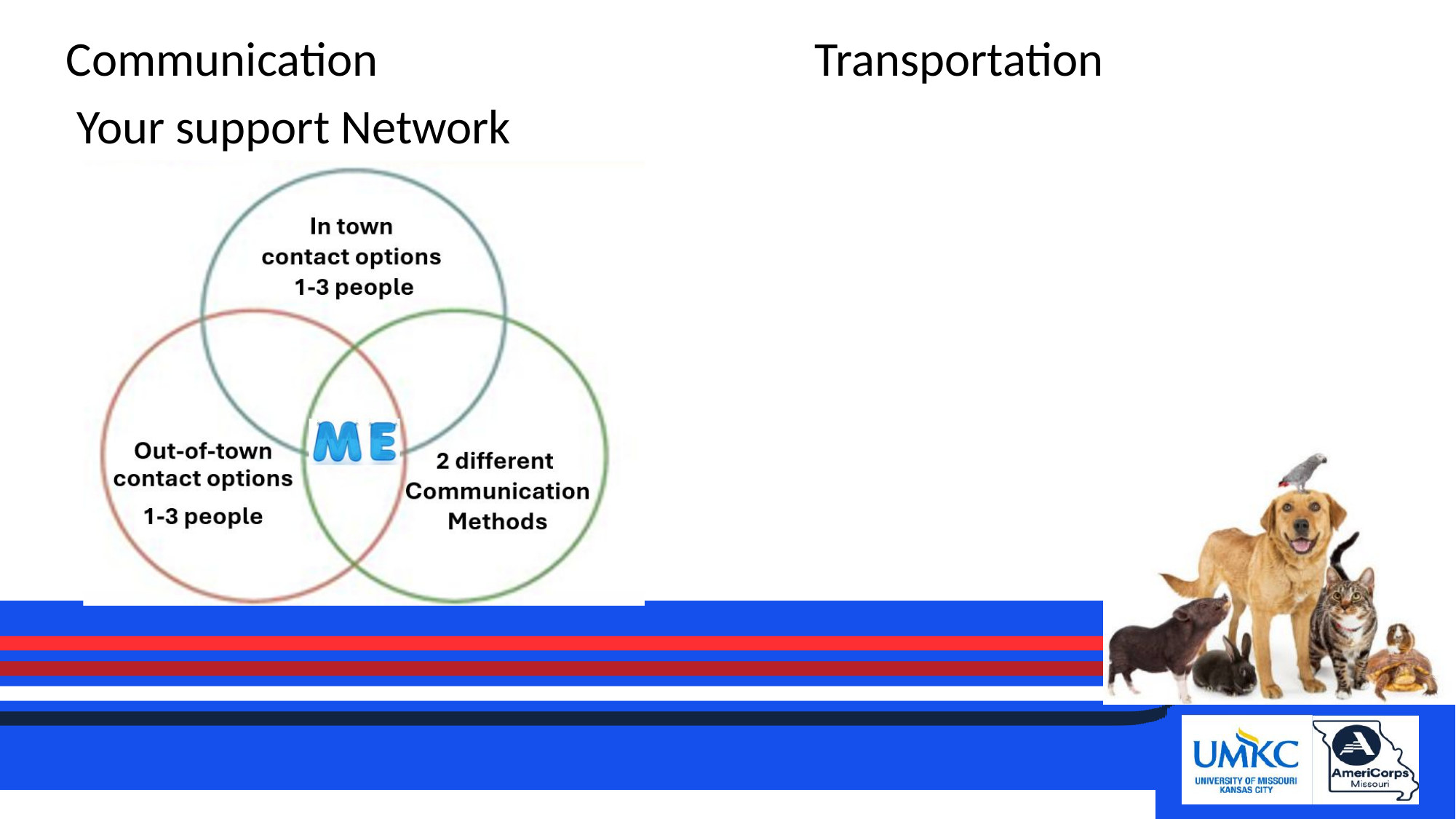

Communication                                        Transportation
 Your support Network
Prepare for action
Transportation

## Slide 21
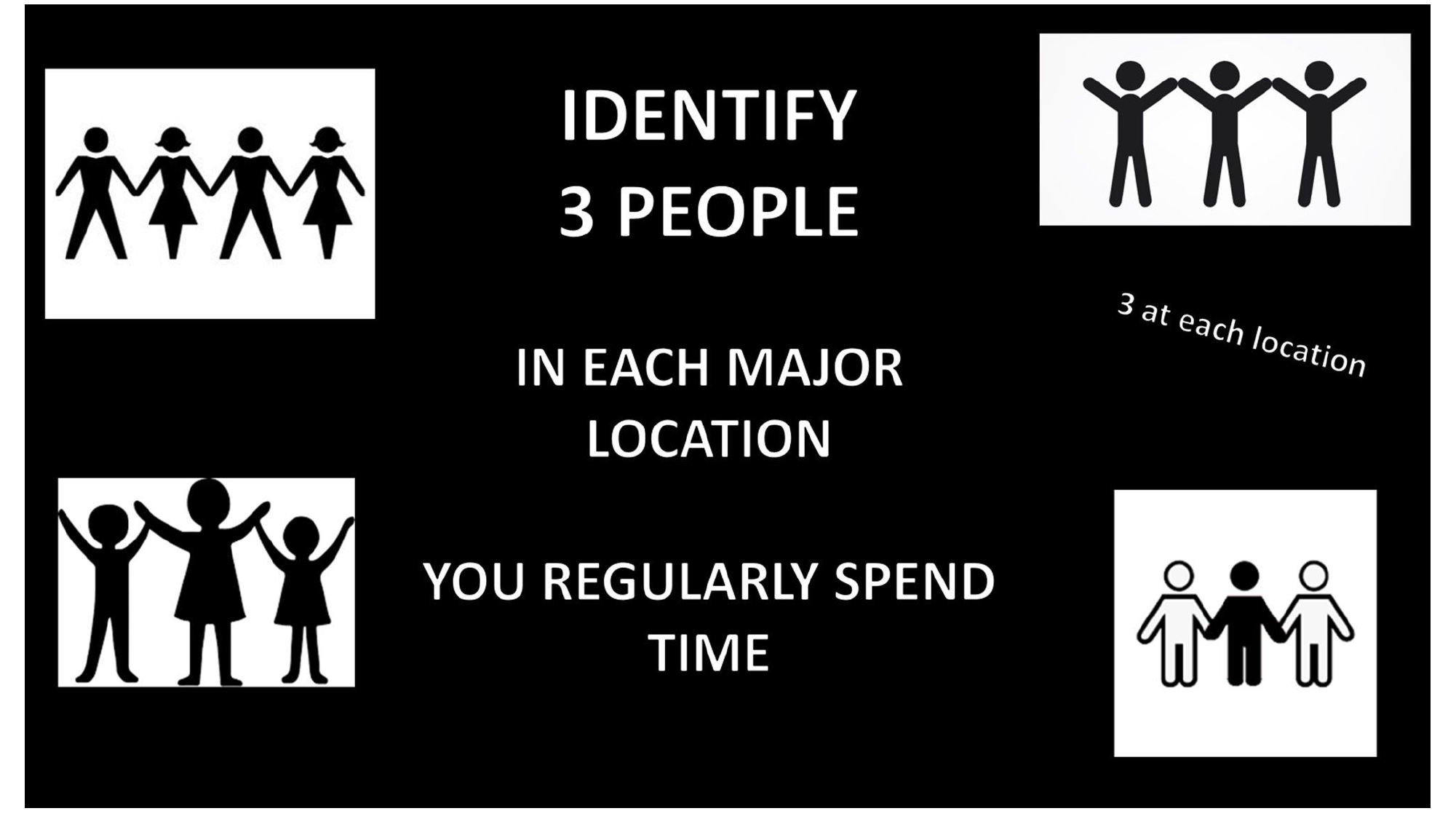

## Slide 22
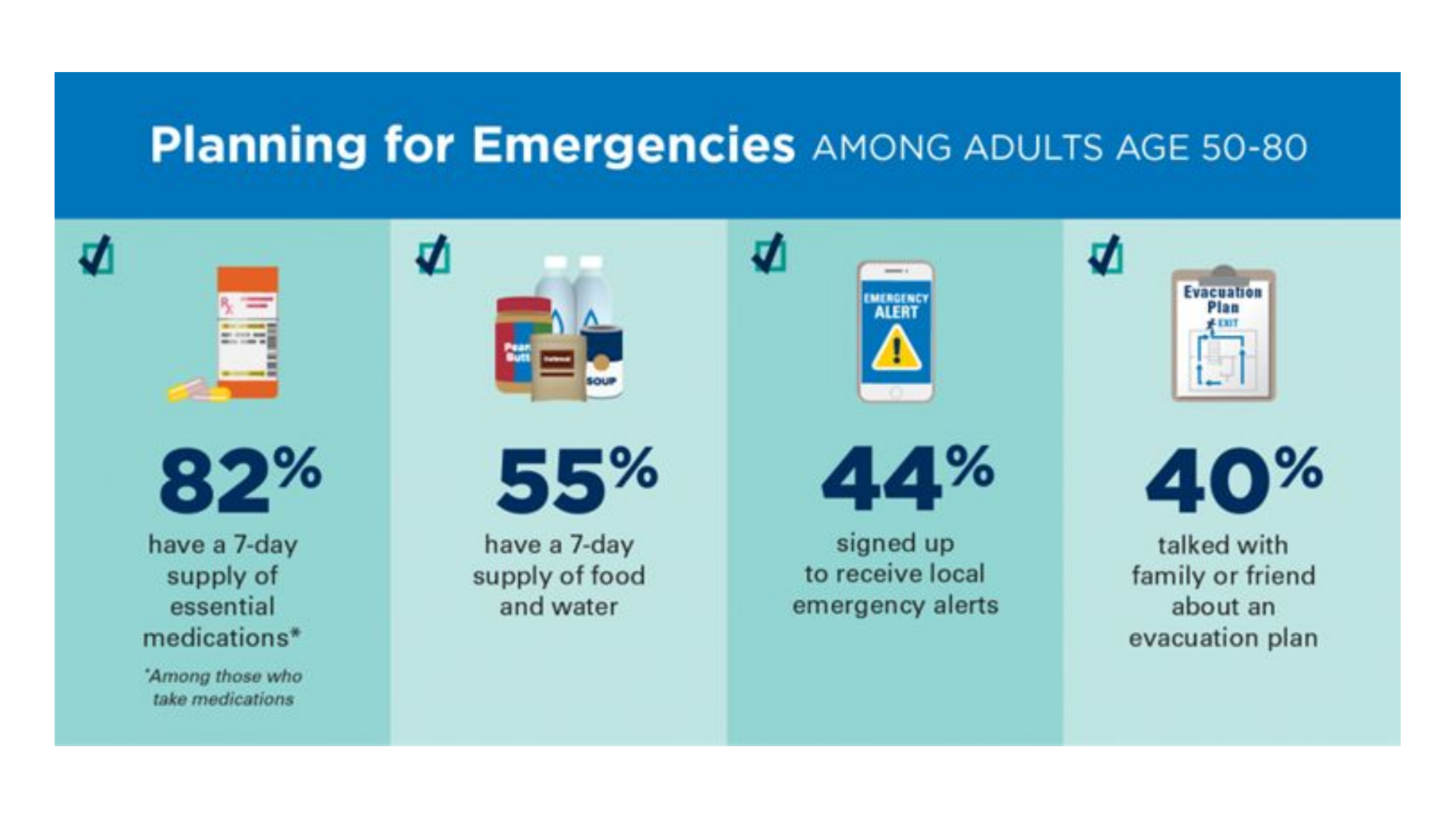

## Slide 23
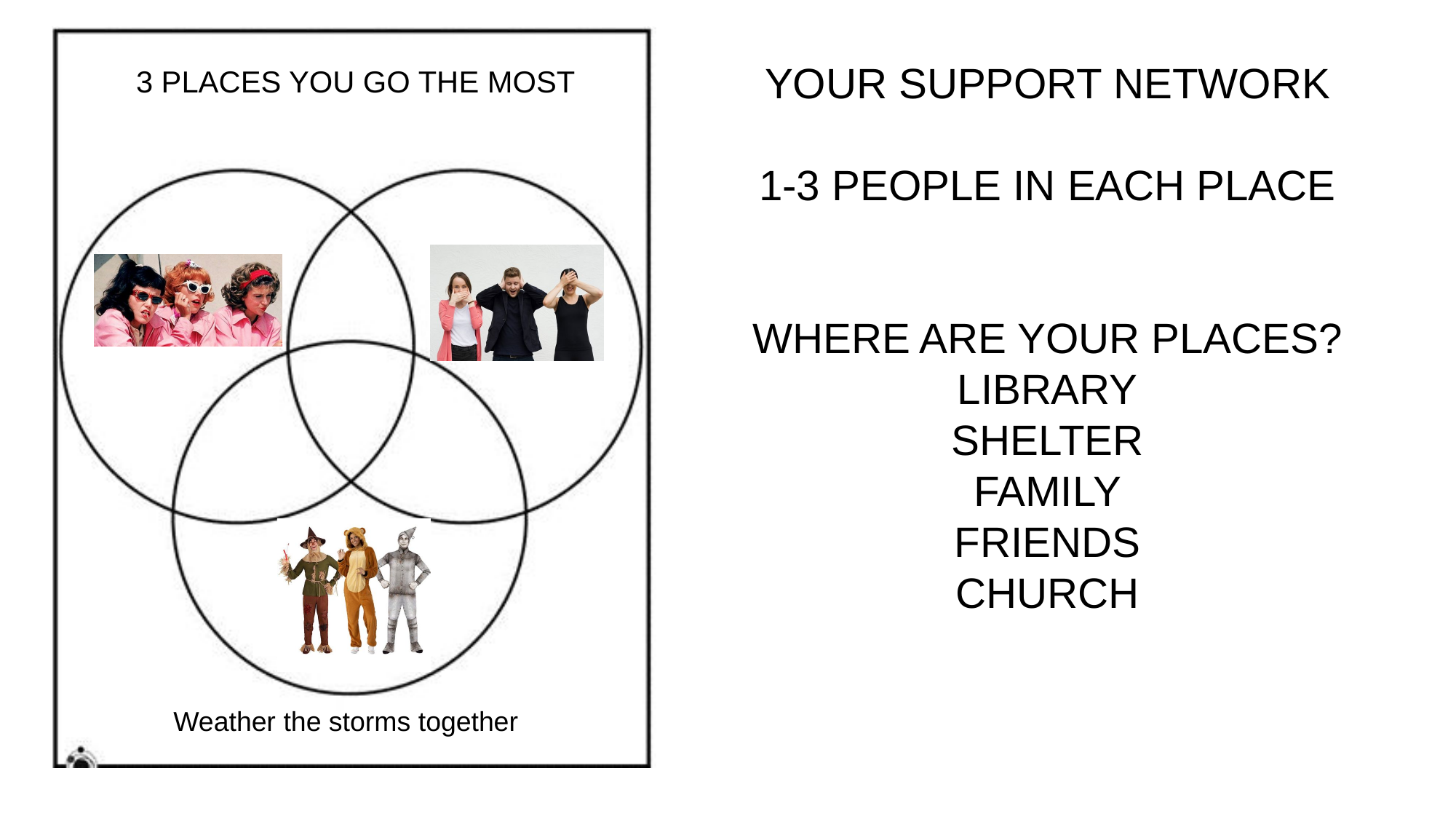

YOUR SUPPORT NETWORK
1-3 PEOPLE IN EACH PLACE
WHERE ARE YOUR PLACES?
LIBRARY
SHELTER
FAMILY
FRIENDS
CHURCH
3 PLACES YOU GO THE MOST
 Weather the storms together

## Slide 24
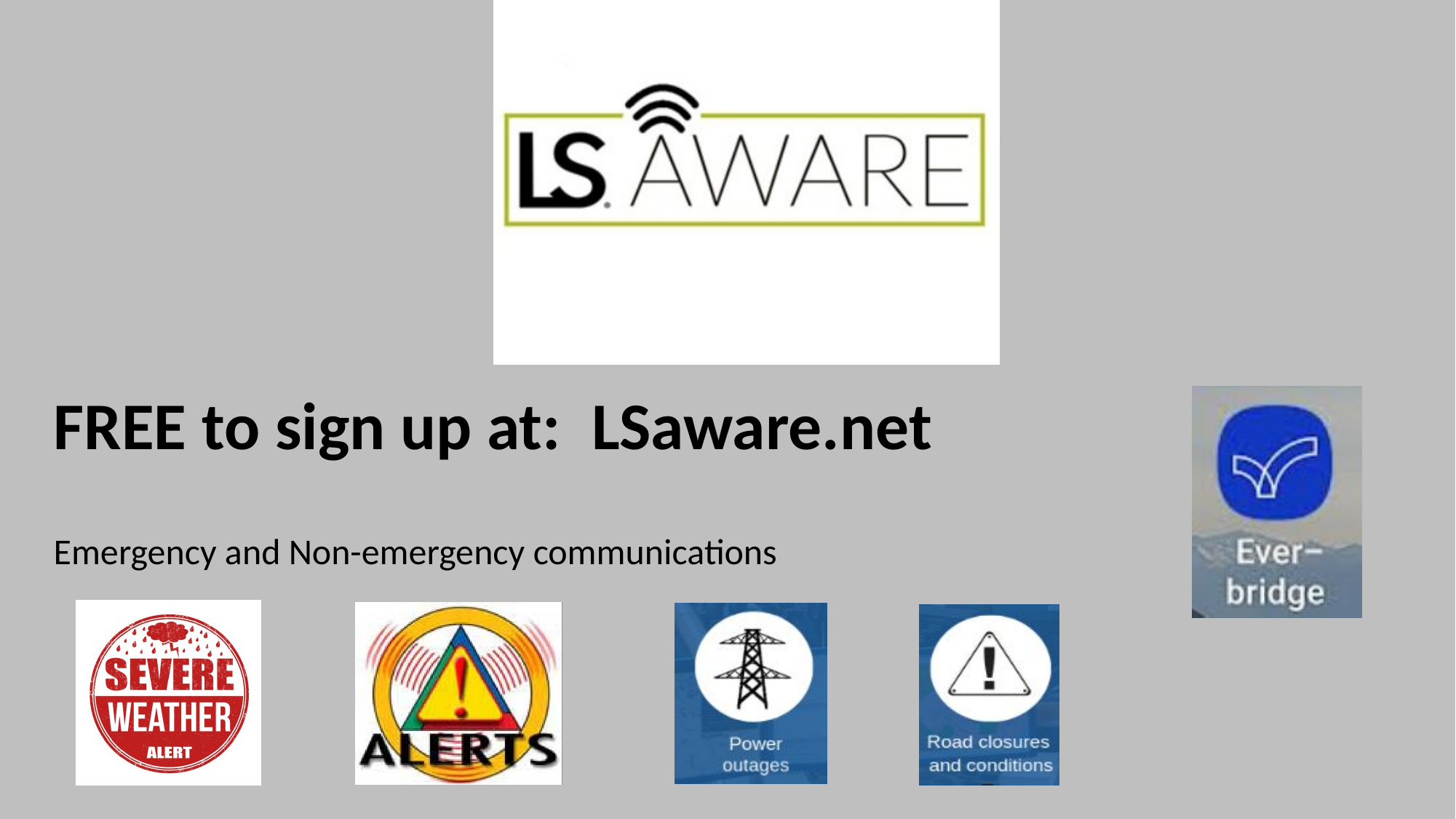

FREE to sign up at:  LSaware.net
Emergency and Non-emergency communications

## Slide 25
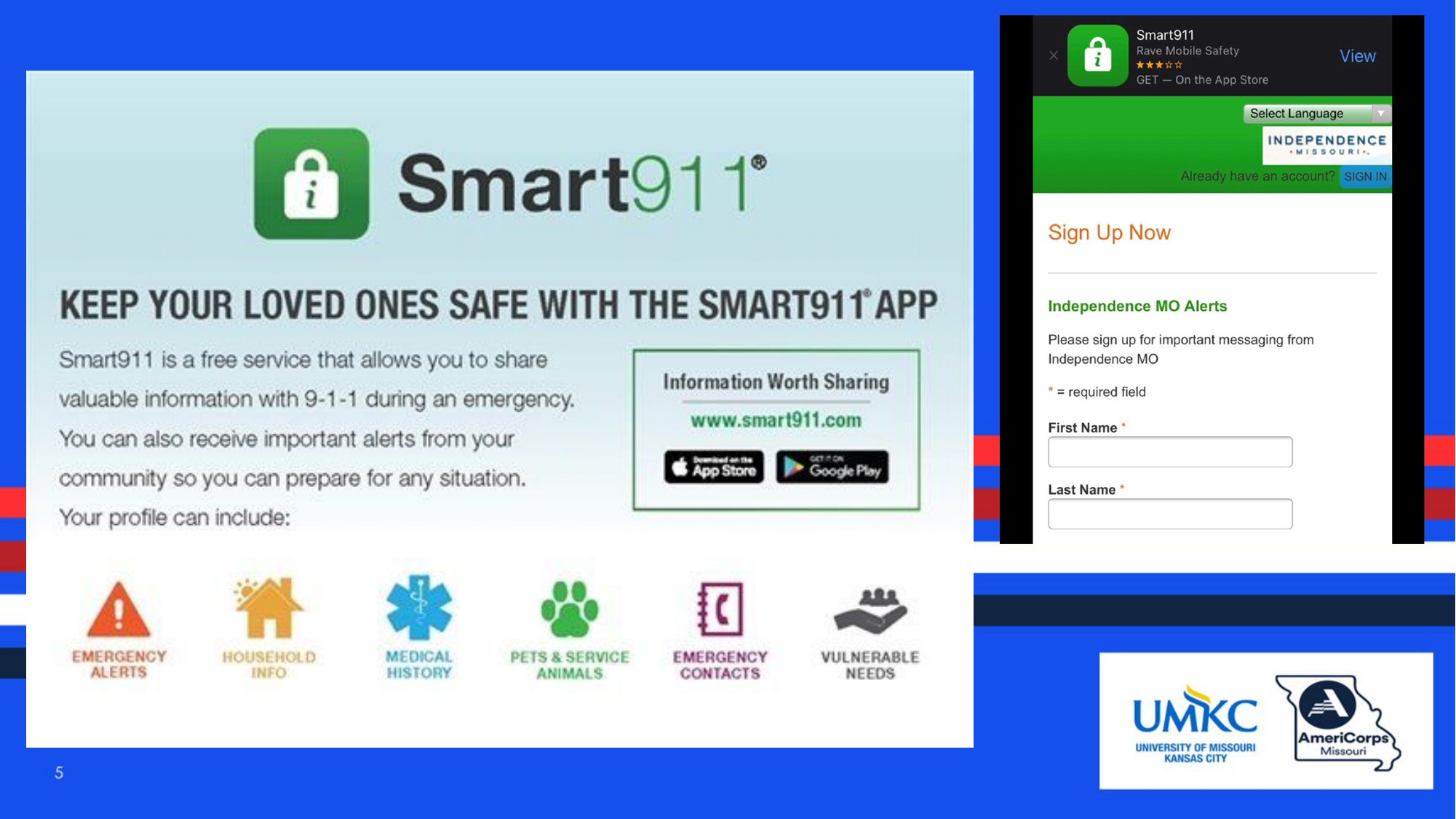

## Slide 26
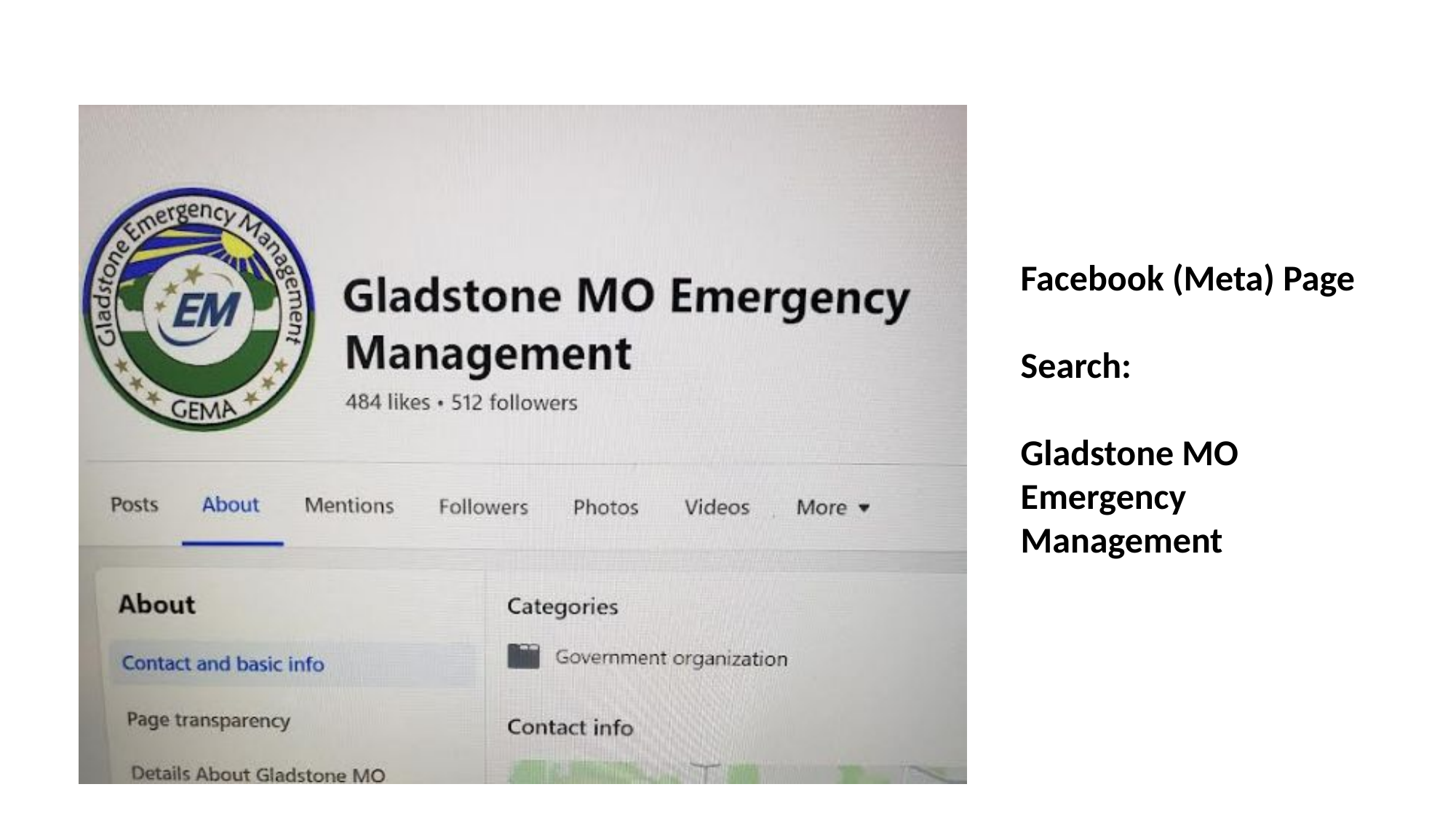

Facebook (Meta) Page
Search:
Gladstone MO Emergency Management
